# Precision cancer sono-immunotherapy using deep-tissue activatable semiconducting polymer immunomodulatory nanoparticles

**DOI:** 10.1038/s41467-022-31551-6

**Published:** 2022-07-12

**Authors:** Jingchao Li, Yu Luo, Ziling Zeng, Dong Cui, Jiaguo Huang, Chenjie Xu, Liping Li, Kanyi Pu, Ruiping Zhang

**Affiliations:** 1 School of Chemical and Biomedical Engineering, Nanyang Technological University, 70 Nanyang Drive, Singapore, 637457 Singapore; 2 School of Chemical Science and Engineering, Tongji University, 1239 Siping Road, 200092 Shanghai, China; 3 The Third Hospital of Shanxi Medical University, Shanxi Bethune Hospital, Shanxi Academy of Medical Sciences, 030032 Taiyuan, China

**Keywords:** Biomaterials, Conjugated polymers, Targeted therapies, Cancer immunotherapy

## Abstract

Nanomedicine holds promise to enhance cancer immunotherapy; however, its potential to elicit highly specific anti-tumor immunity without compromising immune tolerance has yet to be fully unlocked. This study develops deep-tissue activatable cancer sono-immunotherapy based on the discovery of a semiconducting polymer that generates sonodynamic singlet oxygen (^1^O_2_) substantially higher than other sonosensitizers. Conjugation of two immunomodulators via ^1^O_2_-cleavable linkers onto this polymer affords semiconducting polymer immunomodulatory nanoparticles (SPINs) whose immunotherapeutic actions are largely inhibited. Under ultrasound irradiation, SPINs generate ^1^O_2_ not only to directly debulk tumors and reprogram tumor microenvironment to enhance tumor immunogenicity, but also to remotely release the immunomodulators specifically at tumor site. Such a precision sono-immunotherapy eliminates tumors and prevents relapse in pancreatic mouse tumor model. SPINs show effective antitumor efficacy even in a rabbit tumor model. Moreover, the sonodynamic activation of SPINs confines immunotherapeutic action primarily to tumors, reducing the sign of immune-related adverse events.

## Abstract (web summary)

To improve the specificity of immunotherapy, here the authors report the design of a semiconducting polymer immunomodulatory nanoparticle with sonodynamic process to remotely release immune-modulators for deep-tissue precision cancer sono-immunotherapy.

## Introduction

Cancer immunotherapy that targets the host immune system for anticancer therapeutic intervention has revolutionized the paradigm of oncology^1–3^. Particularly, checkpoint blockade immunotherapy that interferes inhibitory pathways in adaptive immunity has improved patient survival across a variety of cancer types^4^, as exemplified by the clinical practice of the U.S. Food and Drug Administration (FDA)-approved antibodies that antagonize cytotoxic T-lymphocyte-associated antigen 4 (CTLA-4) and programmed death 1 (PD-1)/programmed death-ligand 1 (PD-L1)^5,6^. However, as the therapeutic index largely depends on the conditions of pre-existing immunity, many non-immunogenic “cold” tumors (e.g., glioblastomas, pancreatic, breast, and colorectal carcinoma) often manifested with a low number of tumor-infiltrating lymphocytes are poorly responsive to immune checkpoint immunotherapy (~10–30% in clinical patients)^7^. To overcome this issue, other therapeutic modalities with the ability to elicit immunogenic cell death (ICD) (such as chemotherapy, phototherapy, and radiotherapy) can be combined with checkpoint blockade immunotherapy, which convert “cold” tumors into immunogenic “hot” phenotypes and sensitize tumors for improved immunotherapy^8^. However, these combinational immunotherapies inevitably encounter increased risk of immune-related adverse events (irAEs) due to enhanced disruption in homeostatic immune tolerance relative to monotherapy^9,10^.

Nanomedicine has the potential to enhance immunotherapy and simultaneously to reduce irAEs^11^. Local administration of immunotherapeutic hydrogels allows slow release of immune checkpoint blockers, cytokines or immune cells in the tumor microenvironment to induce regional responses^12–15^, which however is difficult to treat invisible or inaccessible tumors^16^. Alternatively, nanoparticles that release immunotherapeutic agents in response to the tumor biomarkers can be systemically administered, which still permits relatively controlled immunotherapy to tumor sites^17–20^. However, their strong reliance on the different levels of the selected biomarker between tumors and normal tissues limits the regional selectivity of immune action and constrains the design flexibility to the availability of tumor-specific biomarkers^21^. In contrast to endogenous biomarkers, exogenous stimuli bypass both limitations^22^, and can provide a more precise way to remotely control immune activation in tumors. Although immunotherapeutic nanoagents responsive to near-infrared (NIR) light have been developed for immunotherapy in a more safe and efficient manner^23–26^, light often shows shallow tissue penetration (<1 cm)^27,28^. By contrast, ultrasound (US) has deep-tissue penetration and the ability to induce the generation of reactive oxygen species (ROS) from sonosensitizers for cancer therapy^29–32^. However, such a sonodynamic process has not been integrated into nanomedicines as the exogenous stimulus to gain the spatiotemporal control of immunotherapy in preclinical settings.

**Fig. 1:**
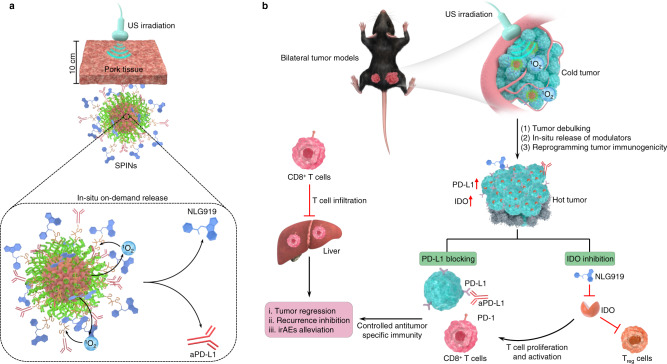
Design and mechanism of SPINs for deep-tissue activatable sono-immunotherapy. **a** Schematic illustration of US-triggered deep-tissue activation of SPINs to release immunomodulators. **b** Schematic illustration of sonodynamic activation of SPINs to debulk tumor, enhance tumor immunogenicity, and release immunomodulators in situ as well as synergetic action of IDO inhibition and PD-L1 blocking on enhancing antitumor immunity with alleviated irAEs relative to free-drug administration.

We herein report the development of semiconducting polymer nanoparticles (SPNs) for deep-tissue activatable cancer sono-immunotherapy. By screening a library of agents, an ideal SPN is identified to have the highest sonodynamic singlet oxygen (^1^O_2_) generation efficiency. This SPN is used to further construct semiconducting polymer immunomodulatory nanoparticles (SPINs) by conjugating immunomodulators via a ^1^O_2_-cleavable linker to the backbone of SPNs (Fig. 1a). The antitumor immunity of SPINs is tested against the subcutaneous pancreatic mouse tumors, as the pancreatic tumor is one of the most aggressive tumors with low immunogenicity, high metastasis, and low survival rate^33^. SPINs can effectively accumulate into mouse subcutaneous pancreatic tumors and rabbit orthotopic pancreatic tumors after systemic administration. Under US irradiation directed to the tumor regions, SPINs generate ^1^O_2_ that can (i) debulk tumor tissues, (ii) reprogram the tumor microenvironment through upregulating the expression levels of PD-L1 and indoleamine 2,3-dioxygenase (IDO), and (iii) scissor the ^1^O_2_-cleavable linkers to remotely release the immunomodulators, NLG919 and anti-PD-L1 antibody (aPD-L1). NLG919 is a potent inhibitor for IDO and can promote the proliferation of cytotoxic T lymphocytes (CTLs) and inhibit regulatory T cells (T_reg_)^34^. The aPD-L1 can bind to PD-L1 on the surface of cancer cells to block its binding with PD-1 on the surface of PD-1^+^ T cells^35^, thereby inhibiting T-cell exhaustion and in turn promoting the tumoricidal functions of T-cell immunity^36^. In addition, NLG919 has shown a great potential to improve the efficacy of aPD-L1, achieving a synergistic effect^37^. In such a way, sonodynamic activation cascade precisely restricts the immunotherapeutic action to tumor regions. Thus, SPIN-mediated activatable sono-immunotherapy not only elicits high antitumor immunity to reject both primary and metastatic distant pancreatic tumors and prevent tumor recurrence, but also reduces the probability of irAEs (Fig. 1b). SPIN-mediated deep-tissue therapy of pancreatic tumors covered with 5-cm tissues and deep-seated rabbit orthotopic pancreatic tumors is also achieved.

## Results

### Screening of SPNs for sonodynamic ^1^O_2_ generation

**Fig. 2:**
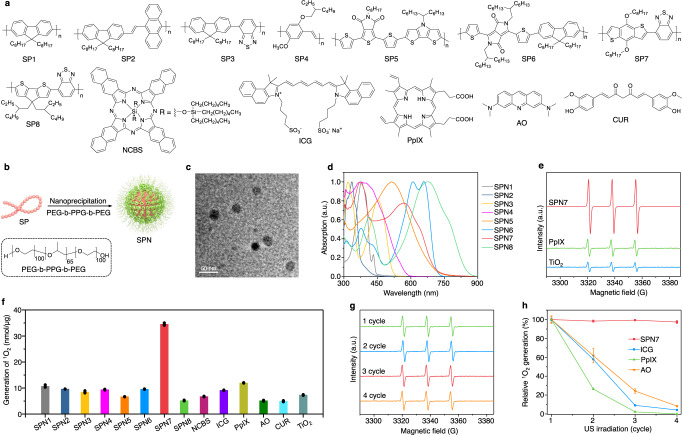
Screening of SPNs for sonodynamic therapy. **a** Chemical structures of SPs and small molecular sonosensitizers. **b** Schematic illustration of the synthesis of SPNs via nanoprecipitation. **c** Representative TEM image of SPN7. The experiment was repeated independently three times with similar results. **d** UV–vis absorption spectra of SPNs in 1× phosphate-buffered saline (PBS) solution (pH = 7.4). **e** ESR spectra of SPN7, PpIX and TiO_2_ nanoparticles (20 µg/mL) after US irradiation using TEMP as the trap. **f** Quantification of sonodynamic ^1^O_2_ generation for each sample (20 µg/mL) (*n* = 3). **g** ESR spectra of SPN7 (20 µg/mL) after 1, 2, 3, and 4 cycles of US irradiation with TEMP as the trap. **h** Relative ^1^O_2_ generation for SPN7, ICG, PpIX and AO (20 µg/mL) after different cycles of US irradiation (*n* = 3). US-irradiation conditions: 1.0 MHz, 1.2 W/cm^2^, 50% duty cycle, 5 min for each cycle. Data are presented as mean values ± SD. Source data are provided as a Source Data file.

A library of hydrophobic semiconducting polymers (SPs) (Fig. 2a) were transformed into water-soluble nanoparticles via nanoprecipitation with the assistance of an amphiphilic triblock copolymer, poly(ethylene glycol)-block-poly-(propylene glycol)-block-poly(ethylene glycol) (PEG-b-PPG-b-PEG) (Fig. 2b). As shown in the transmission electron microscopy (TEM) image, the prepared SPNs exhibited a spherical morphology with a uniform size distribution (Fig. 2c). Dynamic light scattering (DLS) measurements revealed that the hydrodynamic diameters of SPNs ranged from ~24 to 40 nm (Supplementary Fig. 1). Owing to the different molecular structures, these SPNs had the absorption maxima ranging from 320 to 680 nm (Fig. 2d), and fluorescence emission maxima ranging from 430 to 840 nm (Supplementary Fig. 2).

The sonodynamic ^1^O_2_ generation properties of SPNs were investigated and compared with other commonly used sonosensitizers including silicon 2,3-naphthalocyanine bis(trihexylsilyloxide) (NCBS), indocyanine green (ICG), protoporphyrin IX (PpIX), acridine orange (AO), curcumin (CUR), and inorganic titanium dioxide (TiO_2_) nanoparticles at the same mass concentration. Electron spin resonance (ESR) was applied to measure the generation of ^1^O_2_ under US irradiation using 2,2,6,6-tetramethylpiperidine (TEMP) as the radical trap. The characteristic ESR peak of ^1^O_2_ was observed for all samples (Fig. 2e and Supplementary Fig. 3); however, SPN7 generated the highest amount of ^1^O_2_ (34.5 nmol/μg), which was at least threefold higher relative to the others (Fig. 2f). The ranking was consistent with the ^1^O_2_ generation efficiencies under white light irradiation (Supplementary Fig. 4). This implied that sonoluminescence-induced ^1^O_2_ generation probably was the main mechanism involved for SPNs: US induced the sonoluminescence of water in the range of 350–650 nm^38^, which subsequently excited the agents to produce ^1^O_2_ in a way similar to photodynamic process.

In addition to the efficient ^1^O_2_ generation, the intensity of ^1^O_2_ peak for SPN7 remained nearly the same (Fig. 2g) after four cycles of US irradiation (5 min for each cycle); in contrast, the ability to generate ^1^O_2_ for ICG, PpIX, and AO was almost completely demolished (dropped by 95.8, 100, and 91.7%, respectively) (Fig. 2h and Supplementary Fig. 5). This was attributed to the high stability of SPN7 under US irradiation as witnessed by its unchanged absorption, while ICG, PpIX, and AO were molecularly damaged after US irradiation (Supplementary Fig. 6). These data confirmed that SPN7 is an optimal sonosensitizer for in vivo applications.

### Construction of SPINs and deep-tissue sonodynamic activation

**Fig. 3:**
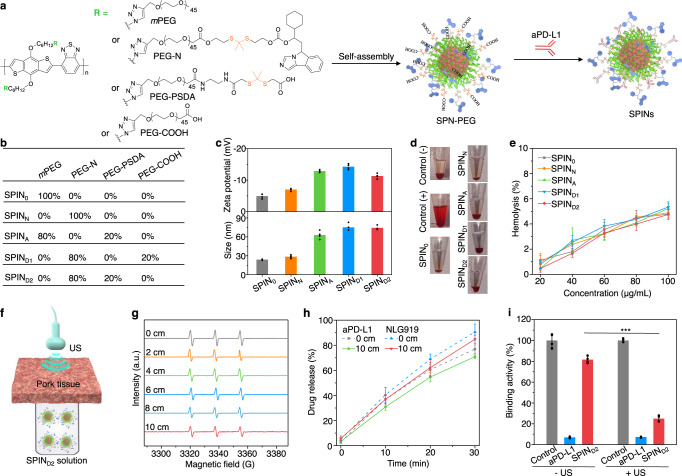
Synthesis, characterization, and sonodynamic activation of SPINs. **a** Chemical structures of amphiphilic semiconducting polymeric modulators and schematic illustration of their self-assembly and surface modification to form SPINs. **b** The molar ratios of each component in different SPINs. **c** Zeta potentials and hydrodynamic sizes of different SPINs in 1× PBS buffer (pH = 7.4) (*n* = 4). **d** Photographs of erythrocytes after incubation with 1× PBS buffer (negative control), 1% Triton X-100 (positive control), and 1× PBS buffer containing SPINs at the concentration of 100 µg/mL for 2 h, followed by centrifugation. **e** Hemolysis percentages of erythrocytes after incubation with SPINs at different concentrations for 2 h (*n* = 4). **f** Schematic illustration of US irradiation of SPIN_D2_ solutions covered with a pork tissue. **g** ESR spectra of ^1^O_2_ for SPIN_D2_ (20 µg/mL) after US irradiation (1.2 W/cm^2^, 3 min) without or with coverage of pork tissues at different thicknesses. **h** Release profiles of aPD-L1 and NLG919 from SPIN_D2_ (40 µg/mL) after US irradiation for different time (*n* = 4). **i** PD-L1/PD-1 binding activity assay after treatment with free aPD-L1 or SPIN_D2_ (40 µg/mL) with or without US irradiation (*n* = 4). SPIN_D2_ – US versus SPIN_D2_ + US: *P* < 0.0001. Statistical significance was calculated via a two-tailed Student’s *t* test. ***P < 0.001. In (**g**–**i**), the power intensity of US irradiation was 1.2 W/cm^2^ (1.0 MHz, 50% duty cycle). Data are presented as mean values ± SD. Source data are provided as a Source Data file.

SPINs were constructed based on SP7 due to its highest ^1^O_2_ generation efficacy among tested SPNs and excellent sonodynamic stability of SPN7. SP7 with bromide groups on the side chains (SP7-Br) was synthesized via Suzuki polycondensation and then reacted with sodium azide to afford azide groups linked polymer (SP7-N_3_) (Supplementary Figs. 7–10). The molecular weight of the synthesized SP7-N_3_ was measured to be 7200 Da, and the maximum absorbance and fluorescence emission was observed at 574 and 770 nm, respectively (Supplementary Fig. 11). To construct SPINs, a ^1^O_2_-cleavable linker was synthesized and utilized to cage the hydroxyl group of NLG919, followed by covalent conjugation with the carboxyl group of alkynyl PEG (Mw = 2000), affording the pro-modulator PEG-NLG919 conjugate (defined as PEG-N) (Supplementary Figs. 12–15). A ^1^O_2_-responsive linker (2,2’-(propane-2,2-diylbis(sulfanediyl)diacetic acid, PSDA)-conjugated PEG chain (defined as PEG-PSDA) was synthesized according to our previous report^25^. SP7-N_3_ was then grafted with *m*PEG-alkyne (Mw = 2000), PEG-N, PEG-PSDA or alkyne-PEG-COOH at different feeding molar ratios to obtain the amphiphilic semiconducting polymer conjugates (Supplementary Figs. 16–20), which would self-assemble into nanoparticles (defined as SPIN_0_, SPIN_N_, SPN-PEG1, SPN-PEG2, and SPN-PEG3, respectively) in aqueous solution (Fig. 3a). Further modifications of SPN-PEG1, SPN-PEG2 and SPN-PEG3 on their surface with aPD-L1 afforded SPIN_A_, SPIN_D1_, and SPIN_D2_, respectively. The molar ratios of NLG919/aPD-L1 in different SPINs were shown in Fig. 3b.

A spherical morphology with uniform size distribution was observed for all SPINs (Supplementary Fig. 21). The hydrodynamic sizes of SPIN_A_, SPIN_D1_ and SPIN_D2_ (from 62.9 to 75.0 nm) were larger than those of SPIN_0_ and SPIN_N_ (~25 nm), and all SPINs showed negative zeta potentials ranging from −14.3 to −4.9 mV (Fig. 3c). They showed excellent colloidal stability in saline buffer solution as witnessed by negligible size change after 21 days of storage (Supplementary Fig. 22). SPINs showed similar absorption profiles with two characteristic peaks of SP7 at ~360 and 553 nm, and almost identical fluorescence emission peaked at ~810 nm (Supplementary Fig. 23). The NIR fluorescence of SPINs allowed for in vivo fluorescence imaging and tracking their tumor accumulation. All SPINs exhibited negligible cytotoxicity even at the high incubation concentration of 100 µg/mL (Supplementary Fig. 24). More importantly, almost no hemolysis of erythrocytes was observed after incubation with SPINs (Fig. 3d-e), testifying their suitability for systemic administration.

The ^1^O_2_ generation properties of SPINs under US treatment were almost identical to that of SPN7 prepared via nanoprecipitation (Supplementary Fig. 25). To investigate the effect of tissue penetration depth on US-induced ^1^O_2_ generation of SPINs, SPIN_D2_ solutions covered by a pork tissue were exposed under US irradiation for ESR measurement (Fig. 3f). The ESR characteristic peaks of ^1^O_2_ slightly decreased with the increasing of tissue thickness (0, 2, 4, 6, 8, and 10 cm), which was still 65% of its original at a thickness of 10 cm (Fig. 3g). This envisioned that SPINs should be able to efficiently generate ^1^O_2_ to break the ^1^O_2_-cleavable linkers to release the immunomodulators even in deep tissues.

Deep-tissue sonodynamic activation of SPINs without and with the coverage of 10-cm pork tissues was evaluated by quantifying the amounts of released immunomodulators under US treatment. Under US irradiation at a power intensity of 1.2 W/cm^2^, the release amount of aPD-L1 and NLG919 in SPIN_D2_ containing solution gradually increased as a function of treatment time regardless of the coverage of tissues (Fig. 3h). After US treatment for 30 min, the release amount of aPD-L1 and NLG919 was measured to be 76.9% and 90.6%, respectively, which was slightly decreased to 70.9% and 84.8% after coverage with 10-cm tissues. Note that the releases of aPD-L1 and NLG919 were negligible under white light irradiation when covering with 10-cm tissues (Supplementary Fig. 26). These results suggested deep-tissue activation of SPINs to control the release of immunomodulators using US, which however was impossible for white light. The PD-L1/PD-1 binding activity was only slightly inhibited for SPIN_D2_ treatment without US irradiation, while which was inhibited by 3.8-fold after US irradiation (Fig. 3i), further confirming the sonodynamic activation of SPIN_D2_.

### Activatable sono-immunotherapy of subcutaneous pancreatic tumors

The therapeutic efficacy of SPIN-mediated sono-immunotherapy was evaluated using a bilateral Panc02 tumor-bearing mouse model. In vivo NIR fluorescence imaging was used to confirm the accumulation of SPINs in tumors after administration through tail vein. The fluorescence signals of tumors gradually increased and similarly reached the maximum at 24 h post injection of SPINs, which was around 5.3–7.2-fold higher than the background signals of tumors (Supplementary Fig. 27). Moreover, intense red fluorescence signals of nanoparticles were observed in tumor tissues at 24 h post-injection of SPINs (Supplementary Fig. 28). The major accumulation of SPINs was found in tumors (15–17% ID/g) and livers (21–23% ID/g) and substantial lower accumulations were found in other organs such as the heart (<3% ID/g), spleen (<6% ID/g), lung (<4% ID/g), and kidney (<5% ID/g) (Supplementary Fig. 29). In contrast, free drug (NLG919) showed a very high accumulation percentage in liver (16% ID/g), but limited accumulation in tumors.

To evaluate ICD biomarkers after SPIN-mediated sonodynamic therapy, the expression levels of calreticulin (CRT) and high mobility group box 1 (HMGB1), and secretion of adenosine triphosphate (ATP) in tumors of mice after intravenous injection of SPINs or free-drug mixture of NLG919 and aPD-L1 at a similar dosage with or without US irradiation were investigated. Obvious fluorescence signals of singlet oxygen sensor green (SOSG) were observed in tumors of SPIN-injected and US-irradiated groups (Supplementary Fig. 30), verifying the generation of ^1^O_2_. As shown in the immunofluorescence staining images, the expression levels of CRT and HMGB1 were greatly increased after SPIN injection and US irradiation, which were at least 6.1- and 4.3-fold higher relative to those in the control groups, respectively (Supplementary Fig. 31a–c). The treatments of SPINs plus US irradiation also increased the ATP levels in tumors by more than 1.8-fold compared with the control groups (Supplementary Fig. 31d). In contrast, the levels of CRT, HMGB1 and ATP did not have remarkable changes in free-drug-injected groups regardless of US irradiation. Furthermore, the expression levels of PD-L1 in tumors after SPIN injection and US irradiation were at least 2.2-fold higher relative to that in the control group (Supplementary Fig. 32), while the treatment of SPIN_D2_ and free drug without US irradiation did not obviously change the PD-L1 expression levels. Immunohistochemical staining images revealed that the saline-injected control group had few CD3^+^ and CD8^+^ tumor-infiltrating lymphocytes inside the tumor tissues (Supplementary Fig. 33), which should be due to the intrinsically low immunogenicity of Panc02 pancreatic tumors. The numbers of CD3^+^ and CD8^+^ tumor-infiltrating lymphocytes were obviously increased after SPIN injection with US irradiation, while which were not increased much for sole SPIN_D2_ injection without US irradiation and free-drug injection regardless of US irradiation. The results indicated that SPIN-mediated treatment could effectively trigger ICD of tumor cells and thus enhance tumor immunogenicity in the tumor microenvironment.

**Fig. 4:**
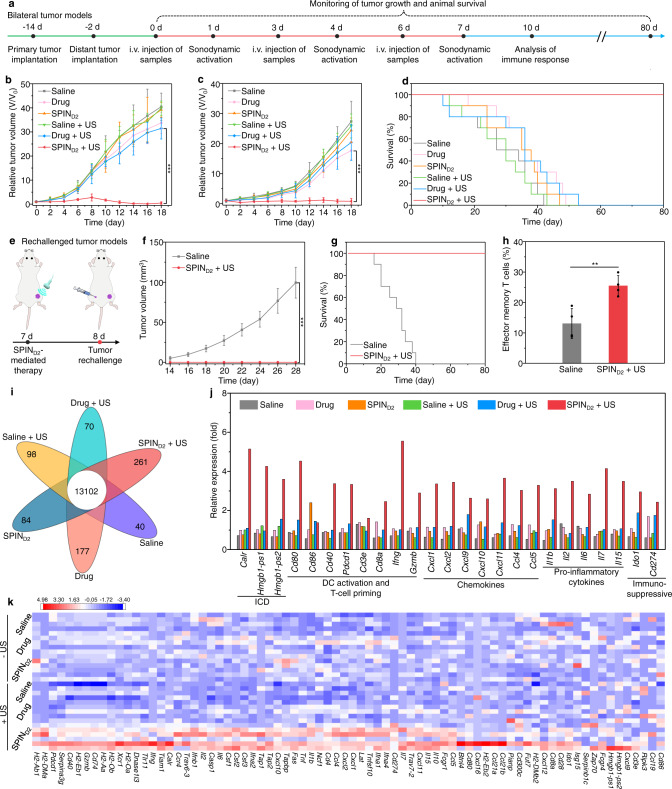
Evaluation of SPIN-mediated sono-immunotherapy in Panc02 tumor mouse model. **a** Schedule for the establishment of primary and distant tumors, triple systemic injection of SPINs (0.2 mL, 0.6 mg/mL) via tail vein, US irradiation (1.0 MHz, 1.2 W/cm^2^, 50% duty cycle, 10 min), and analysis of immune responses. **b**, **c** Relative tumor volumes of primary (**b**) and distant (**c**) tumors of Panc02 tumor-bearing C57BL/6 mice (*n* = 6) after systemic injection of saline, free-drug mixture (on day 0, 3, and 6, 4 mg/kg body weight for NLG919 and aPD-L1), or SPIN_D2_ (0.2 mL, 0.6 mg/mL) with or without US irradiation (1.0 MHz, 1.2 W/cm^2^, 50% duty cycle, 10 min). SPIN_D2_ + US versus drug + US: *P* < 0.0001 for primary tumors (**b**); SPIN_D2_ + US versus drug: *P* < 0.0001 for distant tumors (**c**). **d** Survival curves of Panc02 tumor-bearing C57BL/6 mice (*n* = 10) receiving different treatments as indicated. **e** Schematic illustration of treatment of rechallenged tumor mouse models using SPINs. **f** Growths of rechallenged tumors in Panc02 tumor-bearing mice after injection of saline or SPIN_D2_ (0.2 mL, 0.6 mg/mL) with US irradiation (1.0 MHz, 1.2 W/cm^2^, 50% duty cycle, 10 min) (*n* = 5). Saline versus SPIN_D2_ + US: *P* < 0.0001. **g** The survival curves of Panc02 tumor-bearing C57BL/6 mice after different treatments followed by tumor rechallenge (*n* = 10). **h** Flow cytometry analysis of populations of effector memory T cells in the spleen of Panc02 tumor-bearing C57BL/6 mice after different treatments followed by tumor rechallenge (*n* = 4). Saline versus SPIN_D2_ + US: *P* = 0.0059. **i** Differentially expressed gene numbers in tumor tissues of mice after different treatments. **j** Relative expression of *Carl*, *Hmgb1-ps1*, *Hmgb1-ps2*, *Cd80*, *Cd86*, *Cd40*, *Pdcd1*, *Cd3e*, *Cd8a*, *Ifng*, *Gzmb*, *Cxcl1*, *Cxcl2*, *Cxcl9*, *Cxcl10*, *Cxcl11*, *Ccl4*, *Ccl5*, *Il1b*, *Il2*, *Il6*, *Il7*, *Il15*, *Ido1*, and *Cd274* in tumors of Panc02 tumor-bearing mice after different treatments (the experiment was repeated independently five times with similar results). **k** Unsupervised hierarchical clustering of relative gene expression in tumors of Panc02 tumor-bearing C57BL/6 mice after different treatments (*n* = 5). Data are presented as mean values ± SD. Statistical significance was calculated via two-tailed Student’s *t* test; **P < 0.01, ***P < 0.001. Source data are provided as a Source Data file.

To evaluate the therapeutic efficacy of SPIN-mediated sono-immunotherapy, bilateral Panc02 tumor-bearing mice were treated through a triple therapeutic injection (on days 0, 3, and 6) and US irradiation (on days 1, 4, and 7) (Fig. 4a). Without US irradiation, no obvious cell apoptosis/necrosis was observed in the H&E and immunofluorescence terminal deoxynucleotidyl transferase (TdT) dUTP nick-end labeling (TUNEL) staining images of tumors after free drug and SPIN_D2_ injection (Supplementary Fig. 34), which was similar to that of the saline-injected control group. The growths of primary and distant tumors in the SPIN_D2_-injected group without US irradiation did not have any changes, and those in free-drug-injected group without US irradiation were slightly inhibited compared to that in the control group (Fig. 4b, c). Without US irradiation, the median survival of free drug and SPIN_D2_ injected mice was 36 and 35 days, respectively, which was slightly longer than that of the control mice (27 days) (Fig. 4d). With US irradiation, as shown in H&E and TUNEL staining images, cell apoptosis/necrosis could be observed in both primary and distant tumors of mice after injection of SPINs (Supplementary Fig. 34). Particularly the highest degree of cell apoptosis/necrosis of both primary and distant tumors was found in the SPIN_D2_ injected and US-irradiated group. The growths of primary tumors in the free drug, SPIN_0_, SPIN_N_, SPIN_A_, SPIN_D1_, and SPIN_D2_ injected group with US irradiation was inhibited by 22.3%, 35.1%, 68.9%, 76.8%, 78.6%, and 98.9% compared to that in the control group, respectively (Fig. 4b and Supplementary Fig. 35a). For distant tumors, the inhibition ratio of tumor growth in these groups was 25.6%, 30.1%, 56.9%, 68.1%, 69.5%, and 97.1%, respectively (Fig. 4c and Supplementary Fig. 35b). The median survival of SPIN_0_, SPIN_N_, SPIN_A_, and SPIN_D1_ injected and US-irradiated mice was 33, 35, 39, and 46 days, respectively (Fig. 4d and Supplementary Fig. 36). More importantly, the survival of mice was still 100% after SPIN_D2_ injection and US irradiation for 80 days. These results suggested that SPIN_D2_-mediated treatment showed the highest efficacy in inhibiting the growths of primary and distant metastatic tumors and prolonging the survival of mice.

To further confirm the superior antitumor efficacy of SPIN_D2_-mediated sono-immunotherapy, the tumor-bearing mice after treatment were rechallenged with Panc02 tumor cells on the opposite flank (Fig. 4e). Note that the volume of rechallenged tumors in the saline-injected control mice gradually increased, while no rechallenged tumors were observed for mice after SPIN_D2_ injection with US irradiation (Fig. 4f). All saline-injected control mice after tumor rechallenge died within 40 days (Fig. 4g). In contrast, the SPIN_D2_-injected and US-irradiated mice exhibited a 100% survival for 80 days even after tumor rechallenge. The population of effector memory T cells in spleen of SPIN_D2_-injected and US-irradiated mice was 25.5%, which was 1.9-fold higher than that for the control mice (Fig. 4h and Supplementary Fig. 37). This verified that SPIN_D2_-mediated sono-immunotherapy could build a long-term immunological memory to prevent tumor rechallenge.

We evaluated the gene expression profiles of tumor tissues after different treatments using multiplexed gene expression analysis. Without US irradiation, the number of differentially expressed genes was less than 180 in the saline control, free drug, and SPIN_D2_ injection groups (Fig. 4i). The expression levels of 76 immune-related genes in free-drug and SPIN_D2_-injected group did not have obvious changes compared to those in the control group (Fig. 4j, k). With US irradiation, 261 genes were found to be differentially expressed in SPIN_D2_- injected groups (Fig. 4i). The expression levels of 76 immune-related genes in the SPIN_D2_-injected and US-irradiated group were overall higher than those in the other groups (Fig. 4j, k). The upregulated genes were associated with several key immune pathways, such as MHC class II protein complex binding and lymphocyte costimulation (Supplementary Fig. 38). In the SPIN_D2_-injected and US-irradiated group, the expression levels of ICD-related genes (*Carl*, *Hmgb1-ps1*, and *Hmgb1-ps2*, >5-fold), DC activation, and T-cell priming-related genes (*Cd80*, *Cd86*, *Cd40*, *Pdcd1*, *Cd3e*, *Cd8a*, *Ifng*, and *Gzmb*, >1.2-fold), chemokine-encoded genes (*Cxcl1*, *Cxcl2*, *Cxcl9*, *Cxcl10*, *Cxcl11*, *Ccl4*, and *Ccl5*, >2.5-fold), and cytokine-encoded genes (*Il1b*, *Il2*, *Il6*, *Il7*, and *Il15*, >2.4-fold) were obviously upregulated compared to the control group (Fig. 4j). More importantly, the expression levels of immunosuppressive genes (*Ido1* and *Cd274*) were also increased by 1.6- and 4.0-fold, respectively. This further verified that SPIN-mediated US treatment could convert non-immunogenic “cold” tumor microenvironment into immunogenic “hot” one via upregulating the expression of IDO and PD-L1 in cancer cells.

SPIN_D2_-mediated antitumor immune activation effect was then investigated using flow cytometry and immunofluorescence staining. The free drug and SPIN_D2_ injection did not obviously increase the population of matured DCs (CD80^+^CD86^+^) in tumor-draining lymph nodes (TDLNs) compared to the saline control group (Supplementary Figs. 39, 40). The population of CTLs (CD3^+^CD8^+^) in primary and distant tumors of SPIN_D2_ injected mice was slightly increased compared to that in the control group, which however was 1.2-fold lower relative to that of free-drug-injected mice (Supplementary Fig. 41). Free-drug injection reduced the population of intratumor T_reg_ cells (CD25^+^Foxp3^+^) by 2.6-fold, but the treatment of SPIN_D2_ did not have a remarkable influence on the population of T_reg_ cells (Supplementary Fig. 42). The immunofluorescence staining images revealed that the expression of CD8, IFN-γ, and Granzyme B in tumor tissues of mice after free drug and SPIN_D2_ injection without US irradiation was similar or slightly higher than those in the control group (Supplementary Figs. 43, 44). The treatments of SPINs with US irradiation overall increased the populations of matured DCs in TDLNs, and CTLs in both primary and distant tumors, but reduced the populations of T_reg_ cells (Supplementary Figs. 40–42). The highest populations of matured DCs (36.4%), and CTLs in primary (24.3%) and distant tumors (23.4%) were observed for SPIN_D2_-injected and US-irradiated group, which was 2.4-, 1.6-, and 1.8-fold higher relative to that in free-drug-injected and US-irradiated group, respectively. The lowest population of intratumor T_reg_ cells in primary tumors (2.8%) and distant tumors (4.2%) was also observed for the SPIN_D2_-injected and US-irradiated group, which was 2.4- and 1.8-fold lower than that in free-drug injection plus US-irradiation group, respectively. Moreover, the expression levels of CD8, IFN-γ and Granzyme B in tumor tissues were overall increased after SPIN injection and US irradiation compared to those in the control group (Supplementary Figs. 43 and 44). Particularly, the SPIN_D2_-injected and US-irradiated group showed the highest expression levels of intratumor CD8, IFN-γ, and Granzyme B. These results verified that SPIN_D2_-mediated sono-immunotherapy triggered the highest antitumor efficacy.

### SPIN-mediated deep-tissue therapy of tumor models

**Fig. 5:**
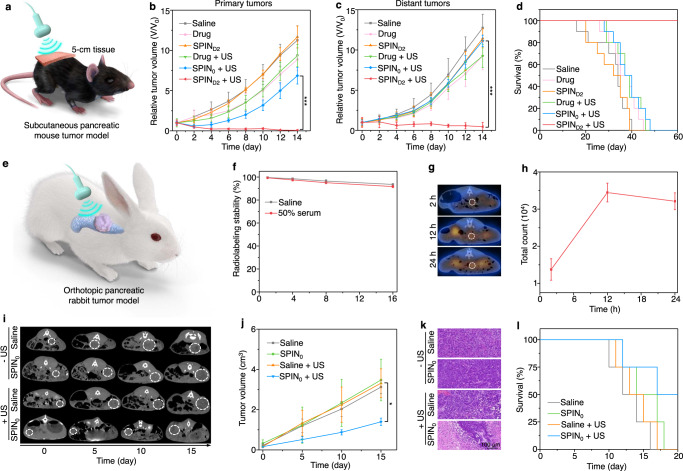
Deep-tissue therapy of tumor models using SPINs. **a** Schematic of sono-immunotherapy of subcutaneous pancreatic mouse tumors covered with 5-cm tissue. **b**, **c** Relative tumor volumes of primary (**b**) and distant (**c**) tumors of Panc02 tumor-bearing C57BL/6 mice (*n* = 5) after systemic injection of saline, free-drug mixture (on day 0, 3, and 6, 4 mg/kg body weight for NLG919 and aPD-L1), SPIN_0_ or SPIN_D2_ (0.2 mL, 0.6 mg/mL) with or without US irradiation (1.0 MHz, 1.2 W/cm^2^, 50% duty cycle, 10 min). The primary tumors were covered with 5-cm tissue under US irradiation. SPIN_D2_ + US versus SPIN_0_ + US: *P* < 0.0001 for primary tumors (**b**); SPIN_D2_ + US versus SPIN_0_ + US: *P* < 0.0001 for distant tumors (**c**). **d** Survival curves of Panc02 tumor-bearing C57BL/6 mice (*n* = 10) after different treatments for 60 days. **e** Schematic of US-mediated deep-tissue sonodynamic therapy of orthotopic pancreatic rabbit tumors. **f** Radiolabeling stability of ^131^I-SPIN_0_ after storage in saline or 50% serum at 37 °C for different time (*n* = 3). **g**, **h** SPECT imaging (**g**) and signal intensity (**h**) of orthotopic pancreatic rabbit tumors after systemic injection of ^131^I-SPIN_0_ (1.0 mL, 1.5 mg/mL) for different time (*n* = 4). The white dotted circle indicated tumors. **i** Computed tomography (CT) imaging of orthotopic pancreatic rabbit tumors after systemic injection of saline or SPIN_0_ (1.0 mL, 1.5 mg/mL) with or without US irradiation (1.0 MHz, 1.2 W/cm^2^, 50% duty cycle, 30 min). The white dotted circle indicated tumors. **j** Tumor volume of orthotopic pancreatic rabbit tumors (*n* = 3) after treatments as indicated for different days. Saline + US versus SPIN_0_ + US: *P* = 0.0108. **k** H&E staining images of orthotopic pancreatic rabbit tumors after different treatments. The experiment was repeated independently three times with similar results. **l** Survival curves of orthotopic pancreatic tumor-bearing rabbits (*n* = 4) after different treatments for 20 days. Data are presented as mean values ± SD. Statistical significance was calculated via two-tailed Student’s *t* test; *P < 0.05, ***P  <  0.001. Source data are provided as a Source Data file.

To verify the deep-tissue therapeutic efficacy mediated by SPINs, US irradiation (on days 1, 4, and 7) of tumors covered with 5-cm pork tissues was conducted after systemic injection of SPIN_D2_ (on day 0, 3, and 6) into Panc02 tumor-bearing mice (Fig. 5a). Without US irradiation, the growth of primary and distant tumors for the SPIN_D2_ injection group was similar as that of the saline control group, and free-drug treatment slightly reduced the growth of both tumors (Fig. 5b, c). The median survival of free drug and SPIN_D2_-injected mice without US irradiation was 35 and 32 days, respectively, which was almost the same as that of the control group (34 days) (Fig. 5d). Although free-drug treatment slightly increased the population of CTLs in tumors by 1.9-fold, the sole SPIN_D2_ injection did not obviously change the population of CTLs compared to saline control group (Supplementary Fig. 45). With US irradiation, the growths of both primary and distant tumors in free drug, SPIN_0_, and SPIN_D2_ injection groups were inhibited (Fig. 5b, c). In particular, the tumor growth in SPIN_D2_ injected and US-irradiated group was inhibited by 100% for primary tumors and 96.4% for distant tumors. In the SPIN_D2_ injection and US-irradiation group, the animal survival was still 100% for 60 days, while the median survival of mice in free drug and SPIN_0_ injection and US-irradiation group was only 37 days (Fig. 5d). The population of intratumoral CTLs in the SPIN_D2_-injected and US-irradiated group was 23.6%, which was 2.1- and 1.6-fold higher than that in free drug and SPIN_0_ injection with US-irradiation group, respectively (Supplementary Fig. 45). These results verified that SPIN_D2_-mediated treatment showed an excellent therapeutic efficacy for tumors covered with 5-cm tissues.

SPIN-mediated deep-tissue therapy was then evaluated using orthotopic pancreatic rabbit tumors (Fig. 5e). As the antibodies against rabbit PD-L1 have not been proven to be effective for in vivo immunotherapy, we only conducted sonodynamic therapy. To track the accumulation of SPINs in orthotopic pancreatic rabbit tumors, radionuclide ^131^I was used to label SPIN_0_ for single-photon emission computed tomography (SPECT) imaging, as iodine could be covalently immobilized onto aromatic backbones via electrophilic substitution of protons^39,40^. The obtained ^131^I-SPIN_0_ showed excellent radiolabeling stability in saline and 50% serum solutions at 37 °C (Fig. 5f). After systemic injection of ^131^I-SPIN_0_ into orthotopic pancreatic tumor-bearing rabbits, the signal intensity of tumors gradually increased and reached the maximum at 12 h post-injection time, as shown in the SPECT images (Fig. 5g, h), confirming effective accumulation of SPIN_0_ in orthotopic pancreatic rabbit tumors. The tumors of rabbits were treated via a triple therapeutic injection (on days 0, 3, and 6) and US irradiation (on days 1, 4, and 7) to evaluate the therapeutic efficacy. Without US irradiation, the growth of tumors for SPIN_0_ injected rabbits was similar as that for the saline-injected control group (Fig. 5i, j), and no apparent apoptosis/necrosis of tumor cells was observed from histological hematoxylin and eosin (H&E) and TUNEL staining images of tumor tissues (Fig. 5k and Supplementary Fig. 46). The median survival of rabbits after saline and SPIN_0_ injection without US irradiation was 12 and 14 days, respectively (Fig. 5l). With US irradiation, the tumor growth of SPIN_0_-injected rabbits was inhibited by 2.4-fold compared to the saline-injected group after treatment for 15 days (Fig. 5i, j). As shown in H&E and TUNEL staining images, obvious apoptosis/necrosis was observed for tumors in the SPIN_0_-injected and US-irradiated group (Fig. 5k and Supplementary Fig. 46). The median survival of rabbits in SPIN_0_-injected and US-irradiated group was 17 days, longer than that for the saline group (Fig. 5l). The results suggested that SPIN-mediated therapy could be used to treat orthotopic pancreatic rabbit tumors due to the deep-tissue penetration of US.

### Preliminary evaluation of minimized irAEs for sono-immunotherapy

**Fig. 6:**
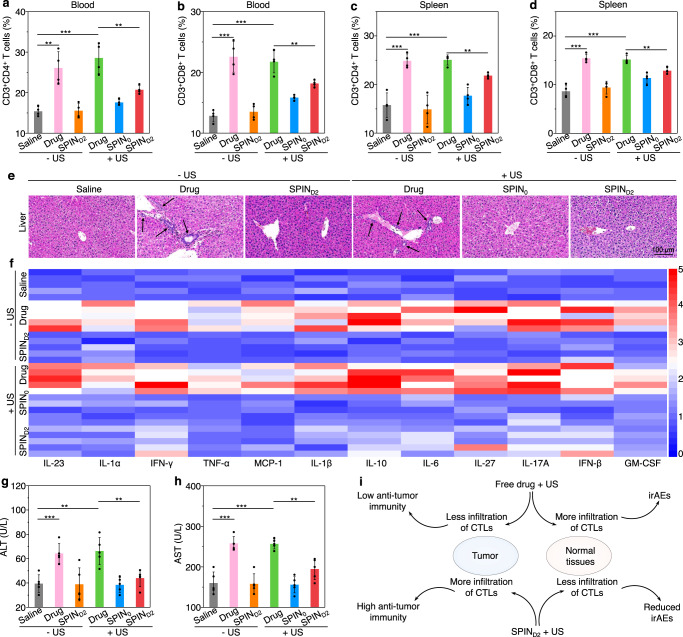
Preliminary evaluation of minimized irAEs for SPIN_D2_-mediated sono-immunotherapy. **a**, **b** Flow cytometry analysis of percentages of CD3^+^CD4^+^ Th cells (**a**), and CD3^+^CD8^+^ CTLs (**b**) in blood of mice (*n* = 4) at 30 day after systemic administrations of saline, SPIN_0_, SPIN_D2_ (0.2 mL, 1.2 mg/mL) or free-drug mixture (8 mg/kg body weight for NLG919 and aPD-L1) with or without US irradiation (1.0 MHz, 1.2 W/cm^2^, 50% duty cycle, 10 min). Saline – US versus drug − US: *P* = 0.0023; saline − US versus drug + US: *P* = 0.0006; drug + US versus SPIN_D2_ + US: *P* = 0.0071 for CD3^+^CD4^+^ Th cells (**a**); saline − US versus drug − US: *P* = 0.0004; saline − US versus drug + US: *P* = 0.0001; drug + US versus SPIN_D2_ + US: *P* = 0.0093 for CD3^+^CD8^+^ CTLs (**b**). **c**, **d** Flow cytometry analysis of percentages of CD3^+^CD4^+^ Th cells (**c**), and CD3^+^CD8^+^ CTLs (**d**) in spleen of mice (*n* = 4) after different treatments for 30 days. Saline − US versus drug − US: *P* = 0.0008; saline − US versus drug + US: *P* = 0.0005; drug + US versus SPIN_D2_ + US: *P* = 0.0015 for CD3^+^CD4^+^ Th cells (**c**); saline − US versus drug − US: *P* = 0.0001; saline  − US versus drug + US: *P* = 0.0002; drug + US versus SPIN_D2_ + US: *P* = 0.0049 for CD3^+^CD8^+^ CTLs (**d**). **e** Representative H&E staining images of liver after 30 days of treatments in different groups (white arrows indicate the infiltrated lymphocytes). The experiments were repeated independently three times with similar results. **f** Heatmap to show relative fold of cytokine levels in serum of mice after different treatments for 30 days relative to those in saline control group. **g**, **h** Serum levels of ALT (**g**) and AST (**h**) in mice (*n* = 5) after different treatments for 30 days. Saline − US versus drug − US: *P* = 0.0010; saline − US versus drug + US: *P* = 0.0020; drug + US versus SPIN_D2_ + US: *P* = 0.0054 for ALT (**g**); saline − US versus drug − US: *P* = 0.0001; saline − US versus drug + US: *P* < 0.0001; drug + US versus SPIN_D2_ + US: *P* = 0.0013 for AST (**h**). **i** Summary comparison of the antitumor immunity and irAEs between SPIN_D2_-mediated sono-immunotherapy and free-drug treatment. Data are presented as mean values ± SD. Statistical significance was calculated via two-tailed Student’s *t* test; **P < 0.01, ***P < 0.001. Source data are provided as a Source Data file.

The minimized irAEs for SPIN-mediated activatable sono-immunotherapy relative to the free-drug treatment was evaluated. Without US irradiation, the percentages of immune cells in blood and spleen for free-drug treatment were observably increased, which was 1.7-fold (CD3^+^CD4^+^ Th cells in blood), 1.8-fold (CD3^+^CD8^+^ CTLs in blood), 1.6-fold (CD3^+^CD4^+^ Th cells in spleen), and 1.8-fold (CD3^+^CD8^+^ CTLs in spleen) higher relative to those for the saline-injected control group, respectively (Fig. 6a–d and Supplementary Figs. 47, 48). In contrast, the percentages of CD3^+^CD4^+^ Th cells and CD3^+^CD8^+^ CTLs in SPIN_D2_-treated group were similar as those in the control group and were at least 1.6-fold lower than those for the free-drug treatment group, which should be attributed to the minimal pharmacokinetic property of SPIN_D2_ without sonodynamic activation. As shown in the H&E staining images, obvious accumulation of lymphocytes in the liver (especially around central vein) was observed for the free-drug treatment (Fig. 6e and Supplementary Fig. 49), while which was hardly found in the control and SPIN_D2_ treated groups. Moreover, the infiltrations of CD3^+^, CD4^+^, and CD8^+^ T cells in livers and spleens of mice after treatment of free drug were overall higher than those for the control and SPIN_D2_ treatment groups (Supplementary Figs. 50–55). The treatment of free-drug would cause severe cytokine storm as the serum levels of various cytokines including interleukin-23 (IL-23), IL-1α, interferon-γ (IFN-γ), tumor necrosis factor α (TNF-α), monocyte chemoattractant protein-1 (MCP-1), IL-1β, IL-10, IL-6, IL-27, IL-17A, IFN-β, and granulocyte–macrophage colony-stimulating factor (GM-CSF) in this group was increased by more than 2.3-fold compared to those in the control group (Fig. 6f and Supplementary Fig. 56). In contrast, the levels of these cytokines in the SPIN_D2_ treatment group were similar as those in the control group. The free-drug treatment was also found to increase the serum levels of key hepatic enzymes, including alanine aminotransferase (ALT) (by 1.6-fold), aspartate aminotransferase (AST) (by 1.6-fold), and alkaline phosphatase (ALP) (by 1.4-fold) compared to the saline control group (Fig. 6g, h and Supplementary Fig. 57a). However, the serum levels of these hepatic enzymes in the SPIN_D2_ treatment group were almost identical to those in the control group. These results suggested that free-drug treatment potentially caused hepatic irAEs, which was common for immune checkpoint inhibitor therapy^41^; while which was not observed for SPIN_D2_ treatment because of minimized bioactivities of caged NLG919 and aPD-L1.

With US irradiation, the percentages of CD3^+^CD4^+^ Th cells and CD3^+^CD8^+^ CTLs in the blood and spleen for the SPIN_D2_-injected group were more than 1.2-fold lower relative to the free-drug-treated group (Fig. 6a–d). The treatment of free drug with US treatment caused the accumulation of a larger number of lymphocytes in liver tissues, while nearly no accumulation of lymphocytes in livers was observed for SPIN_D2_ treatment with US irradiation (Fig. 6e). Moreover, the infiltration of CD3^+^, CD4^+^, and CD8^+^ T cells in the heart, liver, spleen, lung, and kidney for SPIN_D2_-treated and US-irradiated group was overall lower than those after free-drug treatment with US irradiation (Supplementary Figs. 50–55). Although obvious cytokine storm was observed for the mice after treatment with free drug and US irradiation, the serum levels of IL-23, IL-1α, IFN-γ, TNF-α, MCP-1, IL-1β, IL-10, IL-6, IL-27, IL-17A, IFN-β, and GM-CSF in SPIN_D2_-treated and US-irradiated group was overall 1.6-fold lower than those in the free-drug-treated and US-irradiated group (Fig. 6f and Supplementary Fig. 56). The serum levels of ALT, AST, and ALP in the SPIN_D2_-treated and US-irradiated group was only slightly increased compared to the control group, while they were still 1.5-, 1.3-, and 1.3-fold lower relative to those after free-drug treatment with US irradiation (Fig. 6g, h and Supplementary Fig. 57a). Furthermore, in the SPIN_D2_-treated and US-irradiated group, the serum levels of creatinine (CRE), urea, and gamma-glutamyl transferase (GGT) (Supplementary Fig. 57b–d), several vital blood parameters (Supplementary Fig. 58), the body weight of mice (Supplementary Fig. 59), and histological morphologies of major organs (heart, liver, spleen, lung, and kidney) (Fig. 6e and Supplementary Fig. 49) did not change obviously. These results demonstrated that SPIN_D2_ treatment and US irradiation had minimal impact on disrupting homeostatic immune tolerance in normal organs of mice, indicating the reduced probability of irAEs relative to free-drug treatment regardless of US irradiation (Fig. 6i).

## Discussion

With deep-tissue penetration and safe nonionizing radiation, US serves as an ideal external stimulus to precisely activate cancer immunotherapy in situ in living subjects. Although sonodynamic process has been combined with chemotherapy, chemodynamic, or phototherapy to treat cancer in preclinical settings, it has been rarely exploited for immunotherapy with only several examples wherein nanosonosensitizers and immune checkpoint inhibitors were independently administrated into mice (Supplementary Table 1)^42,43^. To acquire the specificity of US-induced immune activation, we have synthesized semiconducting polymer immunomodulatory nanoparticles (SPINs) that underwent sonodynamic process to generate ^1^O_2_ to specifically cleave the ^1^O_2_-sensitive linkers and release the covalently linked immunomodulators. Such a sonodynamic activation mechanism is distinct from the conventional US-mediated cavitation used in drug release from nanoparticles or microbubbles^44,45^, wherein the physically loaded drug molecules still encounter the issue of nonspecific release in normal tissues.

Although SPNs have been used for optical imaging and phototherapy^46–49^, their sonodynamic properties have been unknown. Screening of SPNs led to the discovery of a robust sonosensitizer (SPN7), which had the sonodynamic ability to produced ^1^O_2_ at least 3.0-fold stronger than other commonly used sonosensitizers including small molecule dyes and TiO_2_ at the same mass concentration (Fig. 2f). More importantly, SPNs intrinsically possessed excellent sonodynamic stability (Fig. 2g), which was superior to small molecule sonosensitizers that are structurally fragile to US irradiation (Fig. 2h and Supplementary Fig. 6). Besides, the sonodynamic ^1^O_2_ generation ability of SPNs was retained even in the tissue depth of 10 cm (Fig. 3g), forming the basis of deep-tissue activatable sono-immunotherapy. This was also validated by sole sonodynamic therapy using SPIN_0_, which inhibited the growth of orthotopic pancreatic rabbit tumors by 2.4-fold (Fig. 5j, k).

Molecular reengineering of SPN7 allowed us to covalently conjugate immunomodulators (NLG919 and aPD-L1) to the backbone via a ^1^O_2_-cleavable linker to form pro-therapeutic systems (SPINs) wherein the bioactivities of immunomodulators are intrinsically silenced and only triggered upon US irradiation (Fig. 3a). Although pancreatic tumors have excessive extracellular matrix (ECM) proteins, SPINs passively accumulated in subcutaneous pancreatic mouse tumors after systematic administration (Supplementary Fig. 27), which should be due to effective enhanced permeability and retention (EPR) effect^50,51^. Under US irradiation, SPINs were able to efficiently generate ^1^O_2_ to ablate tumor cells and induce ICD (Supplementary Fig. 31), which led to the enhanced tumor infiltration of lymphocytes (Supplementary Fig. 33) and thus primed tumors for immunotherapy. Meanwhile, the generation of ^1^O_2_ triggered in situ release of immunomodulators from SPINs in the tumor microenvironment, remotely activating localized immunotherapy. Among five SPINs, SPIN_D2_ had the highest sono-immunotherapeutic efficacy in inhibiting growth of primary and distant tumors, extending animal survival and preventing tumor recurrence (Fig. 4b–h). The therapeutic benefits were also proven to be obtainable for mouse tumors covered with 5-cm pork tissues, verifying the in vivo deep-tissue activatable sono-immunotherapy (Fig. 5b–d).

The underlying molecular mechanism study via multiplexed gene expression analysis showed that SPIN_D2_ injection and US irradiation upregulated the gene expression level of *Ido1* and *Cd274* in tumors by 1.6- and 4.0-fold, respectively (Fig. 4j, k). To date, photothermal and photodynamic effects induced upregulated expressions of IDO and PD-L1 in tumors cells has been reported^52–54^, while the influence of sonodynamic effect on these immunosuppressive proteins has not been evaluated. This study disclosed that SPIN-mediated US treatment could reprogram tumor microenvironment to transform the non-immunogenic “cold” tumors into immunogenic “hot” ones via upregulating the expression of IDO and PD-L1. Therefore, targeting of the IDO and PD-L1 pathways followed by US treatment should be an effective strategy to strengthen the immune response. Note that even though the injected dosage of NLG919 and aPD-L1 was overall lower than those used for combinational immunotherapy in previous studies (Supplementary Table 2), SPIN_D2_ almost completely eradicated both primary and distant tumors and fully inhibited rechallenged tumors. Such a high antitumor immune response elicited by SPIN_D2_ should be attributed to the co-existence of two immunomodulators (NLG919 for IDO inhibition and aPD-L1 for PD-L1 blocking) which synergistically improved the tumoricidal activity of CTLs (Supplementary Figs. 41–44).

The high specificity of sonodynamic activation mechanism of SPINs enabled precise restriction of immunotherapeutic action to the tumors of living mice, potentially reducing the incidence of irAEs. This was supported by multi-fold observations. Administration of SPIN_D2_ alone without US irradiation even had poor antitumor efficacy than free-drug administration and did not significantly affect the populations of immune cells in normal organs and serum cytokine levels as free drug did (Fig. 6a–f). In contrast, directed US irradiation after SPIN_D2_ accumulation in tumors led to at least twofold increase in the population of CTLs in the tumor tissues as compared with free-drug administration; whereas, the populations of effector T cells were only slightly higher than those in the saline group and 1.2-fold lower than free-drug administration in normal organs (Fig. 6a–d). This could be explained by the minimal bioactivity of NLG919 and aPD-L1 within SPIN_D2_ without US irradiation.

In summary, we have developed deep-tissue activatable sono-immunotherapeutic nanoagents based on SPNs and validated their efficacy in the pancreatic tumor mouse model. SPIN-mediated sono-immunotherapy capitalizes on excellent sonodynamic properties of SPNs to noninvasively eradiate tumors and enhance the immunogenicity of pancreatic tumors via induction of ICD, and also remotely activate the therapeutic functions via scission of covalently conjugated immunomodulators in tumor in situ. Deep-tissue therapy was achieved using SPINs on subcutaneous pancreatic mouse tumors covered with 5-cm tissues and rabbit orthotopic pancreatic tumors. Thus, SPINs represent a type of precision immunotherapeutic agents which elicit high antitumor immunity without significantly breaking systemic immune tolerance. Furthermore, the flexibility in structural modification of SPINs allows to easily generalize sonodynamic activation approach beyond checkpoint blockade immunotherapy through conjugation other immunotherapeutic molecules. Thus, our study provides a precision nanoplatform to further equip nano-immunotherapy with temporospatial control over immunomodulation.

## Methods

### Materials

All chemicals were purchased from Sigma-Aldrich (St. Louis, MO, USA) unless otherwise declared. SPs were purchased from Luminescence Technology Corp. (Xizhi, Taiwan). NLG919 and aPD-L1 were purchased from MedChemExpress (Monmouth Junction, NJ, USA) and Bioxcell (West Lebanon, NH, USA), respectively. Mouse PD-1[biotinylated]: PD-L1 inhibitor screening assay kit was purchased from BPS Bioscience (San Diego, CA, USA). The chemiluminescence ATP determination kit was purchased from Thermo Fisher Scientific (Waltham, MA, USA). All other antibodies were purchased from Biolegend, (San Diego, CA, USA) or Abcam Inc. (Cambridge, MA, USA).

### Characterization

TEM images were recorded using a JEM 1400 transmission electron microscope. The microscope was operated at 120 kV. The nanoparticle diameter and zeta potential measurements were conducted on a Malvern Nano-ZS Particle Sizer. GPC curve was obtained using a Shimazu LC-VP system (standard: styrene; eluent: tetrahydrofuran). UV–Vis spectra were recorded on an UV-2450 spectrophotometer using UV Probe software (version 2.3). Fluorescence spectra were recorded on a Fluorolog 3-TCSPC spectrofluorometer using fluorolog software (version 3.2). NMR spectra were collected on a Bruker Avance II NMR system (300 MHz). ESR spectra were recorded on an electron spin resonance paramagnetic wave spectrometer (JEOL-FA200). HPLC analyses were performed on an Agilent 1260 system equipped with a G1311B pump, a UV detector and an Agilent Zorbax SB-C18 RP (9.4 × 250 mm) column. Confocal fluorescence imaging of tissue sections was performed using a LSM800 confocal laser scanning microscope (Carl Zeiss, Germany). Flow cytometry analysis of immune cells was performed using a Fortessa X20 flow cytometer (BD Biosciences, USA). In vivo animal fluorescence images were captured using an IVIS imaging system (IVIS-CT machine, PerkinElmer). In vivo SPECT images of tumor-bearing rabbits were captured using a NanoSPECT/CT animal imaging system (Bioscan Ltd., Washington, DC) with a tube voltage of 80 kV, tube current of 450 μA, and slice thickness of 45 μm.

### Preparation of SPNs

All SPNs were similarly synthesized via a nanocoprecipitation. Taking SPN7 as an example, SP7 (0.25 mg) and PEG-b-PPG-b-PEG (20 mg) were dissolved in THF (1 mL), followed by rapid injection into the mixture of Milli-Q water and THF (V/V = 9/1) (10 mL) under sonication for 5 min. After evaporation of THF under a gentle nitrogen flow, the solution was filtered through a Millipore poly(ether sulfone) syringe-driven filter (0.22 μm) and then washed with Milli-Q water using Millipore ultrafiltration devices (50 K) under centrifugation (3170×*g*, 25 min). The obtained samples were stored in the dark at 4 °C.

### Synthesis of compound 1

Benzo[1,2-b:4,5-b’]dithiophene-4,8-dione (0.50 g, 2.26 mmol), zinc powder (0.35 g, 5.38 mmol) and sodium hydride (1.36 g, 34.0 mmol) were dissolved in water. The mixture was stirred at 100 °C for 2 h. To the above solution, 1,6-dibromohexane (3.5 mL) and tetrabutylammonium bromide (0.15 g, 0.45 mmol) were added. The reaction was carried out at 100 °C for 18 h. The crude product was extracted with dichloromethane. Purification of the residue by using silica gel column chromatography (hexane/dichloromethane = 5/1) gave compound 1 (0.89 g, 59.3% yield) as solid. ^1^H NMR (300 MHz, CDCl_3_): δ = 7.47 (d, J = 5.5, 2H), 7.38 (d, J = 5.5, 2H), 4.29 (t, J = 6.4, 4H), 3.52–3.41 (m, 4H), 2.00–1.83 (m, 8H), 1.71–1.57 (m, 8H).

### Synthesis of compound 2

Compound 1 (0.24 g, 0.44 mmol) was dissolved in dichloromethane (2 mL). Bromine (60 μL) was dropwise added and stirred at room temperature for 6 h. The solution was washed with sodium thiosulfate and extracted with dichloromethane. Purification of the residue by using silica gel column chromatography (hexane/dichloromethane, 10/1) gave compound 2 (162 mg, 52.3%) as grange solid. ^1^H NMR (400 MHz, CDCl_3_): δ = 7.43 (d, J = 2.0, 2H), 4.27–4.18 (m, 4H), 3.54–3.32 (m, 4H), 1.97 (dd, J = 14.0, 6.9, 4H), 1.87 (dd, J = 14.0, 6.7, 4H), 1.69–1.49 (m, 8H).

### Synthesis of SP7-N_3_

Compound 2 (36.4 mg, 0.051 mmol), 4,7-bis(4,4,5,5-tetramethyl-1,3,2-dioxaborolan-2-yl)benzo[c][1,2,5]thiadiazole (20 mg, 0.051 mmol), tetrakis(triphenylphosphine)palladium(0) [Pd(PPh_3_)_4_] (3.0 mg, 0.0026 mmol) and potassium carbonate were added in a 50 mL Schlenk tube. The system was degassed via freeze-pump-thaw cycles three times. Then methyltrioctylammonium chloride (2.0 mg) in water/toluene solution was added and degassed via freeze-pump-thaw cycles for one time. The mixture was stirred at 100 °C. After 24 h, the solvent was concentrated under a vacuum. The crude product was precipitated into methanol to yield SP7-Br (21.7 mg, 37.9%) as purple solid. Then SP7-Br (21.7 mg) and sodium azide (10.9 mg, 0.17 mmol) were dissolved in dimethylformamide/tetrahydrofuran. The mixture was stirred at room temperature for 12 h and then concentrated under vacuum. The crude product was washed with water and extracted with dichloromethane to obtain SP7-N_3_ (17.9 mg, 97.8%) as purple solid. ^1^H NMR (300 MHz, CDCl_3_): δ = 8.00–7.13 (m, 4H), 4.36–4.02 (m, 4H), 2.85 (d, J = 21.5, 4H), 2.31–1.95 (m, 8H), 1.62 (d, J = 6.4, 8H).

### Synthesis of 2,2’-(propane-2,2-diylbis(sulfanediyl))diethanol (PSDE)

To a solution of acetic anhydride (5 mL) with 2-mercaptoethanol (4 g, 51.2 mmol), trichlorotitanium(IV) trifluoromethanesulfonate [TiCl_3_(OTf)] (320 mg, 0.53 mmol) was added. The mixture was stirred at room temperature for 3 h. Purification of the residue by using silica gel column chromatography gave 2-mercaptoethyl acetate. ^1^H NMR (300 MHz, CDCl_3_): δ = 4.33 (t, 4H), 2.93 (t, 4H), 2.08 (s, 6H).

2-Mercaptoethyl acetate (4.0 g, 33.20 mmol) and trifluoroacetic acid (150 μL) were dissolved in acetone (880.2 mg, 15.1 mmol). The mixture was stirred at room temperature for 24 h. Purification of the crude product by using silica gel column chromatography (hexane/ethyl acetate = 1/1) gave 2,2’-propane-2,2-diylbis(sulfanediyl)) bis(ethane-2,1-diyl) diacetate (PSDE-acetyl) (4.34 g, 85.5%) as colorless liquid. ^1^H NMR (300 MHz, CDCl_3_): δ = 4.01 (t, J = 6.9, 4H), 2.67 (t, J = 6.9, 4H), 1.91–1.84 (m, 6H), 1.40 (s, 6H).

PSDE-acetyl (1.42 g, 5.06 mmol) and potassium hydroxide (1.2 g, 23.5 mmol) were dissolved in methanol (10 mL). The mixture was stirred at room temperature for 16 h and then concentrated under a vacuum. The residues were poured into water, hydrochloric acid solution (1 M) was dropwise added and then extracted by ethyl acetate. The organic layer was concentrated under a vacuum to give PSDE (0.83 g, 96.6%) as yellow liquid. ^1^H NMR (300 MHz, CDCl_3_): δ = 3.81 (t, J = 6.0, 4H), 2.89 (t, J = 6.0, 4H), 1.65 (s, 6H).

### Synthesis of PEG-N

NLG919 (40 mg, 0.14 mmol), bis(trichloromethyl) carbonate (420 mg, 1.4 mmol) and triethylamine (60 μL) were dissolved in anhydrous dichloromethane (5 mL) at 0 °C. The mixture was stirred at room temperature for 2 h. PSDE (210 mg, 1.12 mmol) and 4-(dimethylamino)pyridine (86.6 mg, 0.71 mmol) were added to the above solution and the mixture was stirred at room temperature for 10 h and then concentrated under vacuum. Purification of the crude product by using silica gel column chromatography (hexane/ethyl acetate = 2/1) gave PSDE-NLG919 (21.3 mg, 33.5%) as yellow solid. ^1^H NMR (300 MHz, CDCl_3_): δ = 7.82–6.59 (m, 6H), 4.15 (t, J = 6.9, 1H), 3.69 (t, J = 6.1, 1H), 2.87–2.70 (m, 4H), 2.50 (dd, J = 14.3, 6.7, 4H), 2.04 (s, 2H), 1.98 (s, 1H), 1.54 (s, 6H), 1.28–1.14 (m, 10H).

Alkyne-PEG-COOH (78 mg, 0.037 mmol), 1-ethyl-3-(3-dimethylaminopropyl)carbodiimide (14 mg, 0.074 mmol) and 4-dimethylaminopyridine (5 mg, 0.037 mmol) were dissolved in dichloromethane (2 mL) for 1 h. Then PSDE-NLG919 (20 mg, 0.037 mmol) was added to the above solution. The mixture was stirred at room temperature for 12 h. The solvent was concentrated under a vacuum and the residue was purified via dialysis in water. After lyophilization, PEG-N was obtained as white powder (52.1 mg, 53.2%). ^1^H NMR (300 MHz, CDCl_3_): δ = 7.8–7.28 (m, 6H), 4.44–4.03 (m, 12H), 3.90–3.82 (m, 2H), 3.81–3.26 (m, 180H), 3.45–3.34 (m, 4H), 2.94–2.71 (m,2H), 2.43 (s, 0H), 2.29 (m, 1H), 2.03 (d, J = 9.7, 1H), 1.60 (s, 6H), 1.37–1.04 (m, 10H).

### Synthesis of SPIN_0_

SP7-N_3_ (6.86 mg), *m*PEG (40 mg, 0.02 mmol), copper(I) bromide (7.46 mg) and N,N,N’,N”,N”-pentamethyldiethylenetriamin (60 μL) were dissolved in tetrahydrofuran (6 mL). The mixture was stirred at room temperature for 24 h. The solvent was removed by a rotary evaporator. The residue was purified by dialysis followed by lyophilization to obtain SPIN_0_ as purple solid. ^1^H NMR (300 MHz, CDCl_3_): δ = 7.78–7.03 (m, 4H), 4.45 (s, 1H), 4.65–4.15 (m, 1H), 3.98–3.29 (m, 180H), 1.79 (d, J = 132.7, 2H), 1.26 (s, 10H), 0.83 (s, 2H).

### Synthesis of SPIN_N_

SP7-N_3_ (6.86 mg), PEG-N (52 mg, 0.02 mmol), CuSO_4_ (9.8 mg, 0.04 mmol), and sodium ascorbate (18 mg, 0.08 mmol) were dissolved in dimethylformamide/tetrahydrofuran (2 mL/4 mL). The mixture was stirred at room temperature for 24 h. The solvent was removed by rotary evaporator. The residue was purified by dialysis followed by lyophilization to obtain SPIN_N_ as purple solid. ^1^H NMR (400 MHz, CDCl_3_): δ = 8.22–7.38 (m, 4H), 4.73 (s, 1H), 4.41 (d, J = 41.8, 2H), 4.25–4.17 (m,3H), 3.98–3.29 (m, 180H), 2.96 (d, J = 18.5, 4H), 1.96–1.84 (m, 2H), 1.66 (s, 6H), 1.45–1.07 (m, 10H), 0.97–0.76 (m, 2H).

### Synthesis of SPIN_A_

SP7-N_3_ (6.86 mg), PEG-PSDA (9 mg, 0.004 mmol), *m*PEG (32 mg, 0.016 mmol), CuSO_4_ (9.8 mg, 0.04 mmol), and sodium ascorbate (18 mg, 0.08 mmol) were dissolved in dimethylformamide/tetrahydrofuran (2 mL/4 mL). The mixture was stirred at room temperature for 24 h. The solvent was removed by a rotary evaporator. The residue was purified by dialysis followed by lyophilization to obtain SPN-PEG1 as purple solid.

SPN-PEG1 (1 mg) was mixed with 20 mg EDC and 12 mg NHS in 5 mL PBS buffer under stirring at room temperature for 3 h to activate the carboxyl groups of PSDA. Then, 2 mL PBS solution of aPD-L1 (2 mg/mL) was added to above solution, and the mixed solution was stirred at 4 °C for 4 h. The products were purified by washing with PBS three times using 300 K centrifugal filter units (Millipore) under centrifugation (3170×*g*, 10 min for each time) to obtain SPIN_A_.

### Synthesis of SPIN_D1_

SP7-N_3_ (6.86 mg), PEG-N (41.6 mg, 0.0016 mmol), PEG-COOH (8 mg, 0.004 mmol), CuSO_4_ (9.8 mg, 0.04 mmol), and sodium ascorbate (18 mg, 0.08 mmol) were dissolved in dimethylformamide/tetrahydrofuran (2 mL/4 mL). The mixture was stirred at room temperature for 24 h. The solvent was removed by a rotary evaporator. The residue was purified by dialysis followed by lyophilization to obtain SPN-PEG2 as purple solid.

The carboxyl groups of SPN-PEG2 were activated by EDC/NHS in PBS buffer, and then conjugated to aPD-L1 according to the above-mentioned protocol. The samples were purified by washing with PBS for three times using centrifugal filter units under centrifugation to obtain SPIN_D1_.

### Synthesis of SPIN_D2_

SP7-N_3_ (6.86 mg), PEG-N (41.6 mg, 0.016 mmol), PEG-PSDA (9 mg, 0.004 mmol), CuSO_4_ (9.8 mg, 0.04 mmol), and sodium ascorbate (18 mg, 0.08 mmol) were dissolved in dimethylformamide/tetrahydrofuran (2 mL/4 mL). The mixture was stirred at room temperature for 24 h. The solvent was removed by a rotary evaporator. The residue was purified by dialysis followed by lyophilization to obtain SPN-PEG3 as purple solid.

The carboxyl groups of SPN-PEG3 were activated by EDC/NHS in PBS buffer, and then conjugated to aPD-L1 according to the above-mentioned protocol. The samples were purified by washing with PBS for three times using centrifugal filter units under centrifugation to obtain SPIN_D2_.

### Measurement of ^1^O_2_ generation

SPNs were dissolved in 1× PBS (pH = 7.4) and small molecules were dissolved in 1× PBS containing 10% methanol or tetrahydrofuran. Sample solutions (0.1 mL, 20 μg/mL) were mixed with TEMP (10 μL, Dojindo Molecular Technologies), followed by US irradiation (1.0 MHz, 1.2 W/cm^2^, 50% duty cycle) for 5 min. Immediately after treatment, ESR spectra of the samples were recorded on a ESR spectrometer. The generation of ^1^O_2_ was quantified by measuring the amounts of free electrons and normalized to the mass of samples. For stability studies, the samples (0.1 mL, 20 μg/mL) were exposed under US irradiation (1.0 MHz, 1.2 W/cm^2^, 50% duty cycle) for different cycles (5 min for each cycle). After each cycle, ESR spectra were acquired to quantify the generation of ^1^O_2_. For photodynamic ^1^O_2_ generation, the sample solutions (1 mL, 20 μg/mL) were mixed with SOSG agent (2 μL, 500 μM, Molecular Probes Lnc.). The solution was irradiated with white light (0.1 W/cm^2^) for 1 min. The fluorescence intensity of SOSG at 528 nm before (F_0_) and after light irradiation (F) was measured using a spectrofluorometer.

### Evaluation of tissue penetration depth

Commercial porcine muscle tissues were purchased from local supermarkets (Shanghai, China), and all experiments involving porcine muscle tissues were approved by the Institutional Animal Care and Use Committee at Tongji University. SPIN_D2_ solutions (0.1 mL, 20 μg/mL) in 1× PBS (pH = 7.4) were placed in 2-mL tubes and mixed with TEMP (10 μL). The tubes were covered with porcine muscle tissues with different thicknesses (0, 2, 4, 6, 8, and 10 cm) and then irradiated by US (1.0 MHz, 1.2 W/cm^2^, 50% duty cycle) for 3 min, followed by ESR measurement of ^1^O_2_ generation.

### Deep-tissue sonodynamic activation of SPINs

SPIN_D2_ solutions (0.5 mL, 40 μg/mL) in 1× PBS (pH = 7.4) were placed in 2-mL tubes. The tubes were covered with pork tissues at the thickness of 10 cm, followed by US irradiation (1.0 MHz, 1.2 W/cm^2^, 50% duty cycle) or white light irradiation (0.1 W/cm^2^) for different times (10, 20, and 30 min). The release of NLG919 and aPD-L1 was measured using HPLC and Bradford protein assay, respectively.

### PD-L1/PD-1 binding activity assay

Free aPD-L1 or SPIN_D2_ solutions (0.1 mL, 40 µg/mL) in assay buffer (pH = 7.4) were exposed under US irradiation (1.0 MHz, 1.2 W/cm^2^, 50% duty cycle) for 20 min. PD-L1/PD-1 binding activity was investigated using mouse PD-1[biotinylated]: PD-L1 inhibitor screening assay kit according to the manufacturer’s protocol.

### Cytotoxicity assay

Panc02 cancer cells were purchased from the National Infrastructure of Cell Line Resource (NICR). The cells were cultured in DMEM cell culture medium containing 10% fetal bovine serum, 100 U/mL penicillin, and 100 μg/mL streptomycin in an incubator at 37 °C and 5% CO_2_. The cells were seeded in a 96-well cell culture plate at a density of 1 × 10^4^ cells/well, followed by incubation with SPINs at the final concentrations of 0, 20, 40, 60, 80, or 100 μg/mL for 24 h. After treatments, the cell viability was evaluated using CCK-8 assay.

### Establishment of pancreatic tumor models

Animal experiments were performed according to the protocols authorized by the Institutional Animal Care and Use Committee at Tongji University. The maximum tumor size was 2.0 cm in mice and 5.0 cm in rabbits permitted by the ethics committee. All the tumor sizes were not exceeded the limits in our experiments. Female healthy C57BL/6 mice (4–6 weeks) were purchased from Shanghai Slac Laboratory Animal Center. The mice were housed with a 12 h/12 h light/dark cycle (temperature: 20–25 °C, humidity: 50–65%) and fed with food and water ad libitum. To establish subcutaneous pancreatic tumor model, 2 × 10^6^ Panc02 cancer cells suspended in 100 μL 1× PBS were subcutaneously injected into the right flank of C57BL/6 mouse. To establish bilateral pancreatic tumor models, 2 × 10^6^ Panc02 cancer cells suspended in 100 μL 1× PBS were subcutaneously injected into the right flank of each mouse as the primary tumors. After 12 days, 2 × 10^6^ Panc02 cells were subcutaneously injected into the left flank of mouse as the distant tumors.

### Hemolysis assay

Fresh blood was collected from C57BL/6 mice and stored in an ice box to prevent clotting. The blood was centrifuged (15,000×*g*, 10 min) to remove supernatant, followed by purification (5 times) via successive rinsing with 1× PBS buffer to obtain red blood cells. The red blood cells were treated with 1× PBS (negative control), 1% Triton X-100 (positive control), or SPINs at different final concentrations (20, 40, 60, 80, and 100 μg/mL) at room temperature for 2 h. After centrifugation (15,000×*g*, 1 min), the absorbance of hemoglobin at 398 nm in supernatants was measured using a SpectraMax M5 microplate reader (Molecular Devices, Sunnyvale CA, USA). The hemolysis percentages were calculated by dividing the absorbance difference between the samples and the negative control by the absorbance difference between the positive and negative controls.

### Tumor accumulation evaluation

The subcutaneous pancreatic tumor-bearing C57BL/6 mice were randomly divided into five groups (*n* = 3) and in vivo NIR fluorescence imaging was performed to evaluate the tumor accumulation of SPINs. PBS solutions of SPINs (0.2 mL, 0.6 mg/mL) were systematically injected into mice in each group via tail vein. At *t* = 0, 6, 12, 24, 36, and 48 h post-injection, fluorescence images of mice were acquired using the IVIS spectrum imaging system with excitation at 660 nm and emission at 710 nm. The obtained images were analyzed using the Living Image 4.3 Software to measure the fluorescence intensities of tumors. At 24 h post-injection timepoint, the mice were euthanized to extract tumors. The collected tumor tissues were cut into 10-μm sections after fixation (4% paraformaldehyde) and dehydration (30% sucrose solution). After further staining the cell nucleus with DAPI, the tumor sections were observed using a confocal laser scanning microscope (LSM800, Carl Zeiss, Germany) to evaluate the accumulation of SPINs. The confocal fluorescence microscopy images were analyzed using Zen Blue software (version 2.3) (Carl Zeiss).

### In vivo biodistribution studies

The subcutaneous pancreatic tumor-bearing C57BL/6 mice were randomly divided into five groups (*n* = 5) and systematically injected with PBS solution of SPINs (0.2 mL, 0.6 mg/mL) or free NLG919 (8 mg/kg body weight) via tail vein. The mice were euthanized at *t* = 24 h post-injection to extract the tumor, heart, liver, spleen, lung and kidney. The organs were weighted and homogenized in PBS solution (1 mL). After centrifugation (3170×*g*, 20 min) of homogenized solutions, the fluorescence intensity of supernatant for each sample was measured using a fluorescence spectrophotometer. HPLC was used to measure the content of NLG919. The %ID/g was calculated by dividing the percentages of injected dosing by the mass of organs.

### Detection of intratumor ^1^O_2_ generation

Subcutaneous pancreatic tumor-bearing C57BL/6 mice were systematically injected with 0.2 mL saline, PBS solution of SPINs (0.6 mg/mL) or free-drug mixture (4 mg/kg body weight for NLG919 and aPD-L1) via tail vein. At 24 h post-injection timepoint, 20 μL SOSG probe (200 μM in 1× PBS buffer containing 10% methanol) was locally injected into tumors of each mouse. The tumors were then exposed under US irradiation (1.0 MHz, 1.2 W/cm^2^, 50% duty cycle) for 10 min. Subsequently, the mice were euthanized to extract tumor tissues. The collected tumors were fixed with paraformaldehyde (4%), dehydrated in sucrose solution (30%), embedded, and cryosectioned into 10-µm sections. The tumor sections were stained with DAPI and then observed under a confocal laser scanning microscope (LSM800, Carl Zeiss, Germany).

### In vivo ICD evaluation

The subcutaneous pancreatic tumor-bearing C57BL/6 mice were randomly divided into ten groups. The mice in each group were systematically injected with 0.2 mL saline, PBS solution of SPINs (0.6 mg/mL), or free-drug mixture (4 mg/kg body weight for NLG919 and aPD-L1) via tail vein. For US treatment groups, US irradiation (1.0 MHz, 1.2 W/cm^2^, 50% duty cycle, 10 min) of tumors was conducted at *t* = 24 h post-injection. After treatments for 24 h, the mice were euthanized to extract tumors. The intertumoral ATP levels (*n* = 4) were detected using a chemiluminescence ATP determination kit. For immunofluorescence staining of CRT and HMGB1, the tumors were fixed with paraformaldehyde (4%), dehydrated in sucrose solution (30%), embedded, and then cut into 10-μm sections. After drying at room temperature, the sections were immersed in 1× PBS solution containing Triton X-100 (0.1%) for 30 min, and then incubated with bovine serum albumin solution (2.5%) for 70 min. These sections were then stained with anti-CRT antibody (dilution 1:200, Abcam Inc., cat. no. ab227444) or anti-HMGB1 antibody (dilution 1:200, Abcam Inc., cat. no. ab79823) at 4 °C for 12 h. After washing with 1× PBS solution for 3 times to remove unbound antibody, the sections were stained with Alexa Fluor 488-conjugated goat anti-rabbit second antibody (dilution 1:200, Abcam Inc., cat. no. ab150077) at room temperature for 2 h, followed by staining of cell nucleus with DAPI (dilution 1:1000) at room temperature for 30 min. Fluorescence images of stained sections were captured using a confocal laser scanning microscope (LSM800, Carl Zeiss, Germany).

### Evaluation of PD-L1 expression levels

The subcutaneous pancreatic tumor-bearing C57BL/6 mice were treated as above described. After treatments for 72 h, the mice were euthanized to extract tumors and the collected tumors were cut into 10-μm sections for immunofluorescence staining using anti-PD-L1 antibody (dilution 1:100, Abcam Inc., cat. no. ab213480) and Alexa Fluor 488-conjugated goat anti-rabbit second antibody (dilution 1:200, Abcam Inc., cat. no. ab150077).

### Immunohistochemical staining

The subcutaneous pancreatic tumor-bearing C57BL/6 mice were systematically injected with 0.2 mL saline, PBS solution of SPINs (0.6 mg/mL) or free-drug mixture (4 mg/kg body weight for NLG919 and aPD-L1) via tail vein. For US treatment groups, US irradiation (1.0 MHz, 1.2 W/cm^2^, 50% duty cycle, 10 min) of tumors was conducted at *t* = 24 h post-injection. The mice were euthanized to extract tumors after 3 days of treatments. The collected tumors were fixed with paraformaldehyde (4%), dehydrated in a series of ethanol solution, embedded in paraffin, and cut into 7-μm sections. The sections were deparaffinized, and successively incubated with proteinase K at room temperature for 10 min, peroxidase blocking solution for 10 min, and BSA solution (3%) for 50 min. The sections were then incubated with anti-CD3 antibody (dilution 1:100, Abcam Inc., cat. no. ab16669) or anti-CD8α antibody (dilution 1:100, Abcam Inc., cat. no. ab ab217344) at 4 °C for 12 h, followed by incubation with horseradish peroxidase (HRP)-conjugated secondary antibody (dilution 1:200, Abcam Inc., cat. no. ab205718) at room temperature for 1 h. After washing with PBS, the sections were incubated with 3,3’-diaminobenzidine substrate—chromogen for 10 min to visualize the color. Then, the sections were washed with water, counterstained with Harris hematoxylin at room temperature for 2 min, dehydrated with ethanol, and then mounted.

### In vivo antitumor efficacy evaluation using mouse pancreatic tumor models

The bilateral subcutaneous pancreatic tumor-bearing C57BL/6 mice were randomly divided into ten groups. The mice in each group were systematically injected with 0.2 mL saline, PBS solution of SPINs (0.6 mg/mL, on days 0, 3, and 6) or free-drug mixture (4 mg/kg body weight for NLG919 and aPD-L1, on day 0, 3, and 6) via tail vein. At 24 h post-injection, the primary tumors were exposed under US irradiation (1.0 MHz, 1.2 W/cm^2^, 50% duty cycle) for 10 min on days 1, 4, and 7. The volumes of primary and distant tumors (*n* = 6) were calculated by measuring the lengths and widths of tumors using a caliper every 2 days for 18 days. The survival of mice in each group (*n* = 10) were monitored and the survival rates were expressed using Kaplan–Meier plots.

### Histological analysis of tumors

After treatments as above described for 10 days, the mice in each group were euthanized, and the primary and distant tumors were collected for H&E staining and immunofluorescence TUNEL staining.

### In vivo antitumor efficacy evaluation using rechallenged tumor models

The subcutaneous pancreatic tumor-bearing C57BL/6 mice were randomly divided into two groups. The mice in each group were systematically injected with 0.2 mL saline or PBS solution of SPIN_D2_ (0.6 mg/mL, on days 0, 3, and 6) via tail vein. For US treatment groups, the tumors were exposed under US irradiation (1.0 MHz, 1.2 W/cm^2^, 50% duty cycle, 10 min) on day 1, 4, and 7. On day 8, 2 × 10^6^ Panc02 cancer cells were subcutaneously injected into the left flank of the mouse as the rechallenged tumors. The sizes of rechallenged tumors (*n* = 5) were measured using caliper every 2 days for 14 days to calculate tumor volumes. The survival of mice in each group (*n* = 10) was monitored and the survival rates were expressed using Kaplan–Meier plots.

### Gene expression analysis

Subcutaneous pancreatic tumor-bearing C57BL/6 mice (*n* = 5) in each group after treatments for 7 days were euthanized to extract tumors for gene expression analysis. Total RNA was extracted from tumor tissues using TRIzol® Reagent (Invitrogen, cat. no. 15596026) according to the manufacturer’s instructions. RNA purification, reverse transcription, library construction, and sequencing were performed according to the manufacturer’s instructions. Complementary DNA (cDNA) was synthesized using a SuperScript double-stranded cDNA synthesis kit (Invitrogen, cat. no. 11917020) with Random Hexamer primers (Invitrogen, cat. no. N8080127).

### Flow cytometry analysis

Bilateral subcutaneous pancreatic tumor-bearing C57BL/6 mice (*n* = 5) in each group after treatments were euthanized to extract tumor-draining lymph nodes, spleen, and tumors. The collected tissues were cut into small pieces and ground into tissue suspension in an ice box. The tissue suspension was filtered through 70-μm cell strainers to obtain single-cell suspension, followed by treatments with red blood cell lysis buffer or lymphocyte separation medium and washing with PBS to separate lymphocytes. The harvested cells were then stained with fluorescence-labeled antibodies following the manufacturer’s instructions. The antibodies were listed as follows: CD45-BV605 (Biolegend, cat. no. 103140, clone 30-F11), CD11c-FITC (Biolegend, cat. no. 117306, clone N418), CD80-PE (Biolegend, cat. no. 104708, clone 16-10A1), CD86-APC (Biolegend, cat. no. 105114, clone PO3), CD3-APC/Cyanine7 (Biolegend, cat. no. 100222, clone 17A2), CD4-FITC (Biolegend, cat. no. 100406, clone GK1.5), CD8-PE (Biolegend, cat. no. 140408, clone 53-5.8), Foxp3-PE (Biolegend, cat. no. 126404, clone MF-14), CD25-APC (Biolegend, cat. no. 101910, clone 3C7), CD44-APC (Biolegend, cat. no. 103011, clone IM7), and CD26L-PerCP/Cyanine5.5 (Biolegend, cat. no. 104431, clone MEL-14). All antibodies used in the experiments were diluted ~100 times. As a control, cells were stained with fluorescence-labeled armenian hamster IgG isotype control antibodies or rat IgG2b,κ isotype control antibodies. The isotype control antibodies were listed as follows: Brilliant Violet 605™ Rat IgG2b, κ Isotype Ctrl (Biolegend, cat. no. 400650, clone RTK4530), APC/Cyanine7 Rat IgG2b, κ Isotype Ctrl (Biolegend, cat. no. 400624, clone RTK4530), FITC Rat IgG2b, κ Isotype Ctrl (Biolegend, cat. no. 400605, clone RTK4530), PE Rat IgG2b, κ Isotype Ctrl (Biolegend, cat. no. 400608, clone RTK4530), APC Rat IgG2b, κ Isotype Ctrl (Biolegend, cat. no. 400612, clone RTK4530), PerCP/Cyanine5.5 Rat IgG2a, κ Isotype Ctrl (Biolegend, cat. no. 400531, clone RTK2758), FITC Armenian Hamster IgG Isotype Ctrl (Biolegend, cat. no. 400905, clone HTK888), and PE Armenian Hamster IgG Isotype Ctrl (Biolegend, cat. no. 400907, clone HTK888). All antibodies used in the experiments were diluted ~100 times. The stained cells were analyzed using a Fortessa X20 flow cytometer (BD Biosciences, USA), and the flow cytometry data were analyzed using FlowJo software (version 10.7.2). Dead cells were excluded using DAPI or Zombie Aqua™ (Biolegend, cat. no. 423102) staining for analysis.

### In vivo deep-tissue therapy of pork tissue-covered tumor models

After systematical injection of 0.2 mL saline, PBS solution of SPIN_0_ or SPIN_D2_ (0.6 mg/mL, on day 0, 3, and 6) or free-drug mixture (4 mg/kg body weight for NLG919 and aPD-L1, on day 0, 3, and 6) into the bilateral subcutaneous pancreatic tumor-bearing C57BL/6 mice via tail vein, the primary tumors covered with 5-cm pork tissues were exposed under US irradiation (1.0 MHz, 1.2 W/cm^2^, 50% duty cycle) for 10 min on day 1, 4 and 7. After treatments, the growth of primary and distant tumors (*n* = 5) and survival of mice in each group (*n* = 10) were monitored.

### Establishment of rabbit orthotopic pancreatic tumor models

The rabbit experiments were performed according to the protocols authorized by the Institutional Animal Care and Use Committee at Tongji University. Female New Zealand white rabbits (age: 9–12 weeks, weight: 2.0–2.5 kg) were purchased from Shanghai Slac Laboratory Animal Center. Rabbit VX2 liver cancer cells (MZ-0769) were obtained from Ningbo Mingzhou Biological Technology Co., Ltd. VX2 tumor cell suspension was obtained from VX2 tumors after homogenization and filter through 40-μm mesh cell strainers. The VX2 tumor cell solution was implanted into hindlimbs of donor rabbits to develop tumors for 2–3 weeks. To establish rabbit orthotopic pancreatic tumor models, and an incision was made through the upper median line of the abdomen to expose the pancreas, followed by making a small stab wound in the pancreas parenchyma. VX2 tumors were extracted from tumor-bearing donor rabbits and then cut into 1-mm^3^ fragments. The VX2 tumor fragments were implanted into stab wounds in the pancreas. After implantation, absorbable hemostats were put onto the incision sites, and the abdomen wall and skin were closed. The rabbit orthotopic pancreatic tumor models were used for experiments after 10 days of implantation.

### Synthesis of ^131^I-SPIN_0_

To synthesize ^131^I-labeled SPIN_0_, 10 mL PBS solution of SPIN_0_ (2 mg/mL) was mixed with Na^131^I (20 mCi) and chloramine-T (16 mg) in PBS solution and reacted at room temperature for 30 min. After purification using filters by centrifugation to remove excess ^131^I till no gamma activity was detected in the filtrate solution, ^131^I- SPIN_0_ was obtained. The radiolabeling yield of ^131^I- SPIN_0_ was 70%.

### SPECT imaging of rabbit orthotopic pancreatic tumor models

The orthotopic pancreatic tumor-bearing rabbits were systematically injected with ^131^I-SPIN_0_ (1.0 mL, 1.5 mg/mL) via ear vein, and SPECT imaging was conducted at 2, 12, and 24 h post-injection using a NanoSPECT/CT animal imaging system (Bioscan Ltd., Washington, DC) with a tube voltage of 80 kV, tube current of 450 μA, and slice thickness of 45 μm. The signal intensity of radioactivity in tumor regions was measured.

### In vivo deep-tissue therapy of rabbit orthotopic pancreatic tumor models

The orthotopic pancreatic tumor-bearing rabbits were randomly divided into four groups (*n* = 4) and the rabbits in each group were systematically injected with 1.0 mL saline or PBS solution of SPIN_0_ (1.5 mg/mL, on days 0, 3, and 6) via ear vein. On days 1, 4, and 7, the tumors were irradiated by US (1.0 MHz, 1.2 W/cm^2^, 50% duty cycle, 30 min). After treatments for 5, 10, and 15 days, CT imaging was conducted to evaluate the volumes of tumors. The survival of rabbits in each group (*n* = 4) was monitored for 20 days. The tumors were collected for H&E and immunofluorescence TUNEL staining after different treatments.

### Evaluation of irAEs

The subcutaneous pancreatic tumor-bearing C57BL/6 mice were randomly divided into six groups and systematically injected with 0.2 mL saline, PBS solution of SPIN_0_, SPIN_D2_ (1.2 mg/mL, on days 0, 3, 6, 9, 12, 15, and 18) or free-drug mixture (8 mg/kg body weight for NLG919 and aPD-L1, on day 0, 3, 6, 9, 12, 15, and 18) via tail vein. US irradiation of tumors was conducted on day 1, 4, 7, 10, 13, 16, and 19 (1.0 MHz, 1.2 W/cm^2^, 50% duty cycle, 10 min). The body of mice was measured every 2 days for 18 days. After treatments for 30 days, the mice in each group (*n* = 4) were used to collect blood and spleen for flow cytometry analysis of CD3^+^CD4^+^ and CD3^+^CD8^+^ T cells after staining with antibodies as described above. The major organs (heart, liver, spleen, lung, and kidney) were collected for H&E and immunofluorescence staining. The cytokine levels in the serum of mice (*n* = 5) after different treatments were measured using LEGENDplex™ mouse inflammation panel (13-plex) with a filter plate (Biolegend, cat. no. 740150) according to the manufacturer’s protocol. The blood from mice in each group (*n* = 5) was collected for blood routine and biochemical analysis.

### Data analysis

All experiments were repeated at least three times and the results were expressed as the mean ± standard deviation (SD) unless stated otherwise. The statistical comparisons between two groups were performed using *t* test and more than three groups were determined by one-way ANOVA. For all tests, differences were considered statistically significant when *P* < 0.05, *P < 0.05, **P < 0.01 and ***P < 0.001.

### Reporting summary

Further information on research design is available in the Nature Research Reporting Summary linked to this article.

## Supplementary information

Supplementary Information

Reporting Summary

## Data Availability

The RNA sequencing data have been deposited in the Genome Sequence Archive (GSA) database under the accession code CRA007151. The remaining data are available within the Article, Supplementary Information, or Source Data file. Source data are provided with this paper.

## Source data

Source Data
