# Supplementary material for: Precision cancer sono-immunotherapy using deep-tissue activatable semiconducting polymer immunomodulatory nanoparticles
Source: Nat Commun. 2022 Jul 12;13:4032. doi: 10.1038/s41467-022-31551-6 (PMC9276830; doi:10.1038/s41467-022-31551-6)
Supplement: Supplementary file 1 — Supplementary Information [file 41467_2022_31551_MOESM1_ESM.pdf]

Supplementary Information For

Precision Cancer Sono-immunotherapy Using Deep-tissue Activatable Semiconducting Polymer immunomodulatory nanoparticles

Jingchao Li[1], Yu Luo[2], Ziling Zeng[1], Dong Cui[1], Jiaguo Huang[1], Chenjie Xu[1], Liping Li[3], Kanyi Pu[1,*], and Ruiping Zhang[3,*]

[1] School of Chemical and Biomedical Engineering, Nanyang Technological University, 70 Nanyang Drive, Singapore 637457, Singapore.

[2] School of Chemical Science and Engineering, Tongji University, 1239 Siping Road, Shanghai, 200092, China.

[3] The Third Hospital of Shanxi Medical University, Shanxi Bethune Hospital, Shanxi Academy of Medical Sciences, Taiyuan, 030032, China.

*E-mail: kypu@ntu.edu.sg; zrp_7142@sxmu.edu.cn

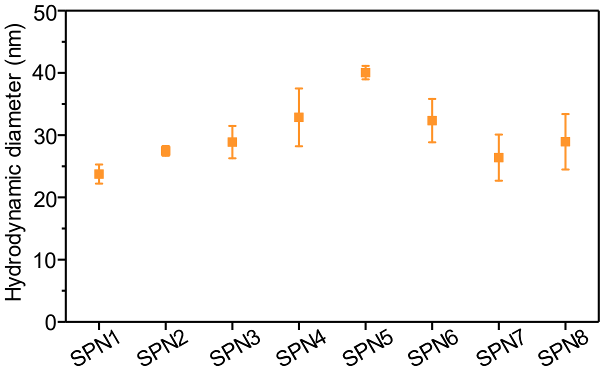

Supplementary Figure 1. Hydrodynamic diameters of SPNs. Hydrodynamic diameters of SPNs (20 μg/mL) in 1× PBS (pH = 7.4) measured by DLS (n = 3). Data are presented as mean values ± SD. Source data are provided as a Source Data file.

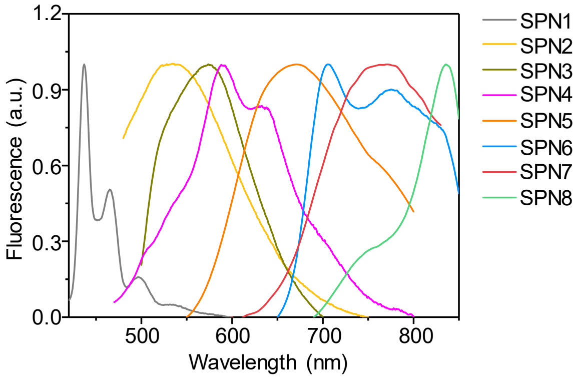

Supplementary Figure 2. Optical characterization of SPNs. Fluorescence spectra of SPNs in 1× PBS (pH = 7.4). Source data are provided as a Source Data file.

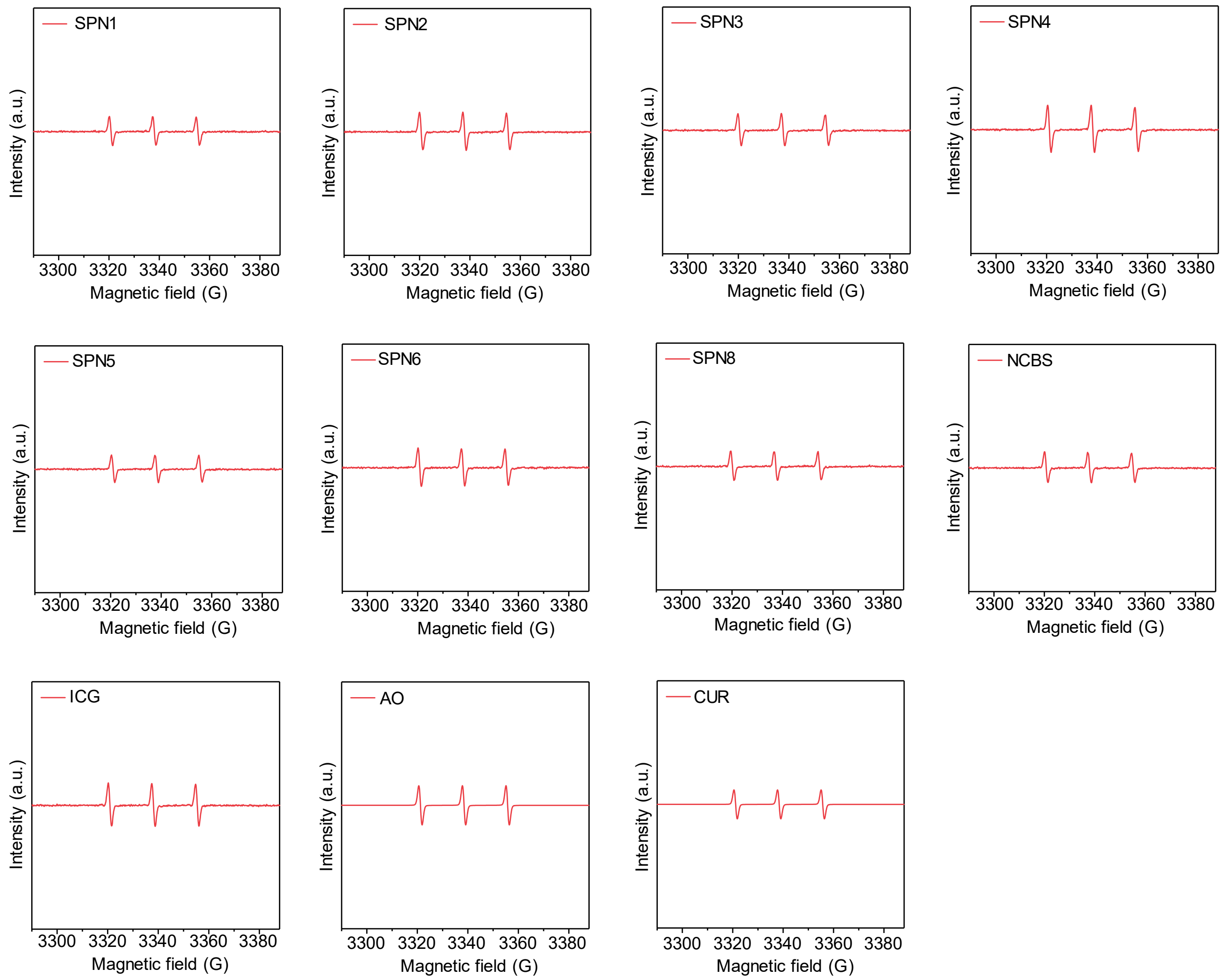

Supplementary Figure 3. Study of sonodynamic $^1O_2$ generation. ESR spectra of SPN1, SPN2, SPN3, SPN4, SPN5, SPN6, SPN8, NCBS, ICG, AO and CUR at the concentration of 20 μg/mL after US irradiation (1.0 MHz, 1.2 W/cm^2, 50% duty cycle, 5 min) using TEMP as the trap. Source data are provided as a Source Data file.

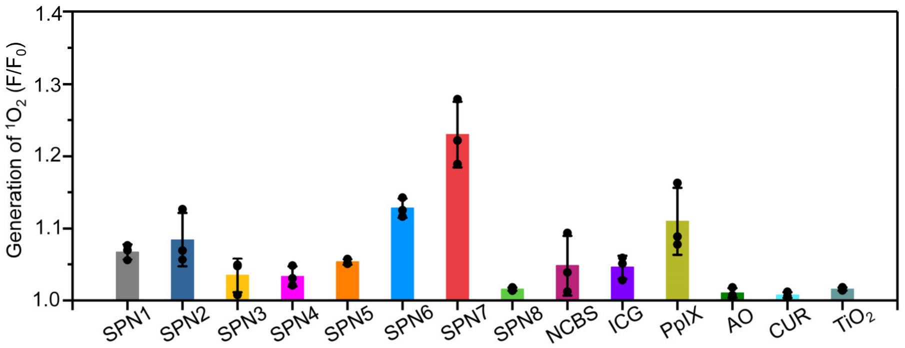

Supplementary Figure 4. Study of photodynamic $^1O_2$ generation. Fluorescence enhancement (F/F$_0$) of SOSG (1 μM) at 528 nm in solutions containing SPN1-8, NCBS, ICG, PpIX, AO, CUR or TiO_2 nanoparticles at the concentration of 20 μg/mL after white light irradiation (0.1 W/cm^2, 1 min) (n = 3). Data are presented as mean values ± SD. Source data are provided as a Source Data file.

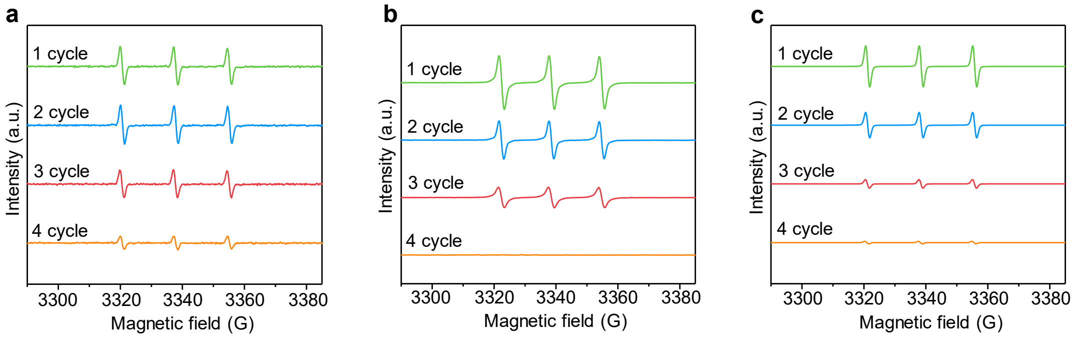

Supplementary Figure 5. Sonodynamic stability study. ESR spectra of ICG (**a**), PpIX (**b**) and AO (**c**) at the concentration of 20 μg/mL after 1, 2, 3, and 4 cycles of US irradiation (1.0 MHz, 1.2 W/cm^2, 50% duty cycle, 5 min for each cycle) with TEMP as the trap. Source data are provided as a Source Data file.

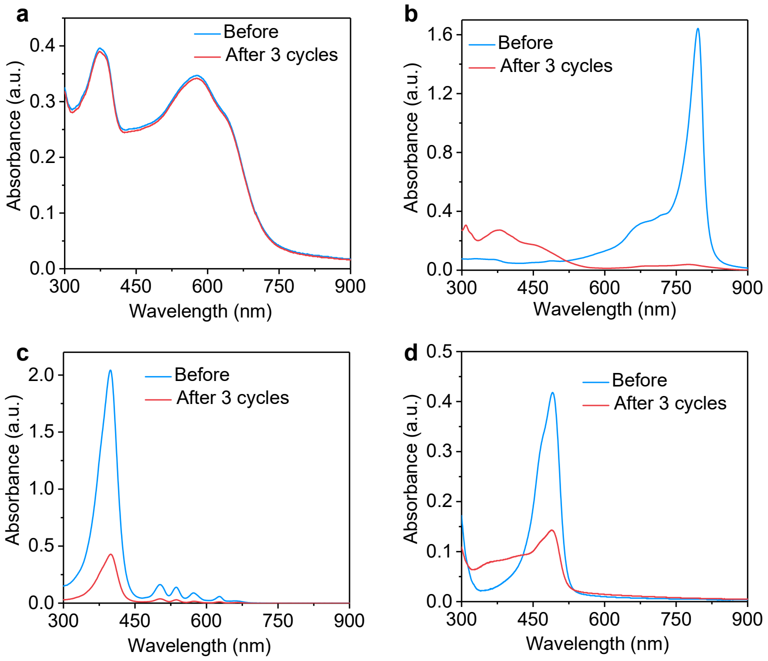

Supplementary Figure 6. Sonodynamic stability study. UV-vis absorption spectra of SPN7 (**a**), ICG (**b**), PpIX (**c**) and AO (**d**) at the concentration of 20 μg/mL after US irradiation (1.0 MHz, 1.2 W/cm^2, 50% duty cycle, 5 min for each cycle). Source data are provided as a Source Data file.

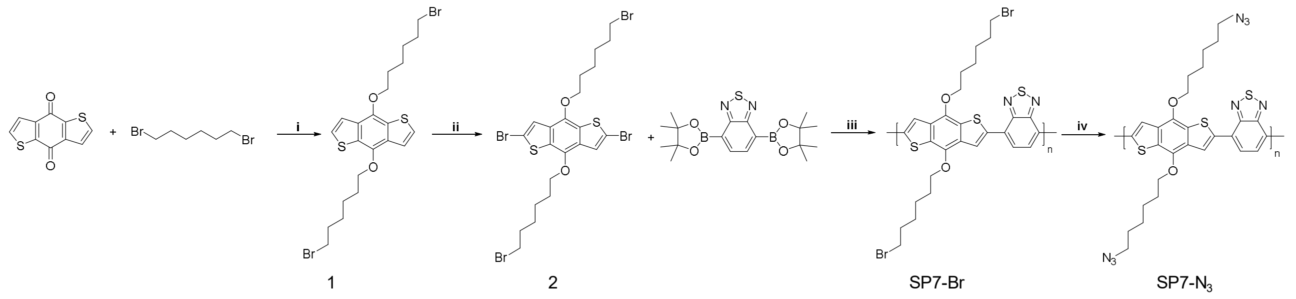

Supplementary Figure 7. Synthesis of SP7-N$_3$. Reagents and conditions: i) Zn, NaOH, TBAB, H_2O, 100 °C, 18 h; ii) Br_2, DCM, room temperature, 6 h; iii) $Pd(PPh_3)_4$, K_2CO_3, methyltrioctylammonium

chloride, 100 ºC, 24 h; iv) NaN_3, THF/DMF, room temperature, 12 h.

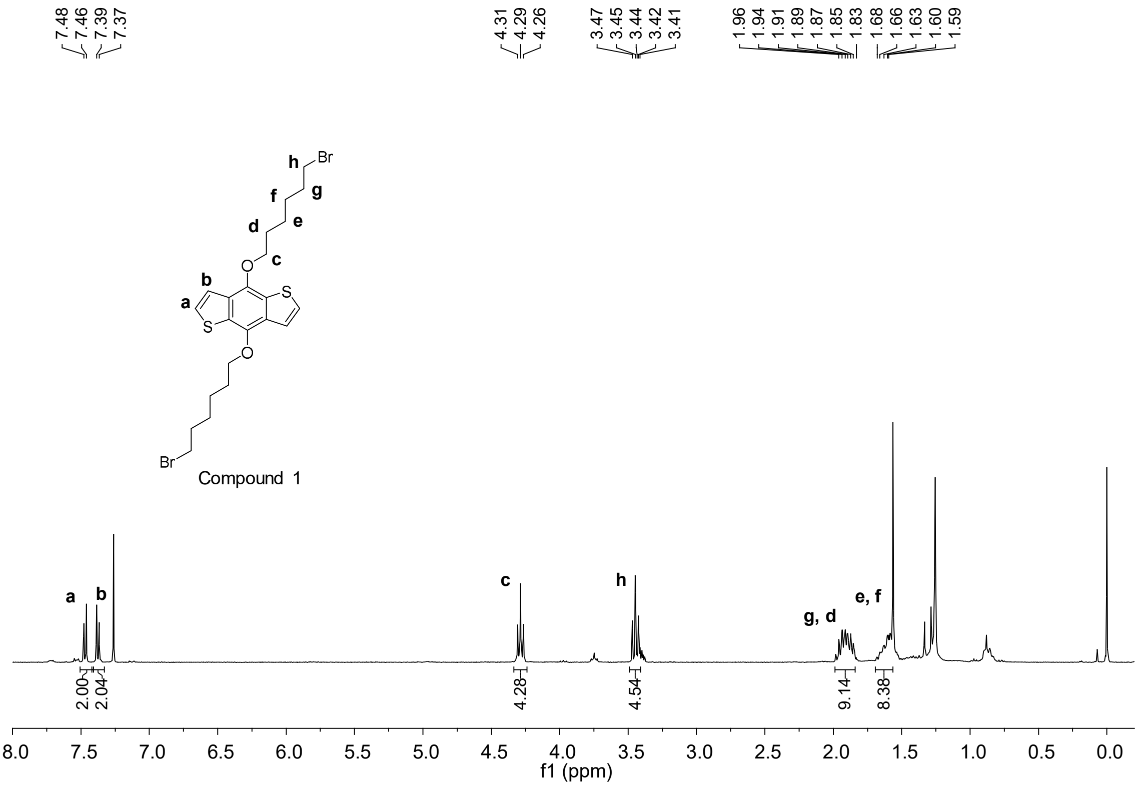

Supplementary Figure 8. ^{1}H NMR characterization. ^{1}H NMR spectrum of compound 1 in $CDCl_3$.

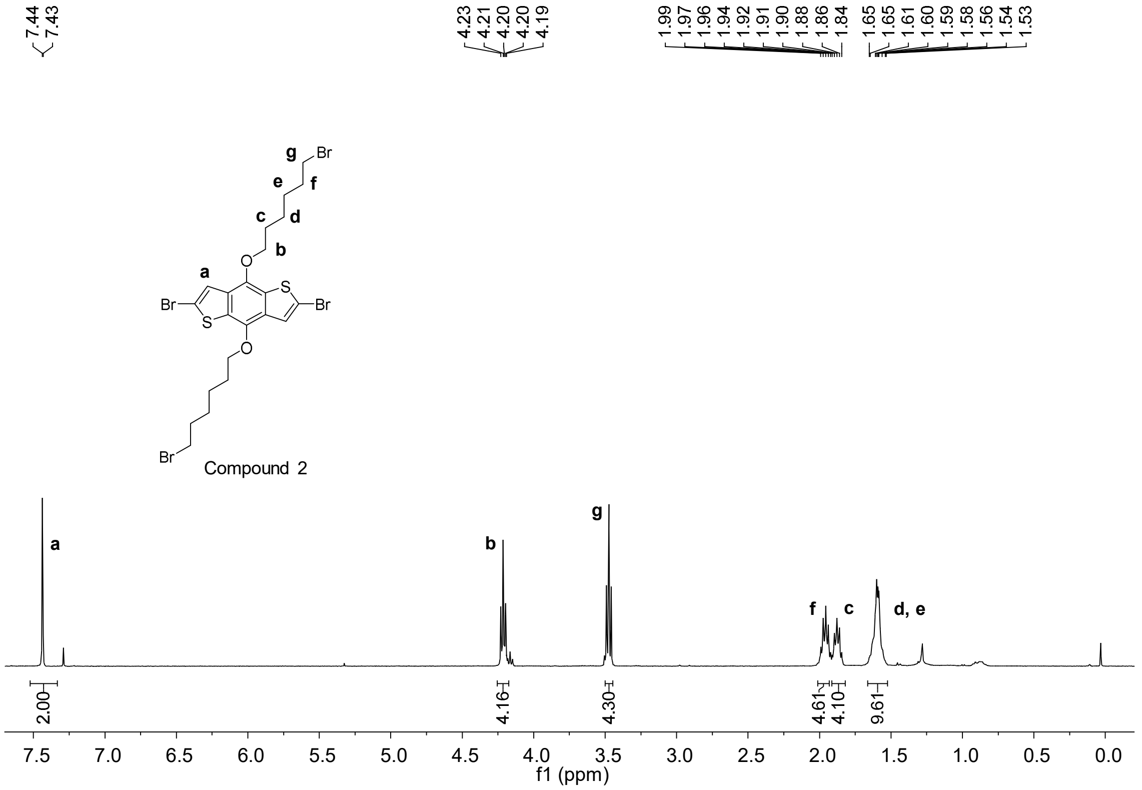

Supplementary Figure 9. ^{1}H NMR characterization. ^{1}H NMR spectrum of compound 2 in $CDCl_3$.

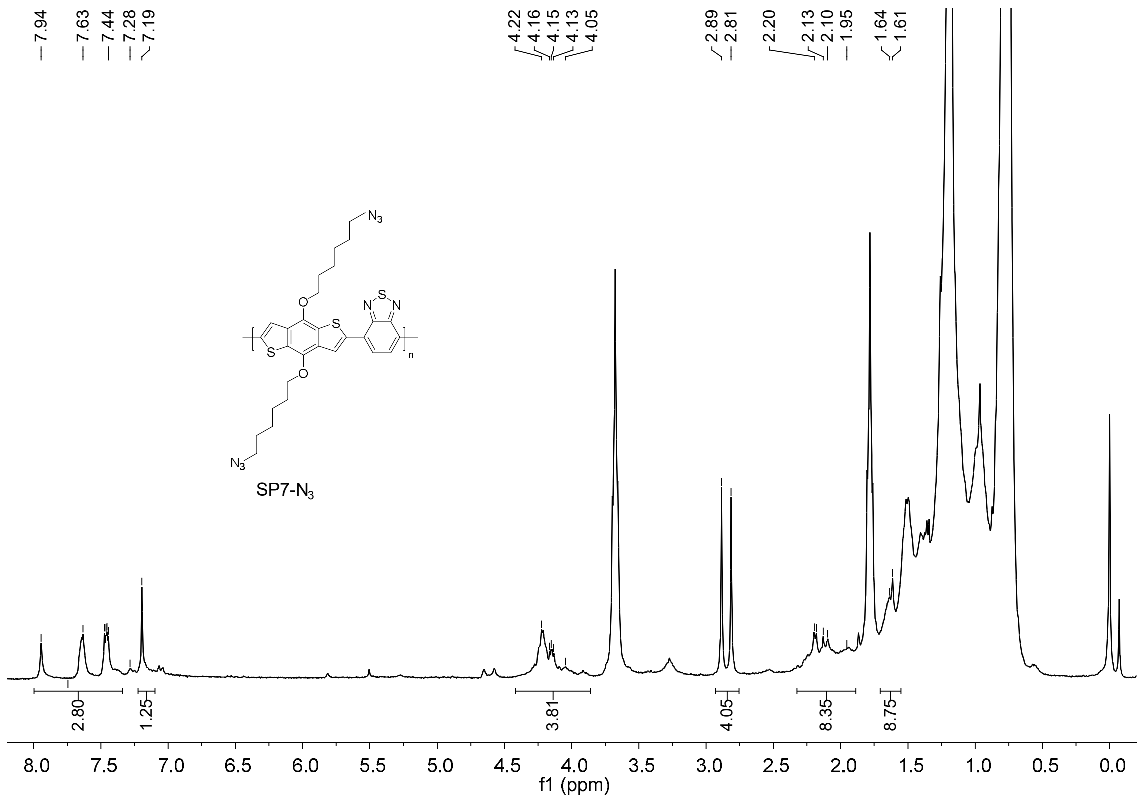

Supplementary Figure 10. ^{1}H NMR characterization. ^{1}H NMR spectrum of SP7-N_3 in $CDCl_3$.

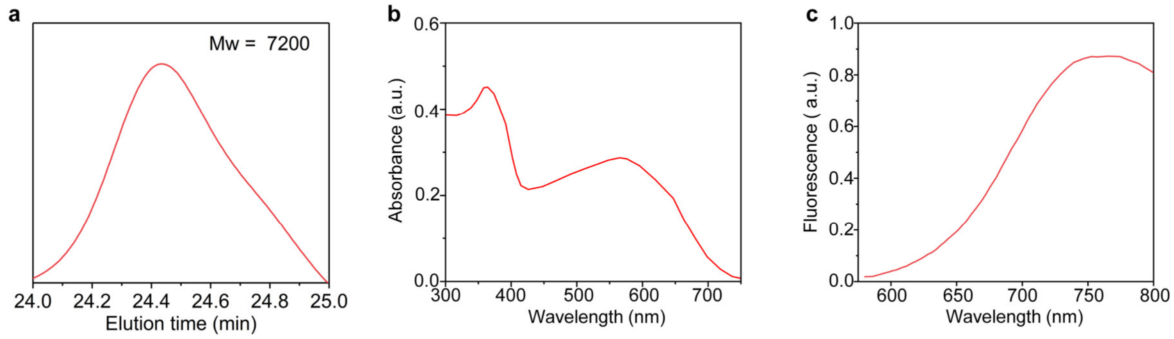

Supplementary Figure 11. Characterization of SP7-N_3. Gel permeation chromatography (GPC) curve (**a**), UV-vis absorption spectrum (**b**), and fluorescence spectrum (**c**) of SP7-N_3 in THF. Source data are provided as a Source Data file.

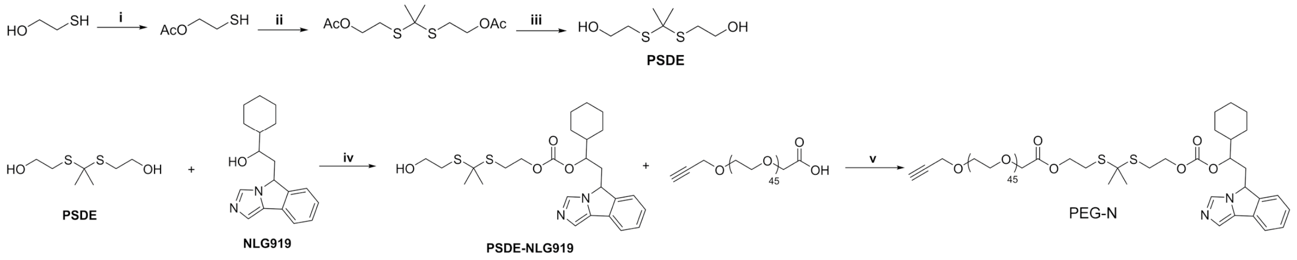

Supplementary Figure 12. Synthesis of PEG-N. Reagents and conditions: i) Ac_2O, $TiCl_3(OTf)$, room temperature, 3 h. ii) Acetone, TFA, room temperature, 24 h. iii) KOH, MeOH, room temperature, 16 h. (iv) BTC, TEA, DMAP, DCM, room temperature, 10 h; v) EDCI, DMAP, DCM, room temperature, 12 h.

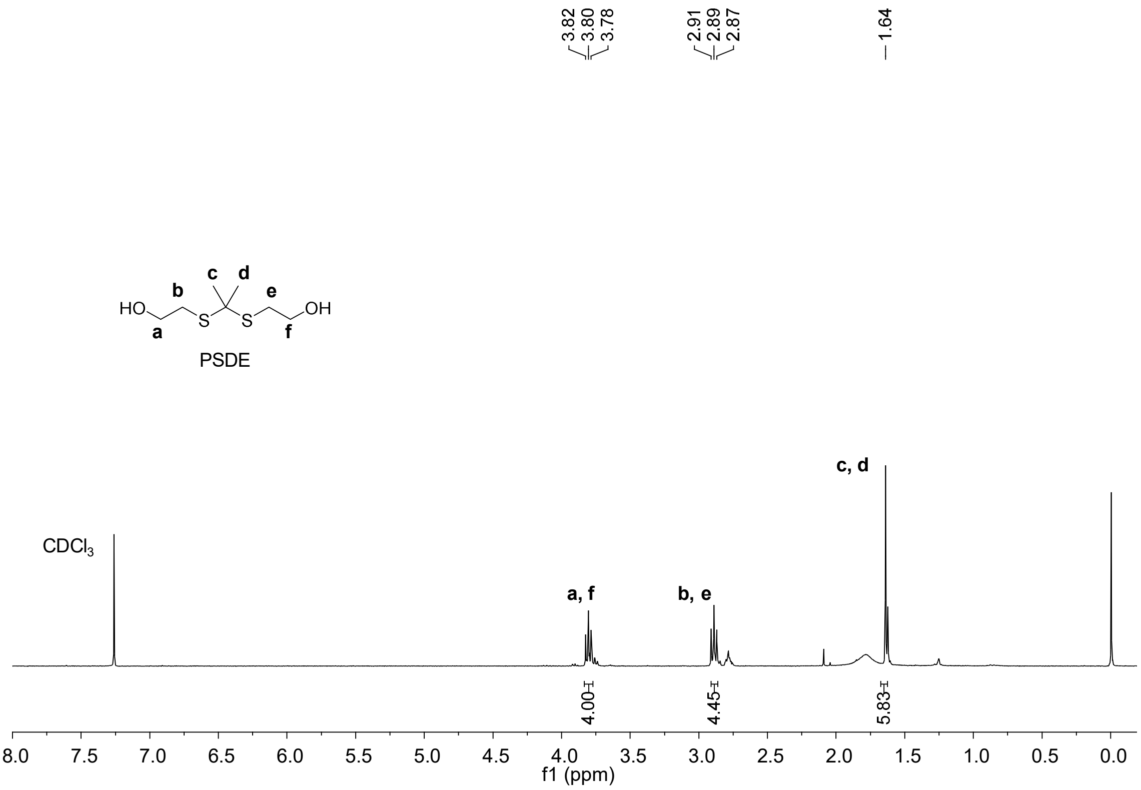

Supplementary Figure 13. ^{1}H NMR characterization. ^{1}H NMR spectrum of PSDE in $CDCl_3$.

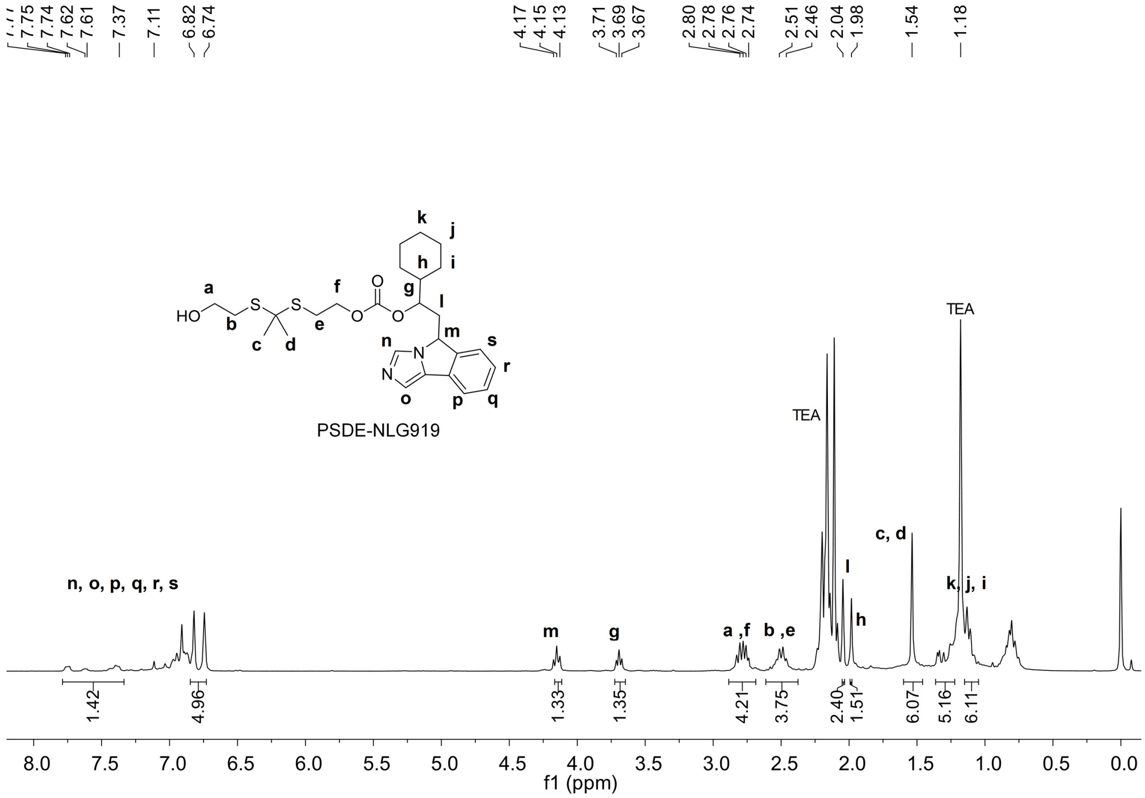

Supplementary Figure 14. ^{1}H NMR characterization. ^{1}H NMR spectrum of PSDE-NLG919 in $CDCl_3$.

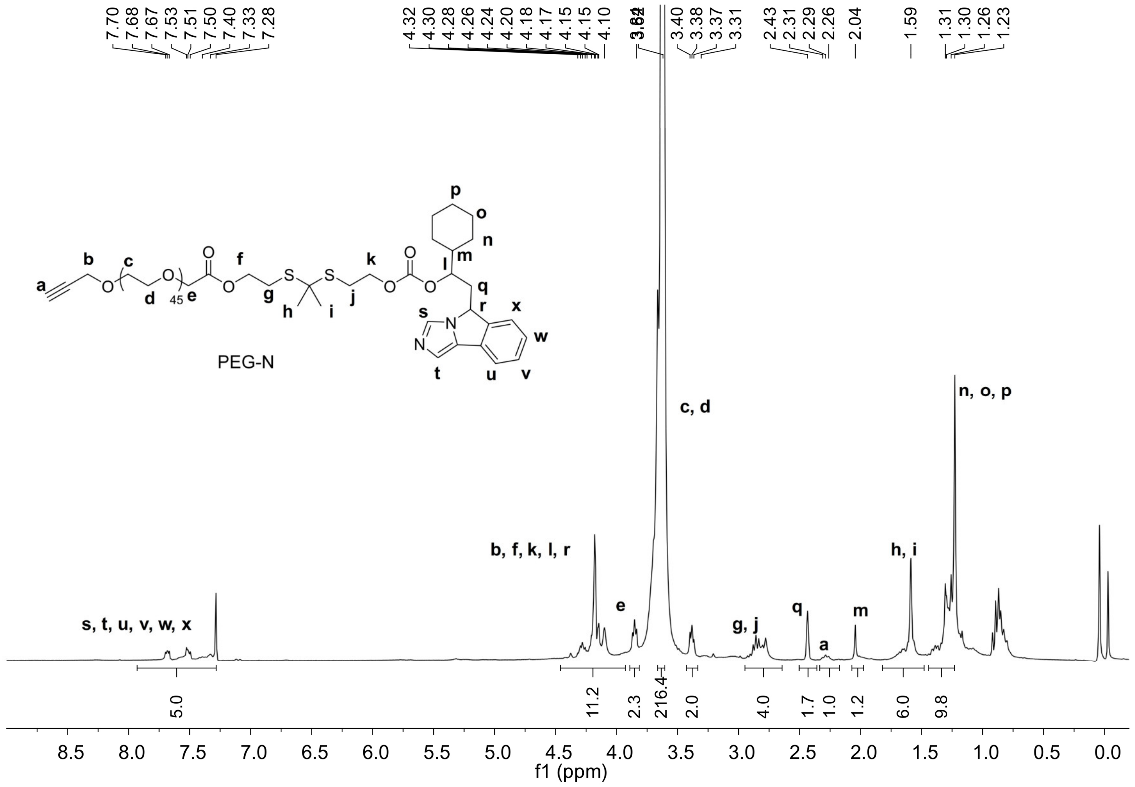

Supplementary Figure 15. ^{1}H NMR characterization. ^{1}H NMR spectrum of PEG-N in $CDCl_3$.

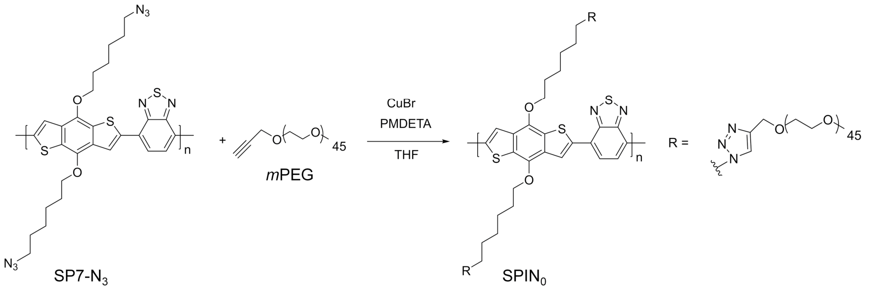

Supplementary Figure 16. Synthesis of $SPIN_0$. Reagents and conditions: copper(I)bromide (CuBr), N,N,N’,N’’,N’’-pentamethyldiethylenetriamin (PMDETA), THF, room temperature, 24 h.

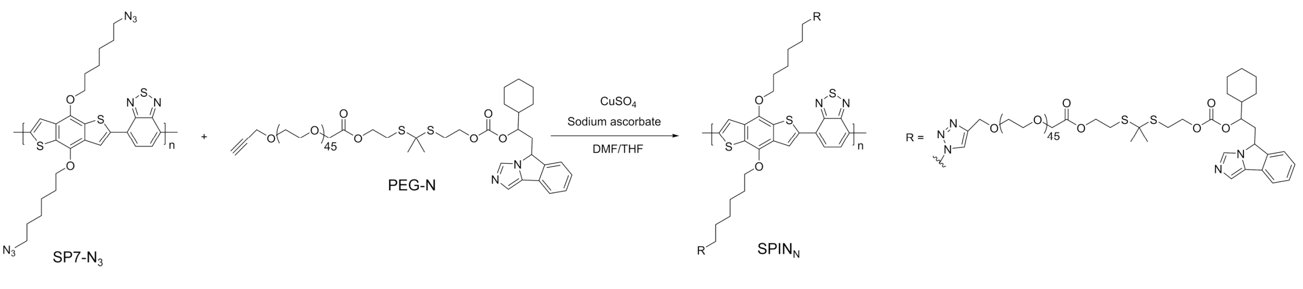

Supplementary Figure 17. Synthesis of $SPIN_N$. Reagents and conditions: $CuSO_4$, sodium ascorbate, DMF/THF, room temperature, 24 h.

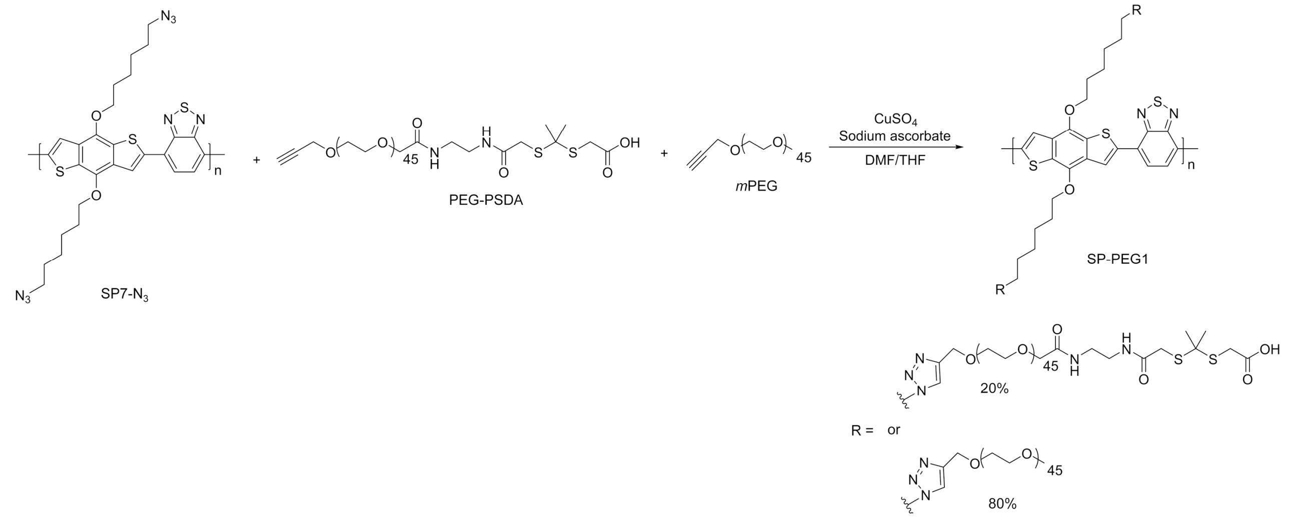

Supplementary Figure 18. Synthesis of SPN-PEG1. Reagents and conditions: $CuSO_4$, sodium ascorbate, DMF/THF, room temperature, 24 h.

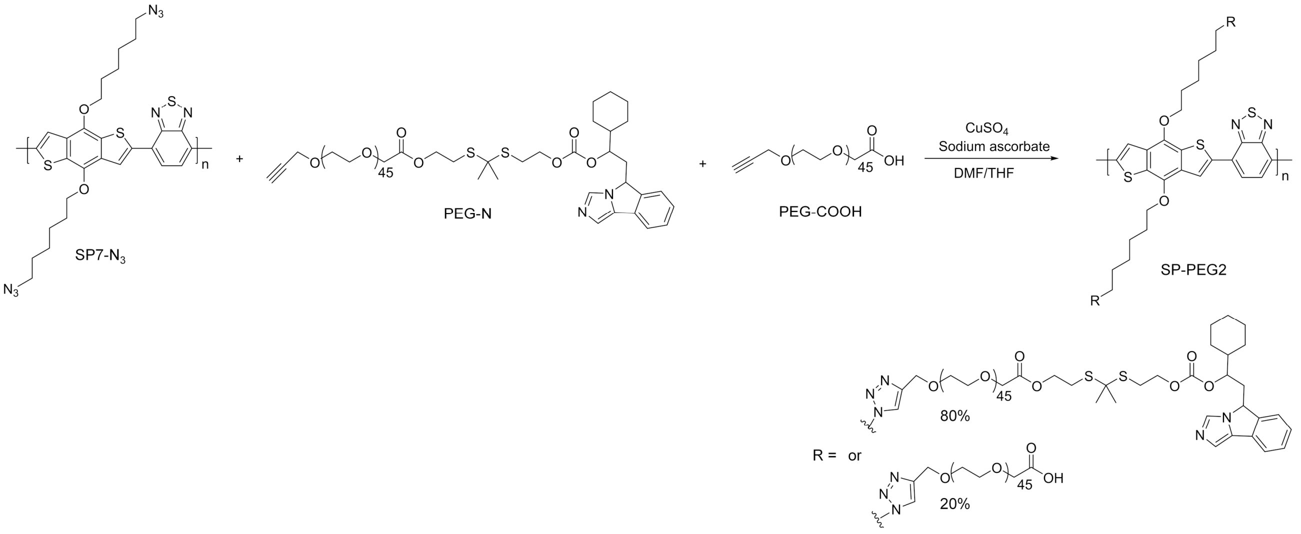

Supplementary Figure 19. Synthesis of SPN-PEG2. Reagents and conditions: $CuSO_4$, sodium ascorbate, DMF/THF, room temperature, 24 h.

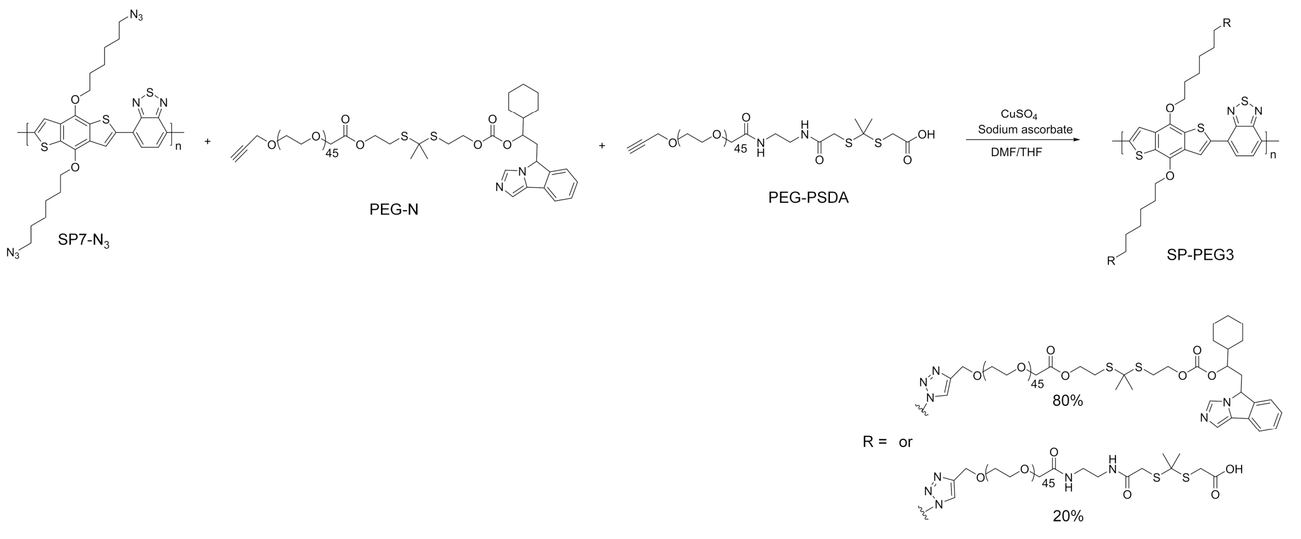

Supplementary Figure 20. Synthesis of SPN-PEG3. Reagents and conditions: $CuSO_4$, sodium ascorbate, DMF/THF, room temperature, 24 h.

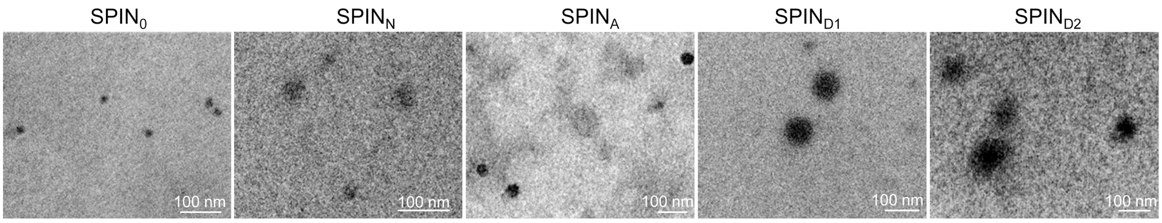

Supplementary Figure 21. Morphology characterization of SPINs. Representative TEM images of SPIN$_0$, SPIN$_N$, SPIN$_A$, SPIN$_{D1}$, and SPIN$_{D2}$. The experiments were repeated independently three times with similar results.

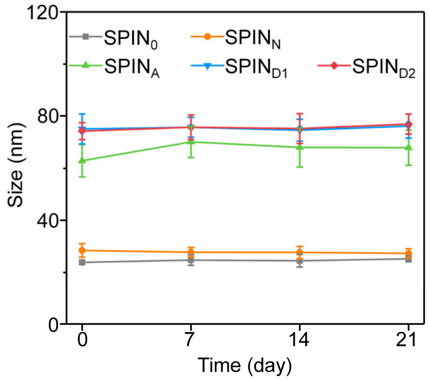

Supplementary Figure 22. Colloidal stability study of SPINs. Hydrodynamic sizes of SPINs after different days of storage in 1× PBS buffer (pH = 7.4) (n = 3). Data are presented as mean values ± SD. Source data are provided as a Source Data file.

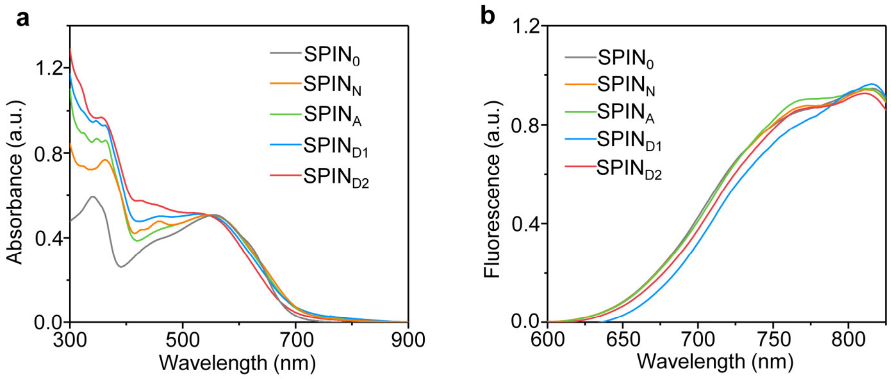

Supplementary Figure 23. Optical property characterization of SPINs. UV-vis absorption (**a**) and fluorescence (**b**) spectra of SPINs in 1× PBS buffer (pH = 7.4). Source data are provided as a Source Data file.

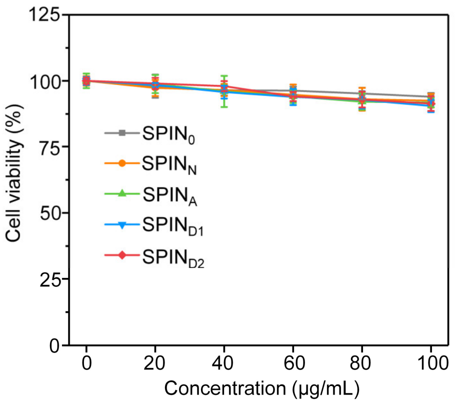

Supplementary Figure 24. Cytotoxicity assay of SPINs. In vitro cell viability of Panc02 cancer cells after treatment with SPINs at the final concentrations of 0, 20, 40, 60, 80 and 100 μg/mL for 24 h. The cells without treatment were used as control. The cell viability was measured using CCK-8 kit (n = 5). Data are presented as mean values ± SD. Source data are provided as a Source Data file.

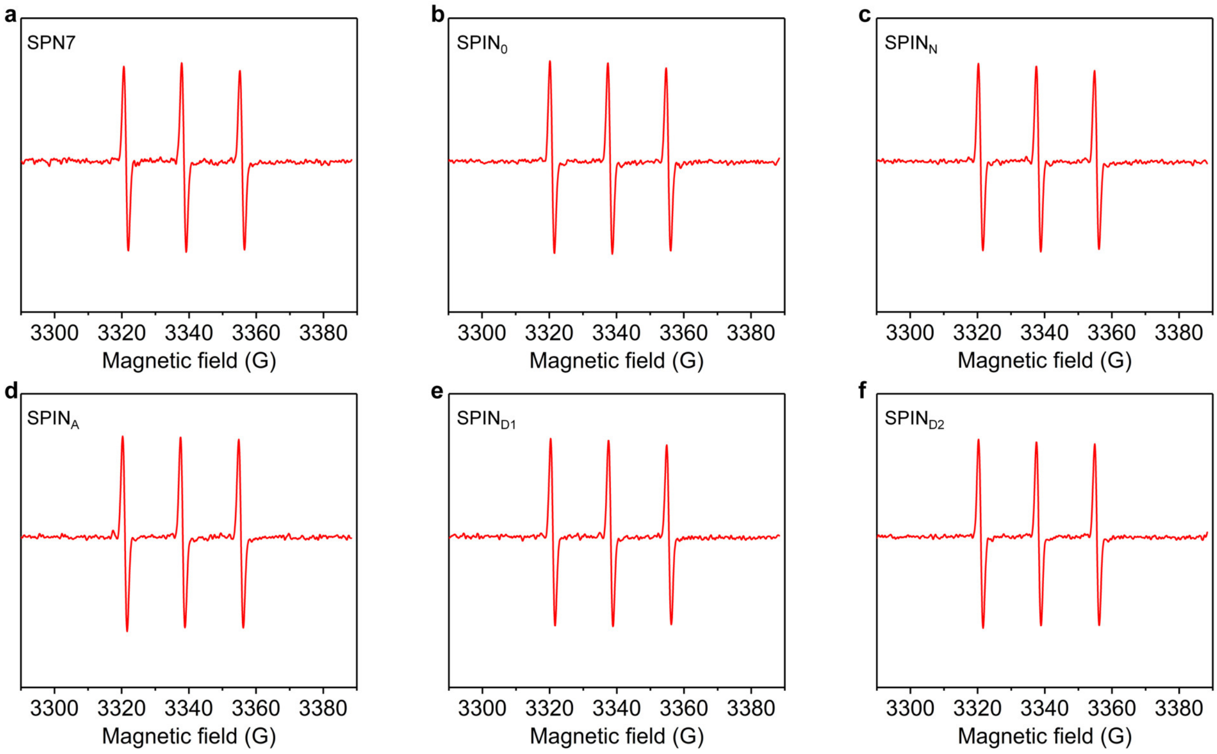

Supplementary Figure 25. Study of sonodynamic $^1O_2$ generation for SPINs. ESR spectra of SPN7 (**a**), $SPIN_0$ (**b**), $SPIN_N$ (**c**), $SPIN_A$ (**d**), $SPIN_{D1}$ (**e**), and $SPIN_{D2}$ (**f**) at the same concentration (10 μg/mL) after US irradiation (1.0 MHz, 1.2 W/cm^2, 50% duty cycle, 3 min) using TEMP as the trap. Source data are provided as a Source Data file.

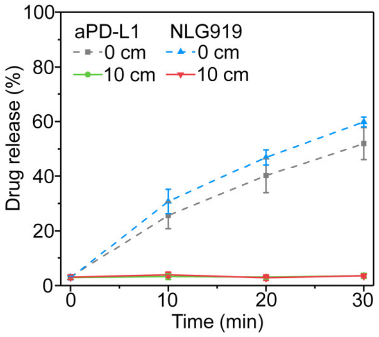

Supplementary Figure 26. Photoirradiation-induced drug release. Release profiles of aPD-L1 and NLG919 from $SPIN_{D2}$ (40 μg/mL) after white light irradiation for different time (n = 4). Data are presented as mean values ± SD. Source data are provided as a Source Data file.

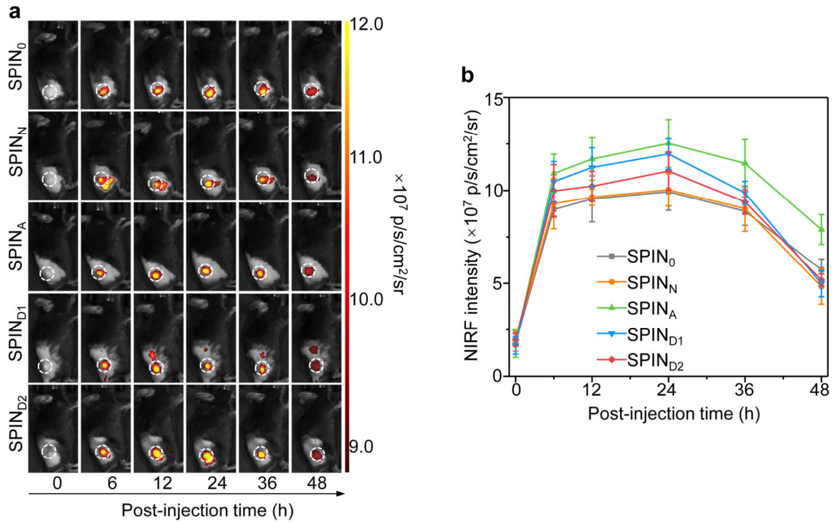

Supplementary Figure 27. In vivo NIR fluorescence imaging of tumor-bearing mice. (**a**) In vivo NIR fluorescence imaging of Panc02 tumor-bearing mice after systemic administrations of $SPIN_0$, $SPIN_N$, $SPIN_A$, $SPIN_{D1}$, and $SPIN_{D2}$ (0.2 mL, 0.6 mg/mL) for different time. The white dotted circle indicated tumors. (b) NIR fluorescence (NIRF) intensity of tumors from Panc02 tumor-bearing mice at different post-injection time (n = 3). Data are presented as mean values ± SD. Source data are provided as a Source Data file.

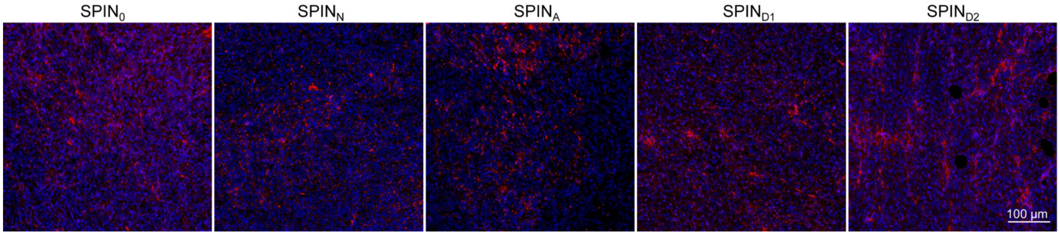

Supplementary Figure 28. Tumor accumulation of SPINs. Confocal fluorescence images of tumors from Panc02 tumor-bearing mice after systemic injection of SPINs (0.2 mL, 0.6 mg/mL) via tail vein for 24 h. The cell nucleus stained with 4’,6-diamidino-2-phenylindole (DAPI) shows blue fluorescence signal and nanoparticles shows red fluorescence signal. The experiments were repeated independently three times with similar results.

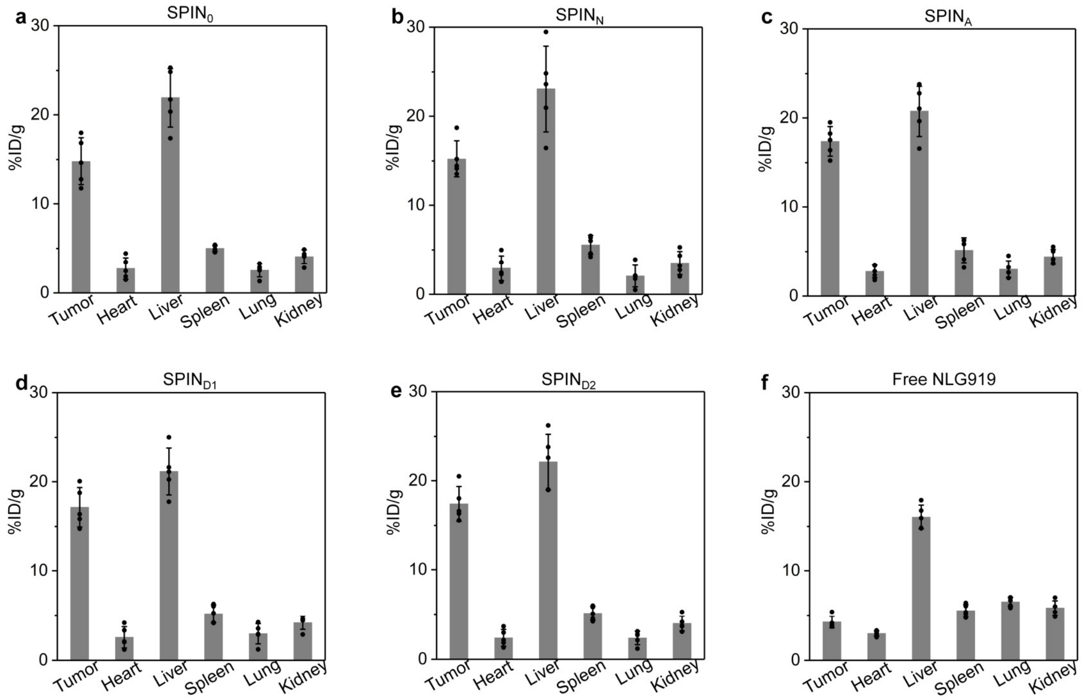

Supplementary Figure 29. In vivo biodistribution of SPINs. In vivo biodistribution of $SPIN_0$ (**a**), $SPIN_N$ (**b**), $SPIN_A$ (**c**), $SPIN_{D1}$ (**d**), $SPIN_{D2}$ (**e**) and free NLG919 (**f**) in subcutaneous Panc02 tumor-bearing mice (n = 5) at 24 h after systemic administration. Data are presented as mean values ± SD. Source data are provided as a Source Data file.

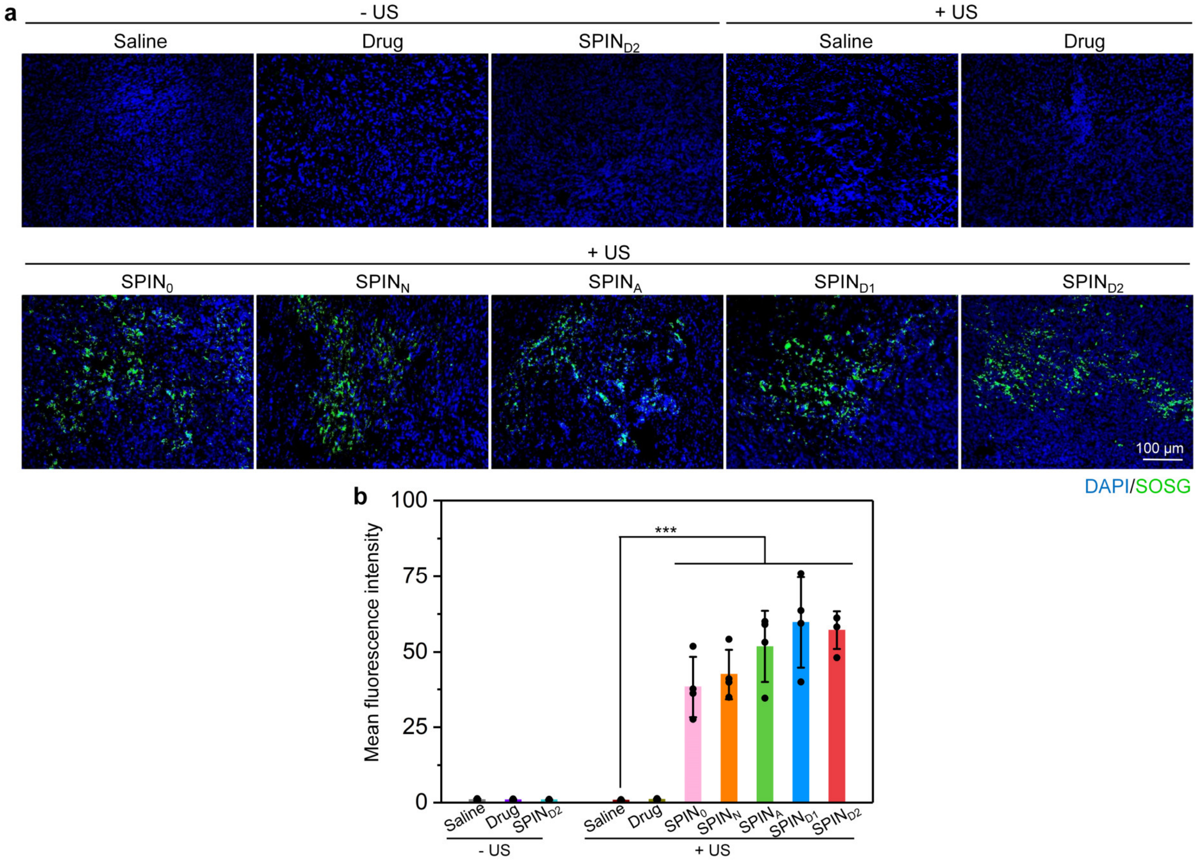

Supplementary Figure 30. Intratumor $^1O_2$ generation. (**a**) Representative confocal fluorescence

images of tumor sections from subcutaneous Panc02 tumor-bearing mice after systemic injection of saline, free drug mixture (4 mg/kg body weight for NLG919 and aPD-L1) or SPINs (0.2 mL, 0.6 mg/mL) via tail vein with or without US irradiation (1.0 MHz, 1.2 W/cm^2, 50% duty cycle, 10 min). The US irradiation of tumor tissues was conducted at 24 h post-injection time. The cell nucleus stained with DAPI shows blue fluorescence signal and SOSG shows green fluorescence signal. (**b**) Mean fluorescence intensity of SOSG in tumors of mice after different treatments (n = 4). Saline + US versus $SPIN_0$ + US: $P < 0.0001$; Saline + US versus $SPIN_N$ + US: $P < 0.0001$; Saline + US versus $SPIN_A$ + US: $P < 0.0001$; Saline + US versus $SPIN_{D1}$ + US: $P < 0.0001$; Saline + US versus $SPIN_{D2}$ + US: $P < 0.0001$. Data are presented as mean values ± SD. Statistical significance was calculated via one-way ANOVA with a Tukey post-hoc test; ***$P < 0.001$. Source data are provided as a Source Data file.

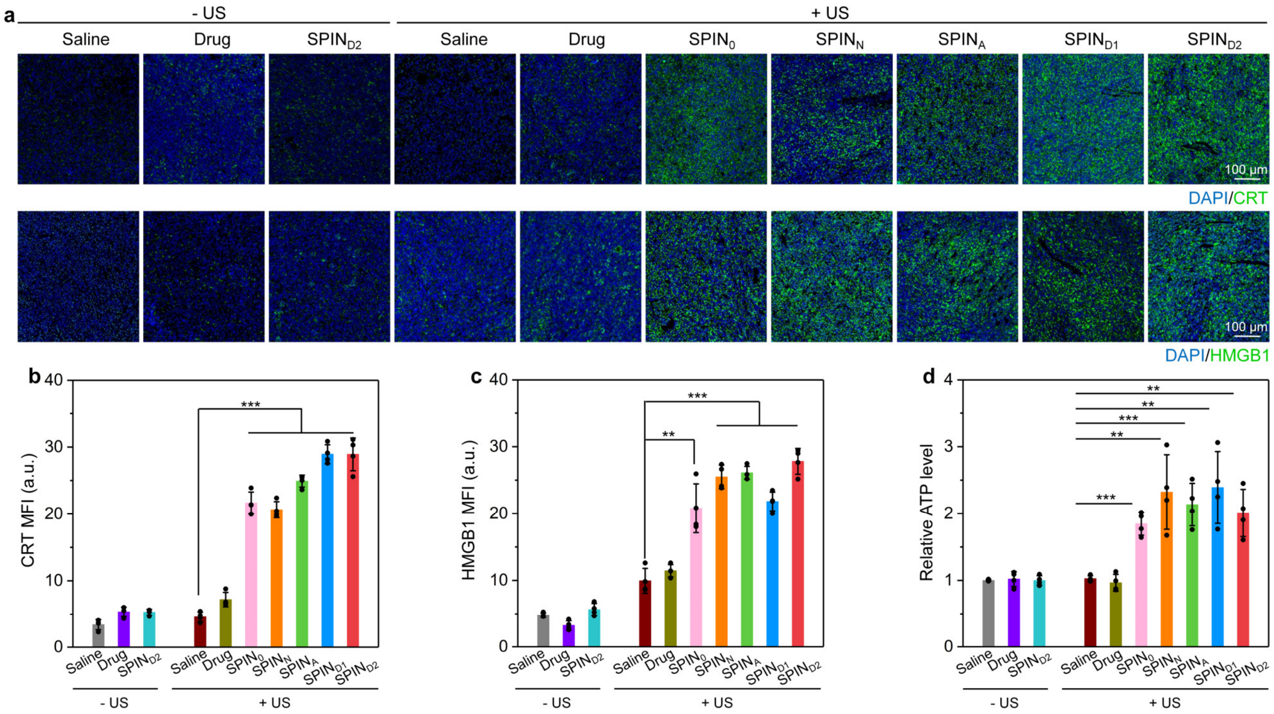

Supplementary Figure 31. Sonodynamically induced ICD. (**a**) Immunofluorescence CRT and HMGB1 staining images of tumor sections from Panc02 tumor-bearing mice after systemic injection of saline, free drug mixture (4 mg/kg body weight for NLG919 and aPD-L1) or SPINs (0.2 mL, 0.6 mg/mL) via tail vein with or without US irradiation (1.0 MHz, 1.2 W/cm^2, 50% duty cycle, 10 min). The assay was conducted at 24 h after treatments. The cell nucleus stained by DAPI shows blue fluorescence signal, CRT and HMGB1 stained by corresponding antibodies show green fluorescence signal. (**b-c**) Mean fluorescence intensity (MFI) of CRT (**b**) and HMGB1 (**c**) staining in tumor sections from different groups (n = 4). Saline + US versus $SPIN_0$ + US: $P < 0.0001$; Saline +

US versus $SPIN_N$ + US: $P < 0.0001$; Saline + US versus $SPIN_A$ + US: $P < 0.0001$; Saline + US versus $SPIN_{D1}$ + US: $P < 0.0001$; Saline + US versus $SPIN_{D2}$ + US: $P < 0.0001$ for CRT (**b**); Saline + US versus $SPIN_0$ + US: $P = 0.0018$; Saline + US versus $SPIN_N$ + US: $P < 0.0001$; Saline + US versus $SPIN_A$ + US: $P < 0.0001$; Saline + US versus $SPIN_{D1}$ + US: $P < 0.0001$; Saline + US versus $SPIN_{D2}$ + US: $P < 0.0001$ for HMGB1 (**c**). (**d**) Relative ATP levels in subcutaneous Panc02 tumors (n = 4) after different treatments for 24 h. Saline + US versus $SPIN_0$ + US: $P < 0.0001$; Saline + US versus $SPIN_N$ + US: $P = 0.0036$; Saline + US versus $SPIN_A$ + US: $P < 0.0001$; Saline + US versus $SPIN_{D1}$ + US: $P = 0.0023$; Saline + US versus $SPIN_{D2}$ + US: $P = 0.0015$. Data are presented as mean values ± SD. Statistical significance was calculated via one-way ANOVA with a Tukey post-hoc test; ** $P < 0.01$, *** $P < 0.001$. Source data are provided as a Source Data file.

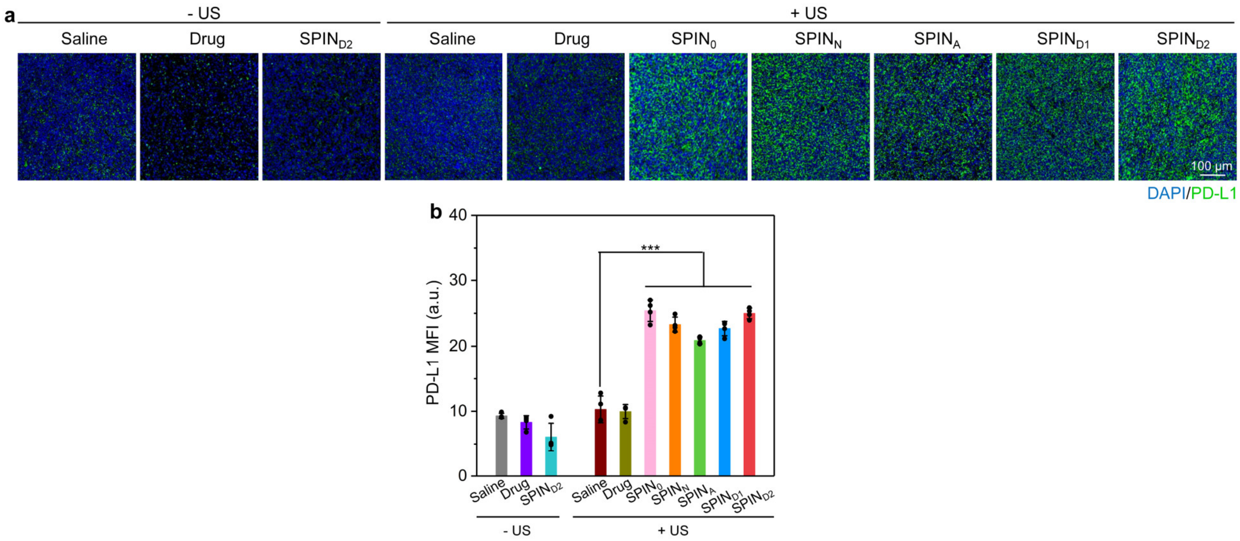

Supplementary Figure 32. Evaluation of PD-L1 expression. (**a**) Immunofluorescence PD-L1 staining images of tumor sections from Panc02 tumor-bearing mice after systemic injection of saline, free drug mixture (4 mg/kg body weight for NLG919 and aPD-L1) or SPINs (0.2 mL, 0.6 mg/mL) via tail vein with or without US irradiation (1.0 MHz, 1.2 W/cm^2, 50% duty cycle, 10 min). The cell nucleus stained by DAPI shows blue fluorescence signal, PD-L1 stained by corresponding antibody shows green fluorescence signal. (**b**) MFI of PD-L1 staining in tumor sections after different treatments (n = 4). Saline + US versus $SPIN_0$ + US: $P < 0.0001$; Saline + US versus $SPIN_N$ + US: $P < 0.0001$; Saline + US versus $SPIN_A$ + US: $P < 0.0001$; Saline + US versus $SPIN_{D1}$ + US: $P < 0.0001$; Saline + US versus $SPIN_{D2}$ + US: $P < 0.0001$. Data are presented as mean values ± SD. Statistical significance was calculated via one-way ANOVA with a Tukey post-hoc test; *** $P < 0.001$. Source data are provided as a Source Data file.

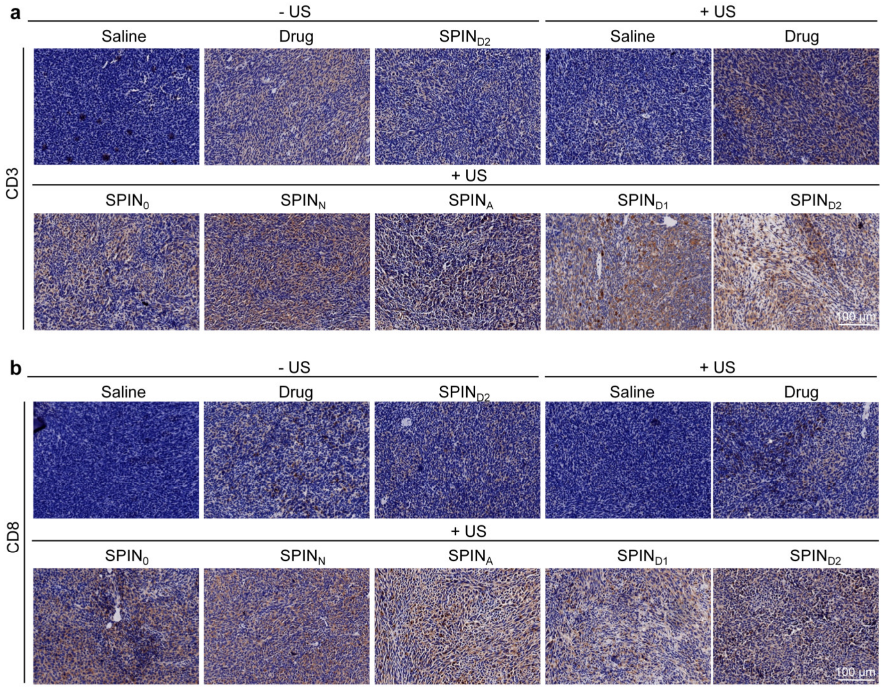

Supplementary Figure 33. Study of sonodynamically induced tumor-infiltrating lymphocytes in tumor tissues. Representative immunohistochemical staining images of CD3$^+$ (**a**) and CD8$^+$ (**b**) tumor-infiltrating lymphocytes in tumor tissues from tumor-bearing mice after different treatments for 3 days. Brown staining represents CD3$^+$ and CD8$^+$ tumor-infiltrating lymphocytes. The experiments in a and b were repeated independently three times with similar results.

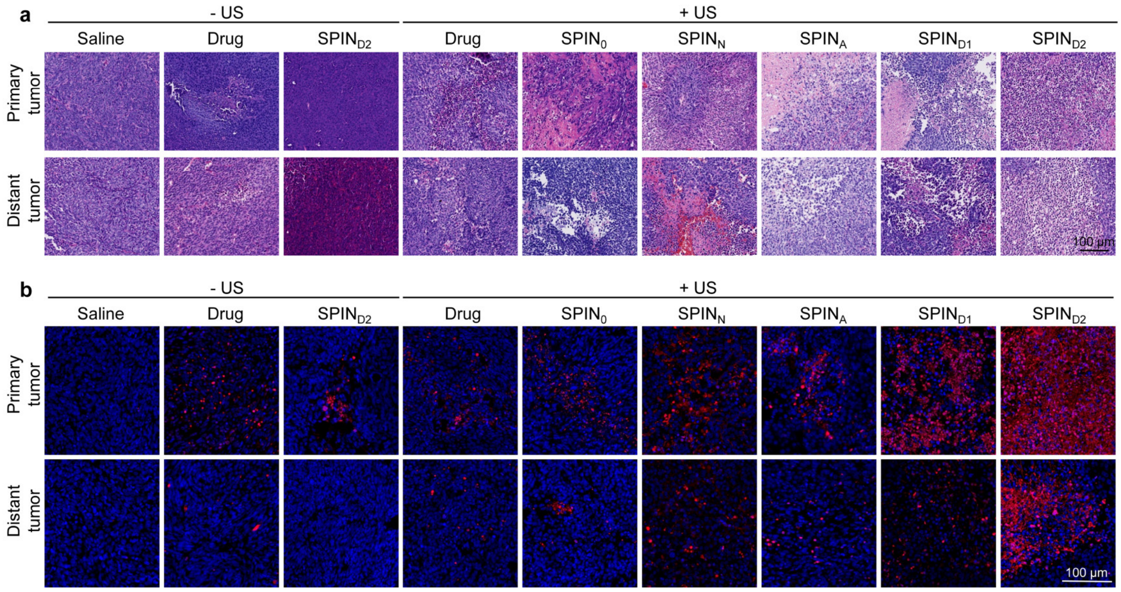

Supplementary Figure 34. Histological analysis of tumors after different treatments. (**a**) H&E staining images of primary and distant tumors of Panc02 tumor-bearing C57BL/6 mice after systemic injection of saline, free drug mixture (4 mg/kg body weight for NLG919 and aPD-L1), or SPINs (0.2 mL, 0.6 mg/mL) with or without US irradiation (1.0 MHz, 1.2 W/cm^2, 50% duty cycle, 10 min). (**b**) TUNEL staining images of primary and distant tumors of mice after different treatments. The cell

nucleus stained by DAPI shows blue fluorescence signal, TUNEL stained by corresponding antibody shows red fluorescence signal. The experiments in a and b were repeated independently three times with similar results.

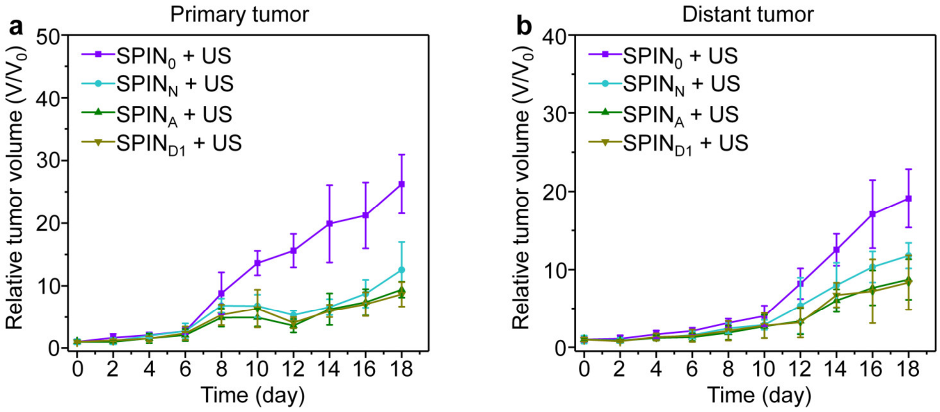

Supplementary Figure 35. Evaluation of tumor growth inhibition efficacy. Relative tumor volumes of primary (**a**) and distant (**b**) tumors of Panc02 tumor-bearing C57BL/6 mice (n = 6) after systemic injection of $SPIN_0$, $SPIN_N$, $SPIN_A$, or $SPIN_{D1}$ (0.2 mL, 0.6 mg/mL) with US irradiation (1.0 MHz, 1.2 W/cm^2, 50% duty cycle, 10 min). Data are presented as mean values ± SD. Source data are provided as a Source Data file.

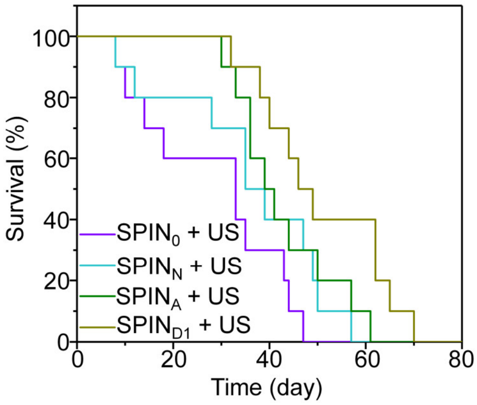

Supplementary Figure 36. Evaluation of survival of mice after different treatments. Survival curves of Panc02 tumor-bearing C57BL/6 mice (n = 10) after systemic injection of $SPIN_0$, $SPIN_N$, $SPIN_A$, or $SPIN_{D1}$ (0.2 mL, 0.6 mg/mL) with US irradiation (1.0 MHz, 1.2 W/cm^2, 50% duty cycle, 10 min). Source data are provided as a Source Data file.

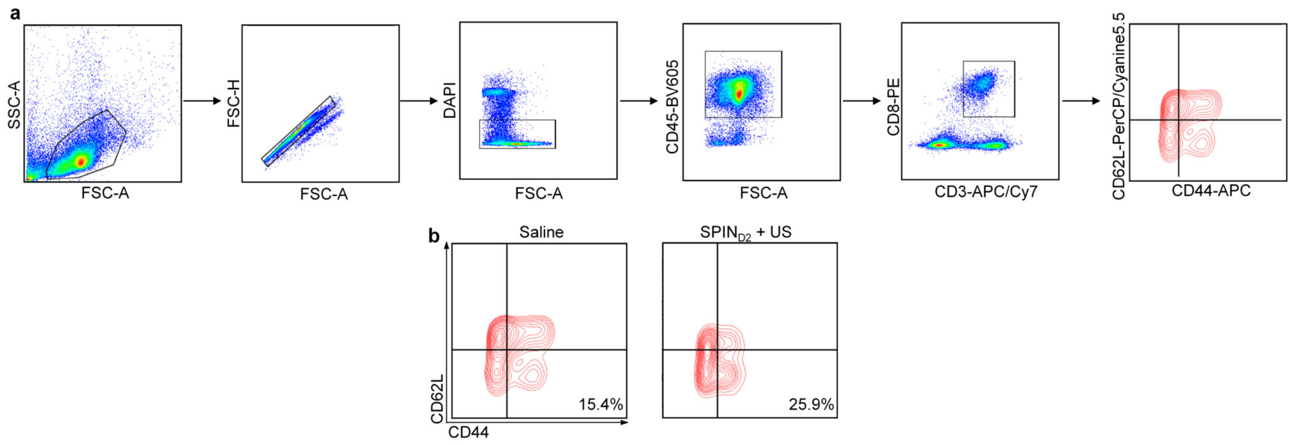

Supplementary Figure 37. Flow cytometry analysis of effector memory T cells. (**a**) Gating strategy for flow cytometry analysis of effector memory T cells. (**b**) Representative flow cytometry plots of effector memory T cells ($CD44^{+}CD62L^{-}$) in spleen of Panc02 tumor-bearing mice after different treatments followed by tumor rechallenge.

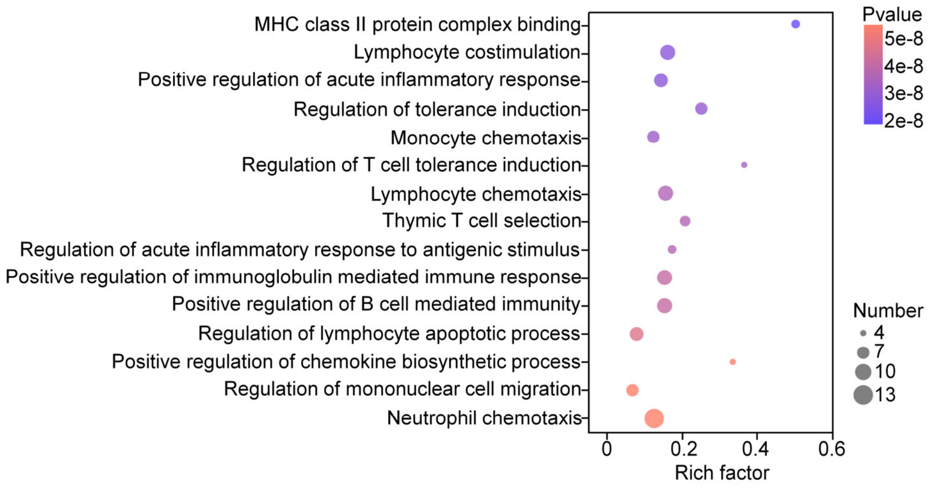

Supplementary Figure 38. Gene expression assay of immune-related genes. GO enrichment analysis of differentially upregulated genes associated with immune processes. Statistical significance was calculated via two-tailed Student's t-test.

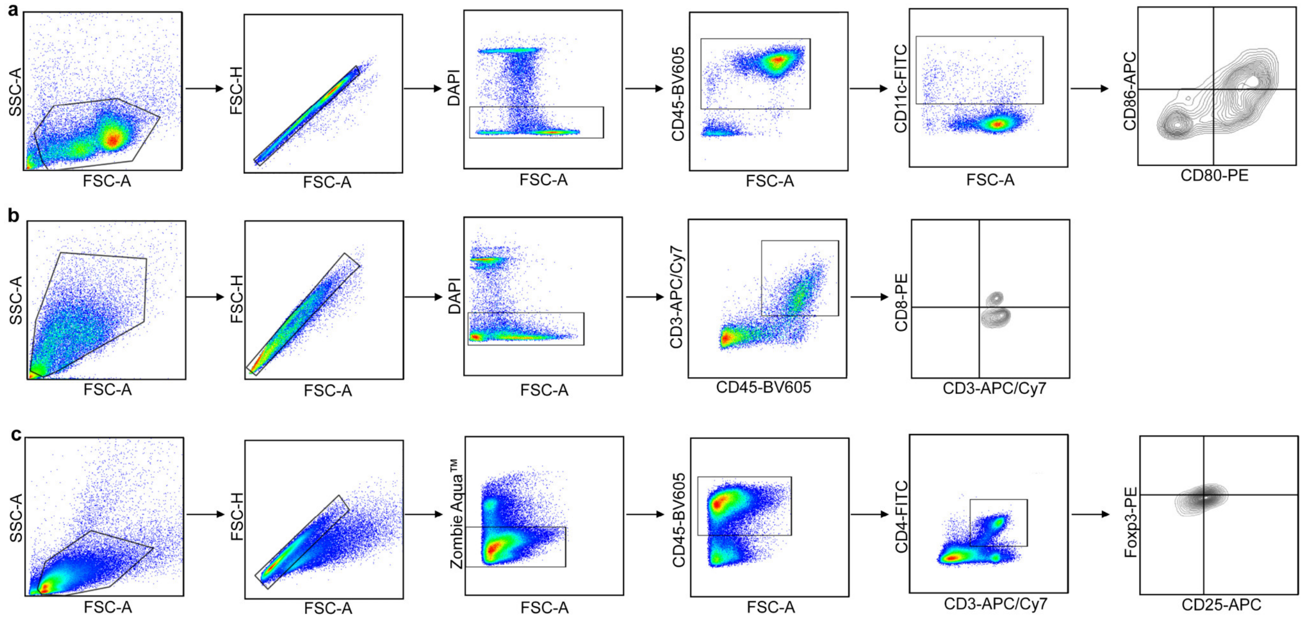

Supplementary Figure 39. Gating strategy for flow cytometry analysis. Gating strategy for flow cytometry analysis of matured DCs (**a**), CD3⁺CD8⁺ CTLs (**b**) and T_{reg} cells (**c**).

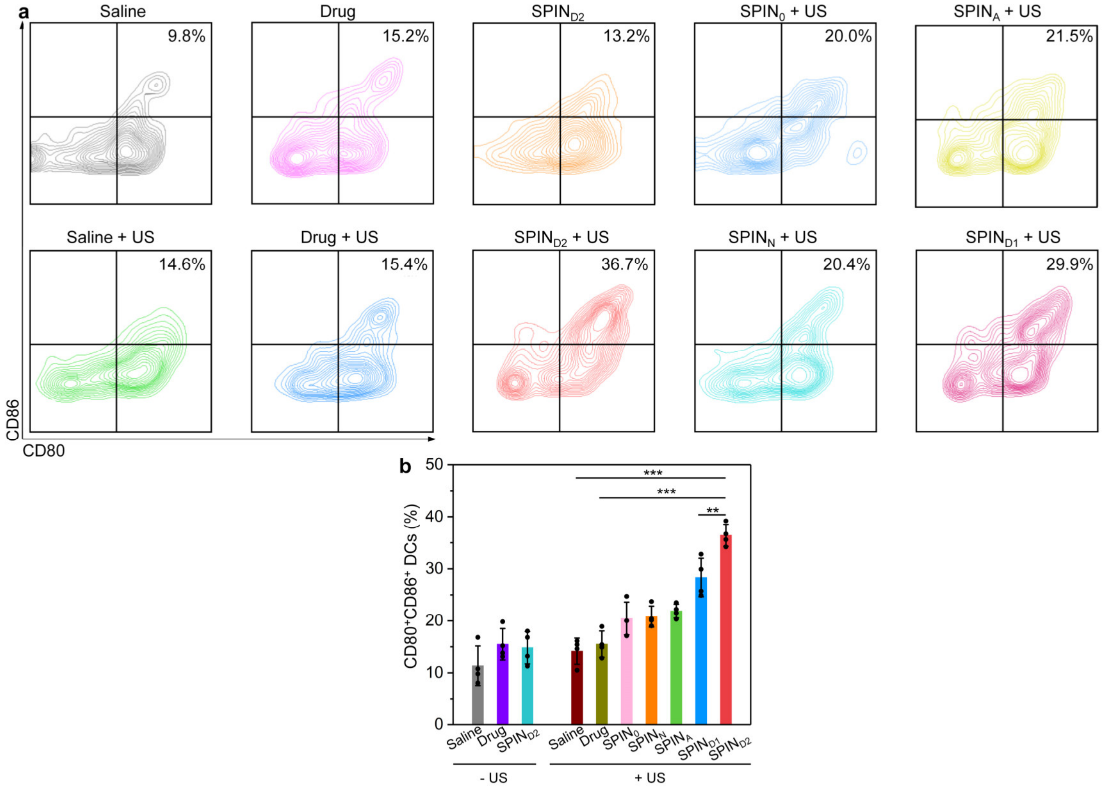

Supplementary Figure 40. Flow cytometry analysis of matured DCs. (**a**) Representative flow cytometry plots of matured DCs ($CD80^+CD86^+$ gating on $CD11c^+$) in tumor-draining lymph nodes of Panc02 tumor-bearing C57BL/6 mice after different treatments. (**b**) Populations of $CD80^+CD86^+$ DCs in tumor-draining lymph nodes of mice after different treatments (n = 4). Saline + US versus $SPIN_{D2}$ + US: $P < 0.0001$; Drug + US versus $SPIN_{D2}$ + US: $P < 0.0001$; $SPIN_{D1}$ + US versus $SPIN_{D2}$ + US: $P = 0.0090$. Data are presented as mean values ± SD. Statistical significance was calculated via two-tailed Student's t-test; **$P<0.01$, ***$P < 0.001$. Source data are provided as a Source Data file.

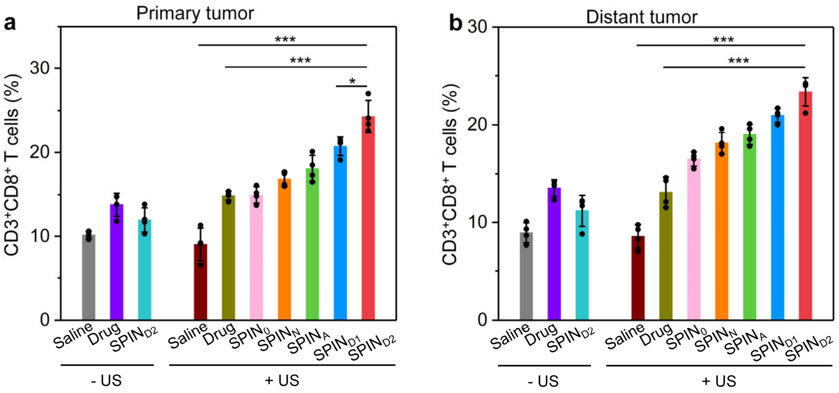

Supplementary Figure 41. Flow cytometry analysis of CTLs. Populations of $CD3^+CD8^+$ T cells in primary tumors (**a**) and distant tumors (**b**) of mice after different treatments (n = 4). Saline + US versus $SPIN_{D2}$ + US: $P < 0.0001$; Drug + US versus $SPIN_{D2}$ + US: $P < 0.0001$; $SPIN_{D1}$ + US versus $SPIN_{D2}$ + US: $P = 0.0183$ for primary tumors (**a**); Saline + US versus $SPIN_{D2}$ + US: $P < 0.0001$; Drug + US versus $SPIN_{D2}$ + US: $P < 0.0001$ for distant tumors (**b**). Data are presented as mean values ± SD. Statistical significance was calculated via two-tailed Student's t-test; *$P<0.05$, ***$P < 0.001$. Source data are provided as a Source Data file.

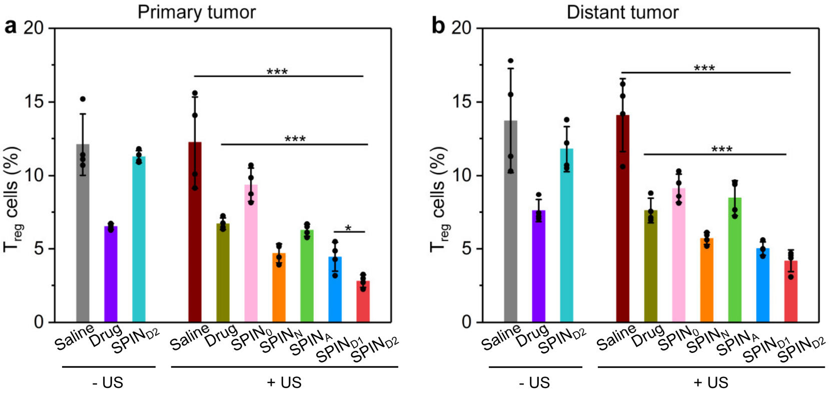

Supplementary Figure 42. Flow cytometry analysis of T_{reg} cells. Populations of T_{reg} cells in primary tumors (**a**) and distant tumors (**b**) of mice after different treatments (n = 4). Saline + US versus $SPIN_{D2}$ + US: $P < 0.0001$; Drug + US versus $SPIN_{D2}$ + US: $P < 0.0001$; $SPIN_{D1}$ + US versus $SPIN_{D2}$ + US: $P = 0.0216$ for primary tumors (**a**); Saline + US versus $SPIN_{D2}$ + US: $P < 0.0001$; Drug + US versus $SPIN_{D2}$ + US: $P < 0.0001$ for distant tumors (**b**). Data are presented as mean values ± SD. Statistical significance was calculated via two-tailed Student's t-test; *$P<0.05$, ***$P < 0.001$. Source data are provided as a Source Data file.

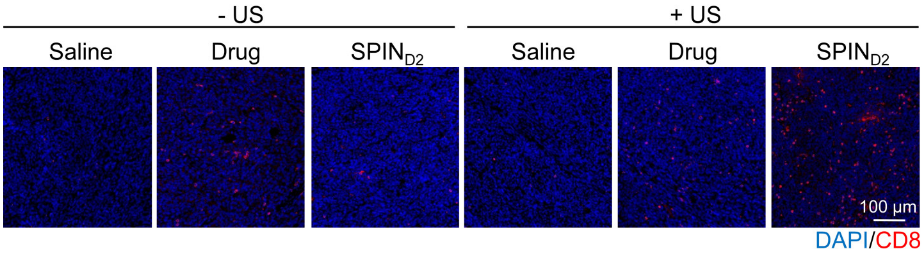

Supplementary Figure 43. Evaluation of CD8^{+} T cells in tumor tissues. Immunofluorescence CD8 staining images of tumor sections from Panc02 tumor-bearing mice after systemic injection of saline, free drug mixture (4 mg/kg body weight for NLG919 and aPD-L1) or SPIN$_{D2}$ (0.2 mL, 0.6 mg/mL) via tail vein with or without US irradiation (1.0 MHz, 1.2 W/cm^2, 50% duty cycle, 10 min). The cell nucleus stained by DAPI shows blue fluorescence signal, CD8 stained by corresponding antibody show red fluorescence signal. The experiments were repeated independently three times with similar results.

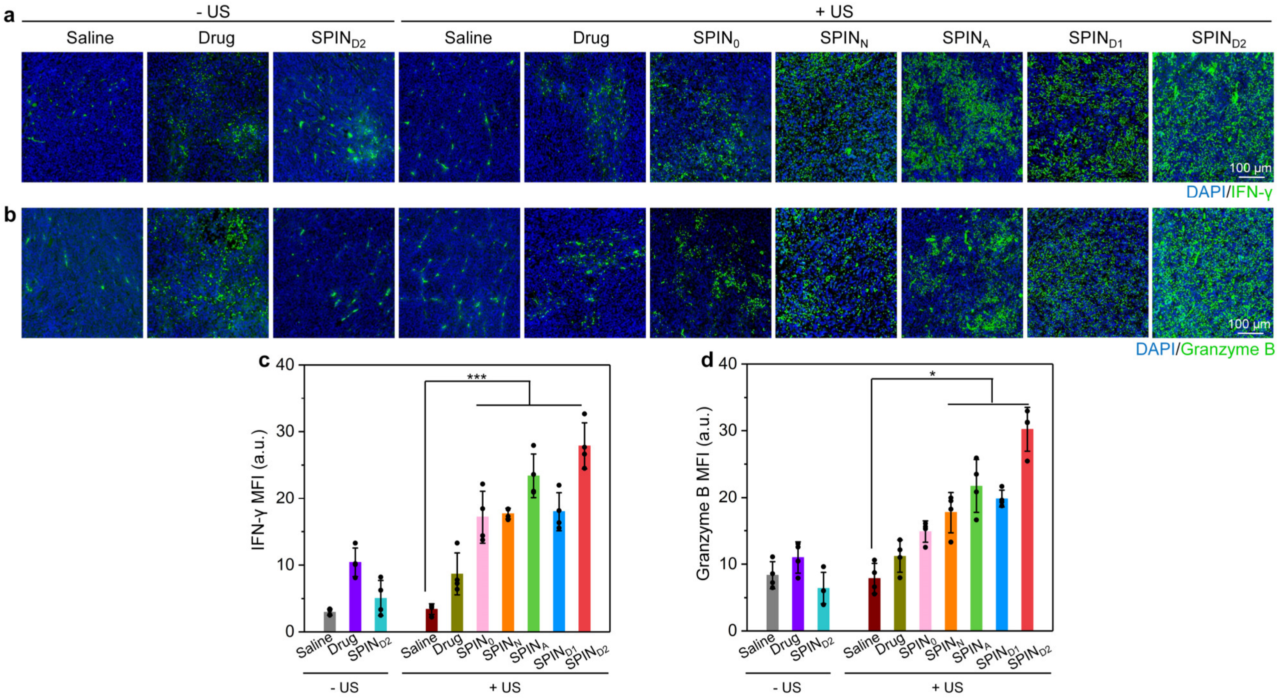

Supplementary Figure 44. Evaluation of IFN-γ and Granzyme B expression levels in tumors. (**a,b**) Immunofluorescence IFN-γ (**a**) and Granzyme B (**b**) staining images of tumor sections from Panc02 tumor-bearing mice after systemic injection of saline, free drug mixture (4 mg/kg body weight for NLG919 and aPD-L1) or SPINs (0.2 mL, 0.6 mg/mL) via tail vein with or without US irradiation (1.0 MHz, 1.2 W/cm^2, 50% duty cycle, 10 min). The cell nucleus stained by DAPI shows blue fluorescence signal, IFN-γ and Granzyme B stained by corresponding antibodies show green fluorescence signal. (**c,d**) MFI of IFN-γ (**c**) and Granzyme B (**d**) staining in tumor sections from different groups (n = 4). Saline + US versus SPIN$_0$ + US: $P < 0.0001$; Saline + US versus SPIN$_N$ + US:

$P < 0.0001$; Saline + US versus $SPIN_A$ + US: $P < 0.0001$; Saline + US versus $SPIN_{D1}$ + US: $P < 0.0001$; Saline + US versus $SPIN_{D2}$ + US: $P < 0.0001$ for IFN-γ (**c**); Saline + US versus $SPIN_N$ + US: $P = 0.0161$; Saline + US versus $SPIN_A$ + US: $P < 0.0001$; Saline + US versus $SPIN_{D1}$ + US: $P = 0.0023$; Saline + US versus $SPIN_{D2}$ + US: $P < 0.0001$ for Granzyme B (**d**). Data are presented as mean values ± SD. Statistical significance was calculated via one-way ANOVA with a Tukey post-hoc test; $^{*}P < 0.05$, $^{***}P < 0.001$. Source data are provided as a Source Data file.

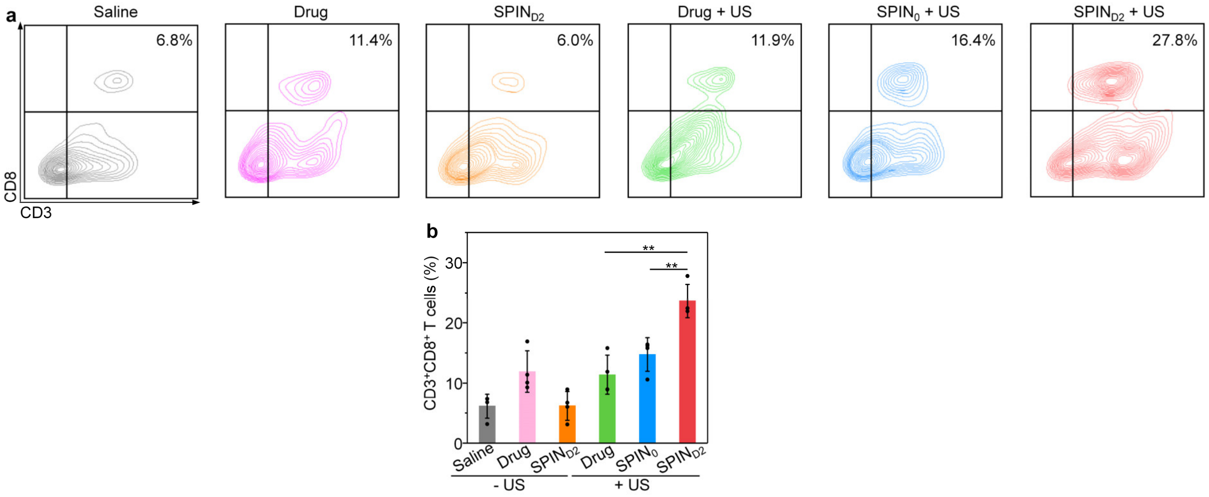

Supplementary Figure 45. Flow cytometry analysis of CTLs after deep-tissue therapy using SPINs. (**a**) Representative flow cytometry plots of CTLs ($CD3^+CD8^+$ gating on $CD45^+$) in 5-cm tissue covered tumors from Panc02 tumor-bearing mice after systemic injection of saline, free drug mixture (4 mg/kg body weight for NLG919 and aPD-L1), $SPIN_0$ or $SPIN_{D2}$ (0.2 mL, 0.6 mg/mL) with or without US irradiation (1.0 MHz, 1.2 W/cm^2, 50% duty cycle, 10 min). (**b**) Populations of $CD3^+CD8^+$ T cells in 5-cm tissue covered tumors of mice after different treatments (n = 4). Drug + US versus $SPIN_{D2}$ + US: $P = 0.0012$; $SPIN_0$ + US versus $SPIN_{D2}$ + US: $P = 0.0041$. Data are presented as mean values ± SD. Statistical significance was calculated via two-tailed Student's t-test; $^{**}P < 0.01$. Source data are provided as a Source Data file.

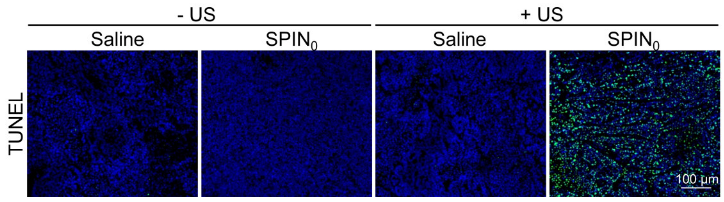

Supplementary Figure 46. Histological analysis of orthotopic pancreatic rabbit tumors after different treatments. Immunofluorescence TUNEL staining images of orthotopic pancreatic rabbit

tumors after different treatments. The cell nucleus stained by DAPI shows blue fluorescence signal, TUNEL stained by corresponding antibody shows green fluorescence signal. The experiments were repeated independently three times with similar results.

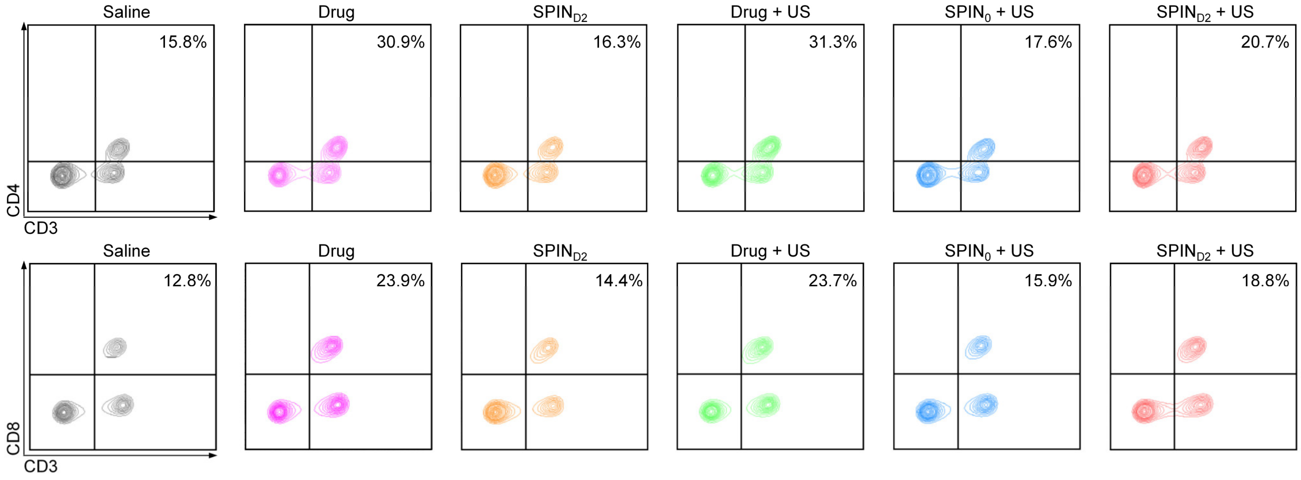

Supplementary Figure 47. Evaluation of T cells in blood. Representative flow cytometry plots of $CD3^+CD4^+$ and $CD3^+CD8^+$ T cells in blood of mice at day 30 after systemic administrations of saline, SPIN$_0$, SPIN$_{D2}$ (0.2 mL, 1.2 mg/mL) or free drug mixture (8 mg/kg body weight for NLG919 and aPD-L1) with or without US irradiation (1.0 MHz, 1.2 W/cm^2, 50% duty cycle, 10 min).

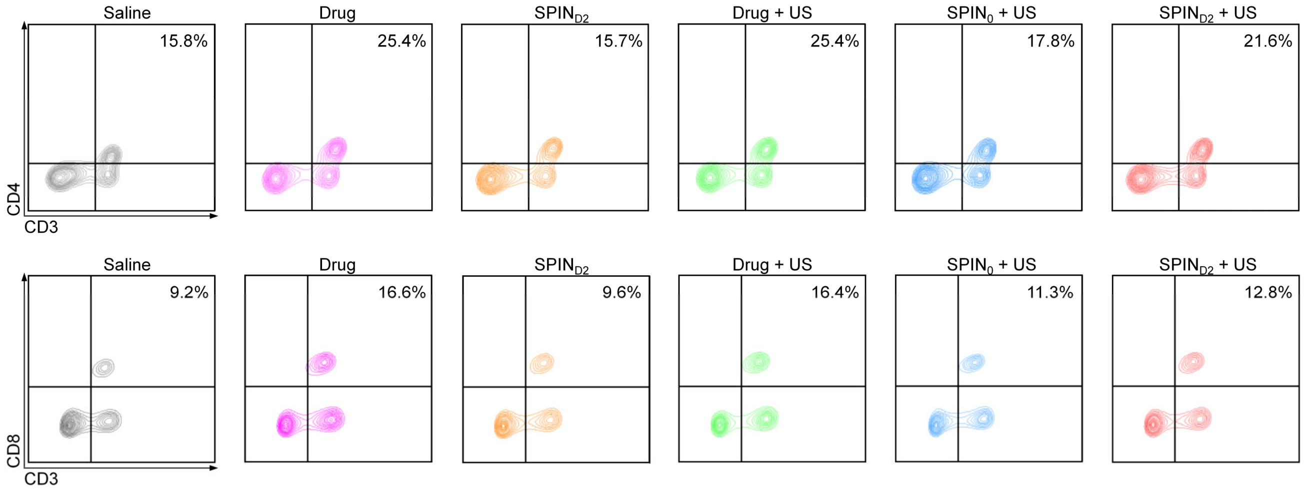

Supplementary Figure 48. Evaluation of T cells in spleen. Representative flow cytometry plots of $CD3^+CD4^+$ and $CD3^+CD8^+$ T cells in spleen of mice at day 30 after systemic administrations of saline, SPIN$_0$, SPIN$_{D2}$ (0.2 mL, 1.2 mg/mL) or free drug mixture (8 mg/kg body weight for NLG919 and aPD-L1) with or without US irradiation (1.0 MHz, 1.2 W/cm^2, 50% duty cycle, 10 min).

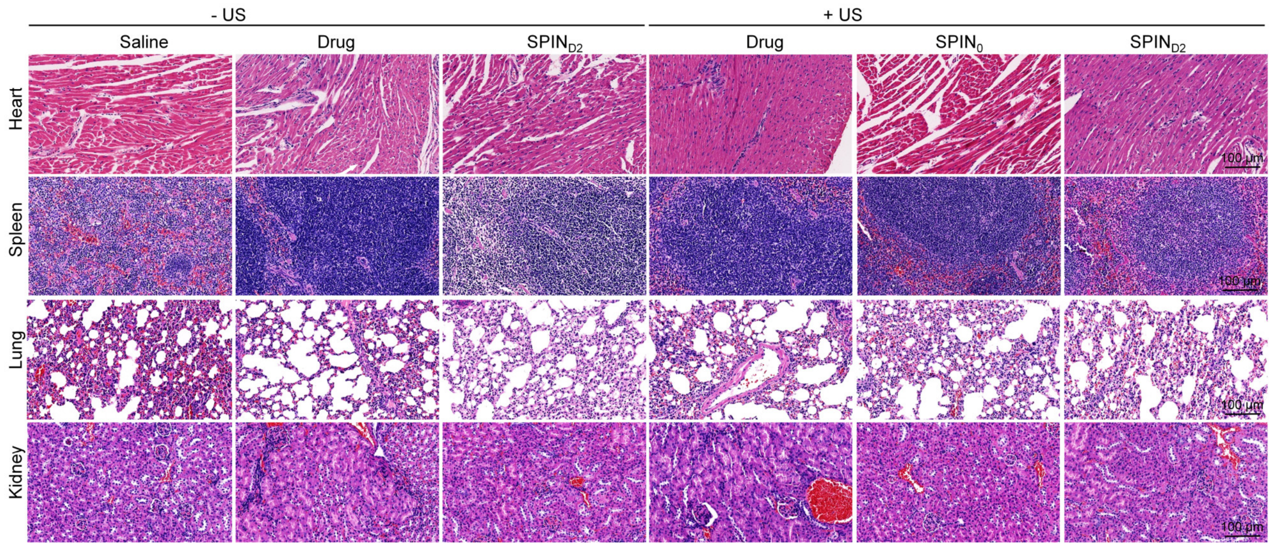

Supplementary Figure 49. In vivo biocompatibility studies by histological analysis. Representative H&E staining images of heart, spleen, lung and kidney of mice at day 30 after systemic administrations of saline, SPIN$_0$, SPIN$_{D2}$ (0.2 mL, 1.2 mg/mL) or free drug mixture (8 mg/kg body weight for NLG919 and aPD-L1) with or without US irradiation (1.0 MHz, 1.2 W/cm^2, 50% duty cycle, 10 min). The experiments were repeated independently three times with similar results.

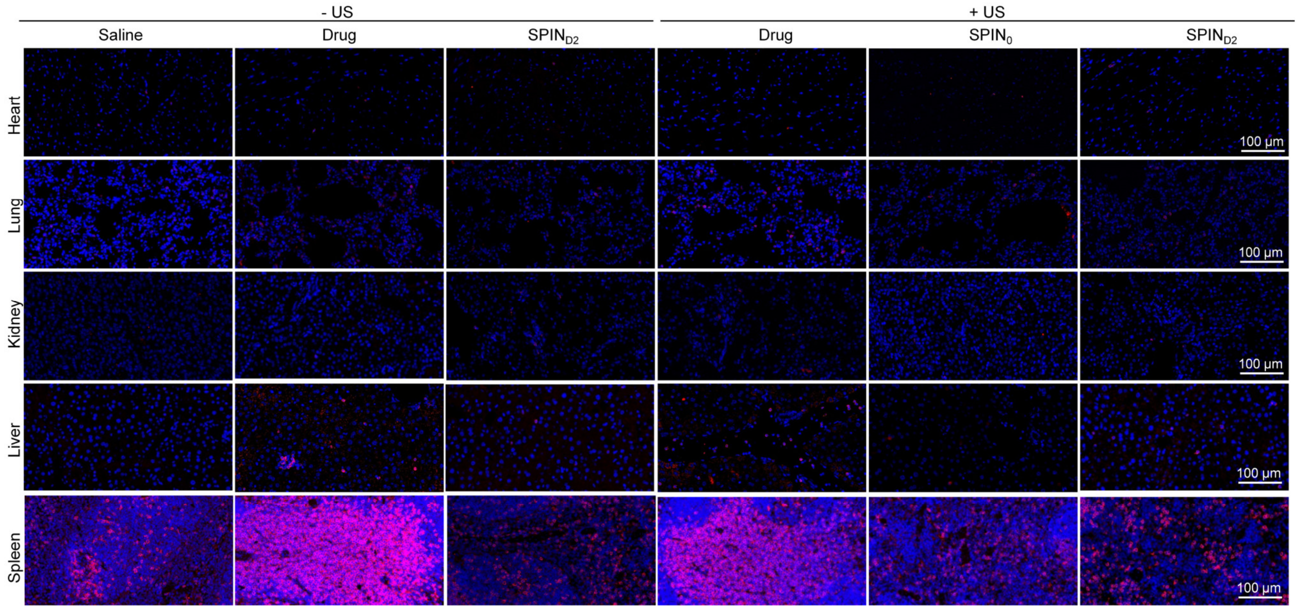

Supplementary Figure 50. CD3$^+$ T cell infiltration studies by immunofluorescence imaging. Representative immunofluorescence CD3 staining images of heart, liver, spleen, lung and kidney of mice at day 30 after systemic administrations of saline, SPIN$_0$, SPIN$_{D2}$ (0.2 mL, 1.2 mg/mL) or free drug mixture (8 mg/kg body weight for NLG919 and aPD-L1) with or without US irradiation (1.0 MHz, 1.2 W/cm^2, 50% duty cycle, 10 min). Cell nucleus stained by DAPI shows blue fluorescence signal; CD3 stained by the corresponding antibody shows red fluorescence signal. The experiments were

repeated independently four times with similar results.

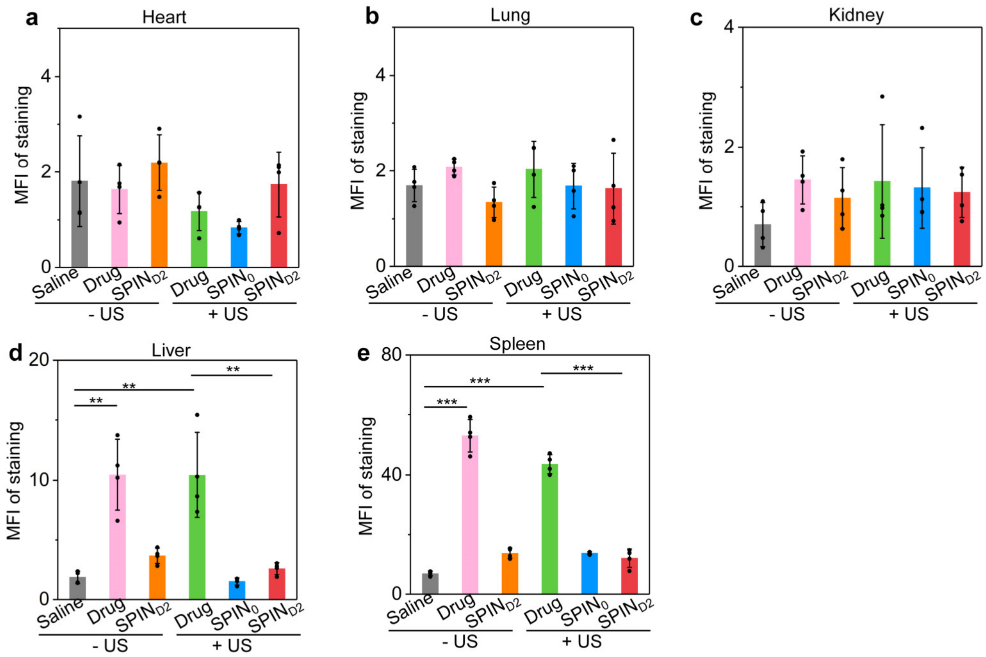

Supplementary Figure 51. CD3⁺ T cell infiltration quantification in major organs. Relative MFI for CD3 staining in heart (**a**), lung (**b**), kidney (**c**), liver (**d**) and spleen (**e**) of mice at day 30 after systemic administrations of saline, SPIN$_0$, SPIN$_{D2}$ (0.2 mL, 1.2 mg/mL) or free drug mixture (8 mg/kg body weight for NLG919 and aPD-L1) with or without US irradiation (1.0 MHz, 1.2 W/cm^2, 50% duty cycle, 10 min) (n = 4). Saline - US versus Drug - US: P = 0.0012; Saline - US versus Drug + US: P = 0.0030; Drug + US versus SPIN$_{D2}$ + US: P = 0.0047 for liver (**d**); Saline - US versus Drug - US: P<0.0001; Saline - US versus Drug + US: P<0.0001; Drug + US versus SPIN$_{D2}$ + US: P<0.0001 for spleen (**e**). Data are presented as mean values ± SD. Statistical significance was calculated via two-tailed Student's t-test; **P<0.01, ***P < 0.001. Source data are provided as a Source Data file.

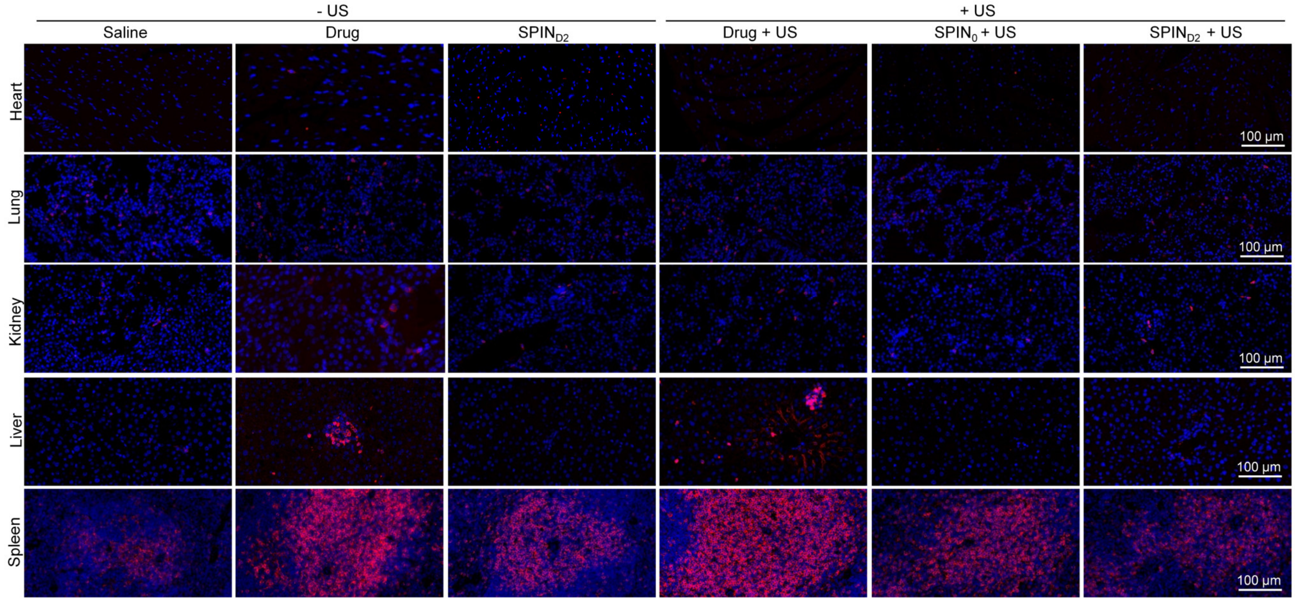

Supplementary Figure 52. C4⁺ T cell infiltration studies by immunofluorescence imaging. Representative immunofluorescence CD4 staining images of heart, liver, spleen, lung and kidney of mice at day 30 after systemic administrations of saline, $SPIN_0$, $SPIN_{D2}$ (0.2 mL, 1.2 mg/mL) or free drug mixture (8 mg/kg body weight for NLG919 and aPD-L1) with or without US irradiation (1.0 MHz, 1.2 W/cm^2, 50% duty cycle, 10 min). Cell nucleus stained by DAPI shows blue fluorescence signal; CD4 stained by the corresponding antibody shows red fluorescence signal. The experiments were repeated independently four times with similar results.

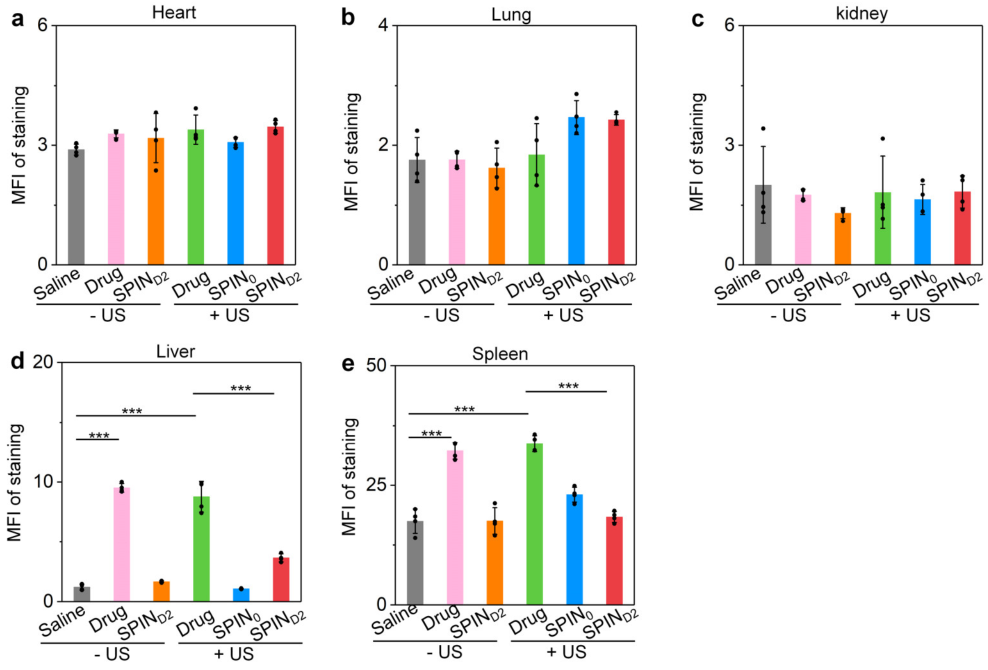

Supplementary Figure 53. CD4⁺ T cell infiltration quantification in major organs. Relative MFI for CD4 staining in heart (**a**), lung (**b**), kidney (**c**), liver (**d**) and spleen (**e**) of mice at day 30 after

systemic administrations of saline, $SPIN_0$, $SPIN_{D2}$ (0.2 mL, 1.2 mg/mL) or free drug mixture (8 mg/kg body weight for NLG919 and aPD-L1) with or without US irradiation (1.0 MHz, 1.2 W/cm^2, 50% duty cycle, 10 min) (n = 4). Saline - US versus Drug - US: P<0.0001; Saline - US versus Drug + US: P<0.0001; Drug + US versus $SPIN_{D2}$ + US: P=0.0002 for liver (**d**); Saline - US versus Drug - US: P<0.0001; Saline - US versus Drug + US: P<0.0001; Drug + US versus $SPIN_{D2}$ + US: P<0.0001 for spleen (**e**). Data are presented as mean values ± SD. Statistical significance was calculated via two-tailed Student's t-test; ***P < 0.001. Source data are provided as a Source Data file.

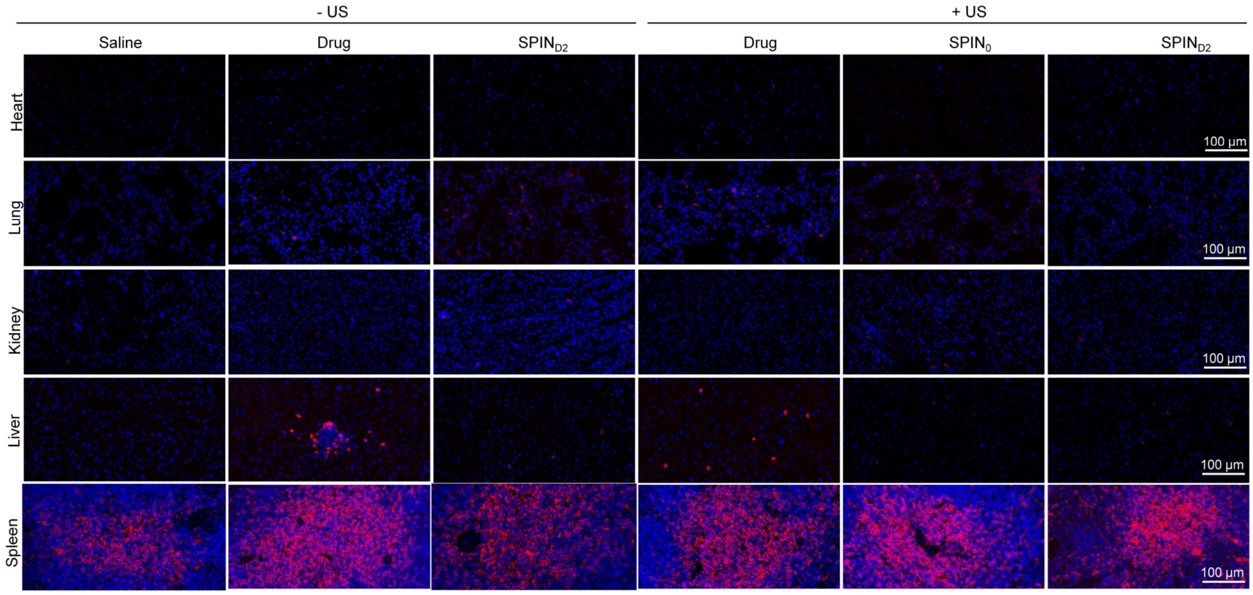

Supplementary Figure 54. C8$^+$ T cell infiltration studies by immunofluorescence imaging. Representative immunofluorescence CD8 staining images of heart, liver, spleen, lung and kidney of mice at day 30 after systemic administrations of saline, $SPIN_0$, $SPIN_{D2}$ (0.2 mL, 1.2 mg/mL) or free drug mixture (8 mg/kg body weight for NLG919 and aPD-L1) with or without US irradiation (1.0 MHz, 1.2 W/cm^2, 50% duty cycle, 10 min). Cell nucleus stained by DAPI shows blue fluorescence signal; CD8 stained by the corresponding antibody shows red fluorescence signal. The experiments were repeated independently four times with similar results.

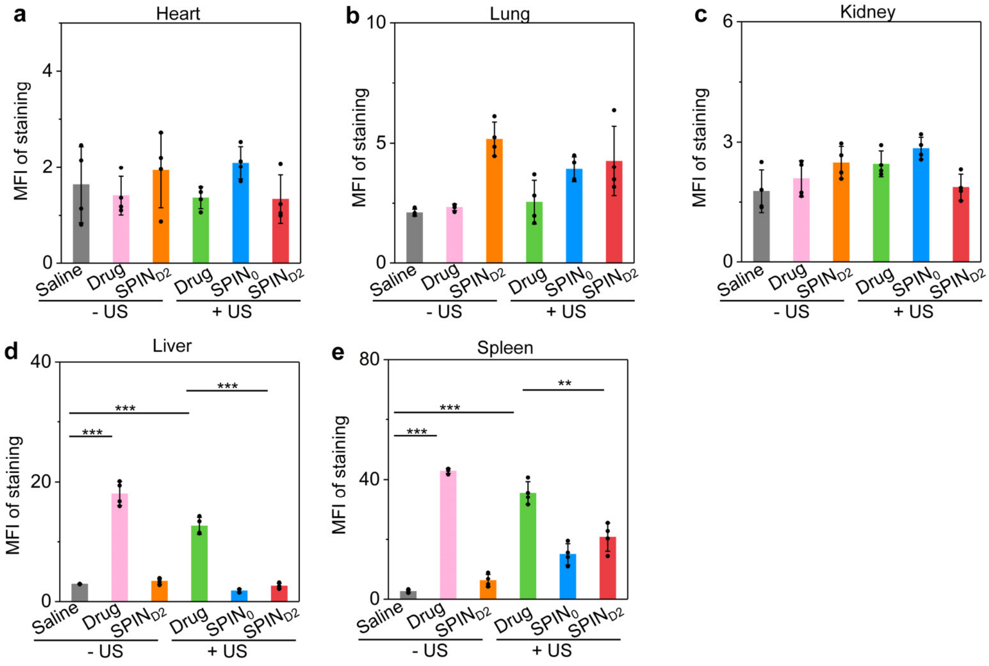

Supplementary Figure 55. $CD8^+$ T cell infiltration quantification in major organs. Relative MFI for CD8 staining in heart (**a**), lung (**b**), kidney (**c**), liver (**d**) and spleen (**e**) of mice at day 30 after systemic administrations of saline, $SPIN_0$, $SPIN_{D2}$ (0.2 mL, 1.2 mg/mL) or free drug mixture (8 mg/kg body weight for NLG919 and aPD-L1) with or without US irradiation (1.0 MHz, 1.2 W/cm^2, 50% duty cycle, 10 min) (n = 4). Saline - US versus Drug - US: P<0.0001; Saline - US versus Drug + US: P<0.0001; Drug + US versus $SPIN_{D2}$ + US: P<0.0001 for liver (**d**); Saline - US versus Drug - US: P<0.0001; Saline - US versus Drug + US: P<0.0001; Drug + US versus $SPIN_{D2}$ + US: P=0.0028 for spleen (**e**). Data are presented as mean values ± SD. Statistical significance was calculated via two-tailed Student's t-test; **P < 0.01, ***P < 0.001. Source data are provided as a Source Data file.

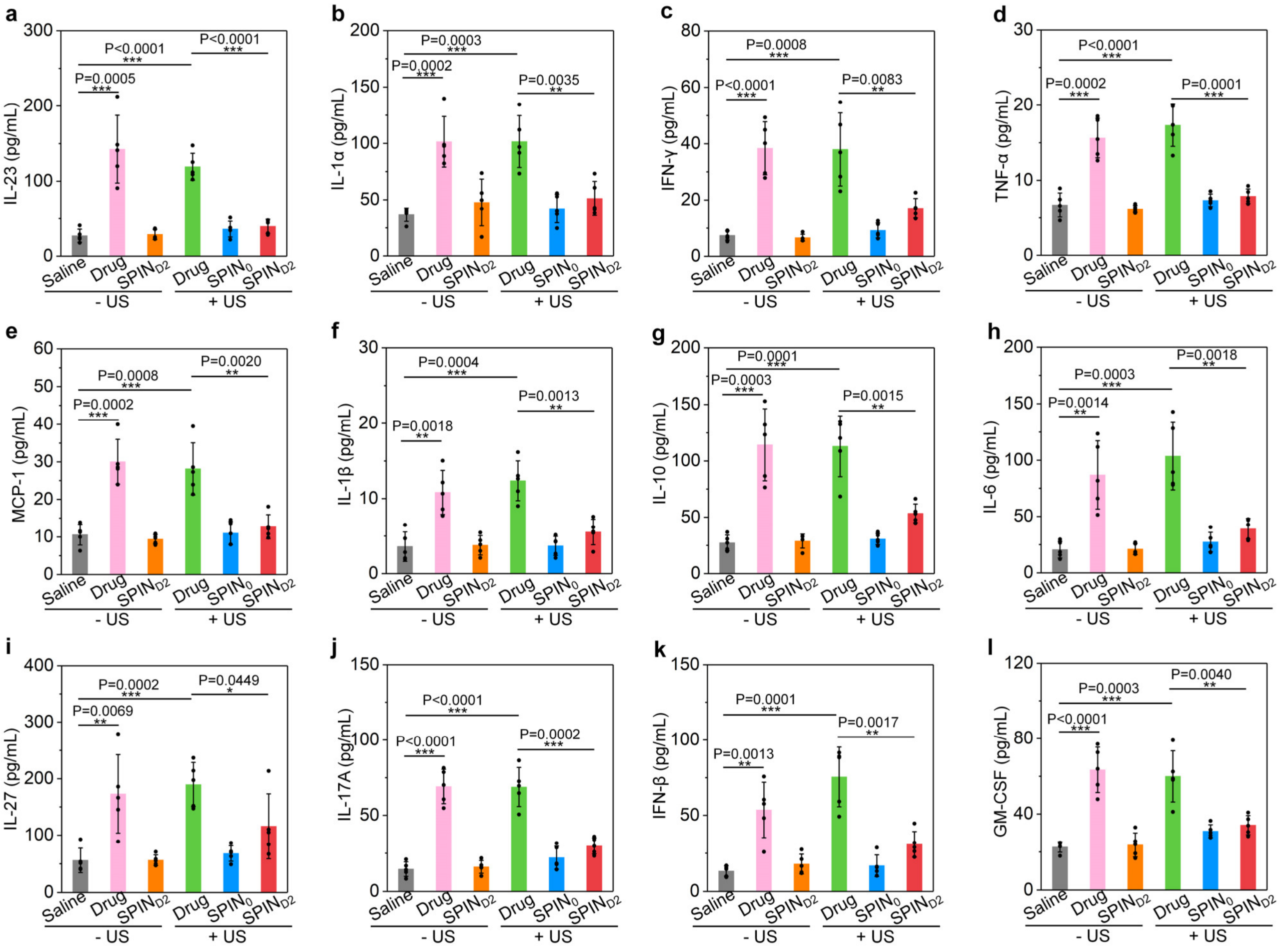

Supplementary Figure 56. Evaluation of cytokine levels in serum. The levels of IL-23 (**a**), IL-1α (**b**), IFN-γ (**c**), TNF-α (**d**), MCP-1 (**e**), IL-1β (**f**), IL-10 (**g**), IL-6 (**h**), IL-27 (**i**), IL-17A (**j**), IFN-β (**k**) and GM-CSF (**l**) in serum of mice at day 30 after systemic administrations of saline, $SPIN_0$, $SPIN_{D2}$ (0.2 mL, 1.2 mg/mL) or free drug mixture (8 mg/kg body weight for NLG919 and aPD-L1) with or without US irradiation (1.0 MHz, 1.2 W/cm^2, 50% duty cycle, 10 min) (n = 5). Data are presented as mean values ± SD. Statistical significance was calculated via two-tailed Student's t-test; *P < 0.05, **P < 0.01, ***P < 0.001. Source data are provided as a Source Data file.

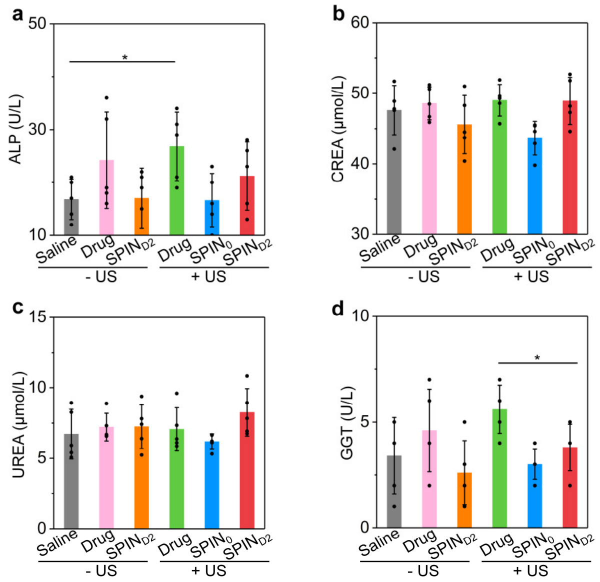

Supplementary Figure 57. Blood biochemical analysis. The levels of ALP (**a**), CREA (**b**), UREA (**c**), and GGT (**d**) in serum of mice at day 30 after systemic administrations of saline, SPIN$_0$, SPIN$_{D2}$ (0.2 mL, 1.2 mg/mL) or free drug mixture (8 mg/kg body weight for NLG919 and aPD-L1) with or without US irradiation (1.0 MHz, 1.2 W/cm^2, 50% duty cycle, 10 min) (n = 5). Saline - US versus Drug + US: P=0.0180 for ALP (**a**); Drug + US versus SPIN$_{D2}$ + US: P=0.0344 for GGT (**d**). Data are presented as mean values ± SD. Statistical significance was calculated via two-tailed Student's t-test; *P < 0.05. Source data are provided as a Source Data file.

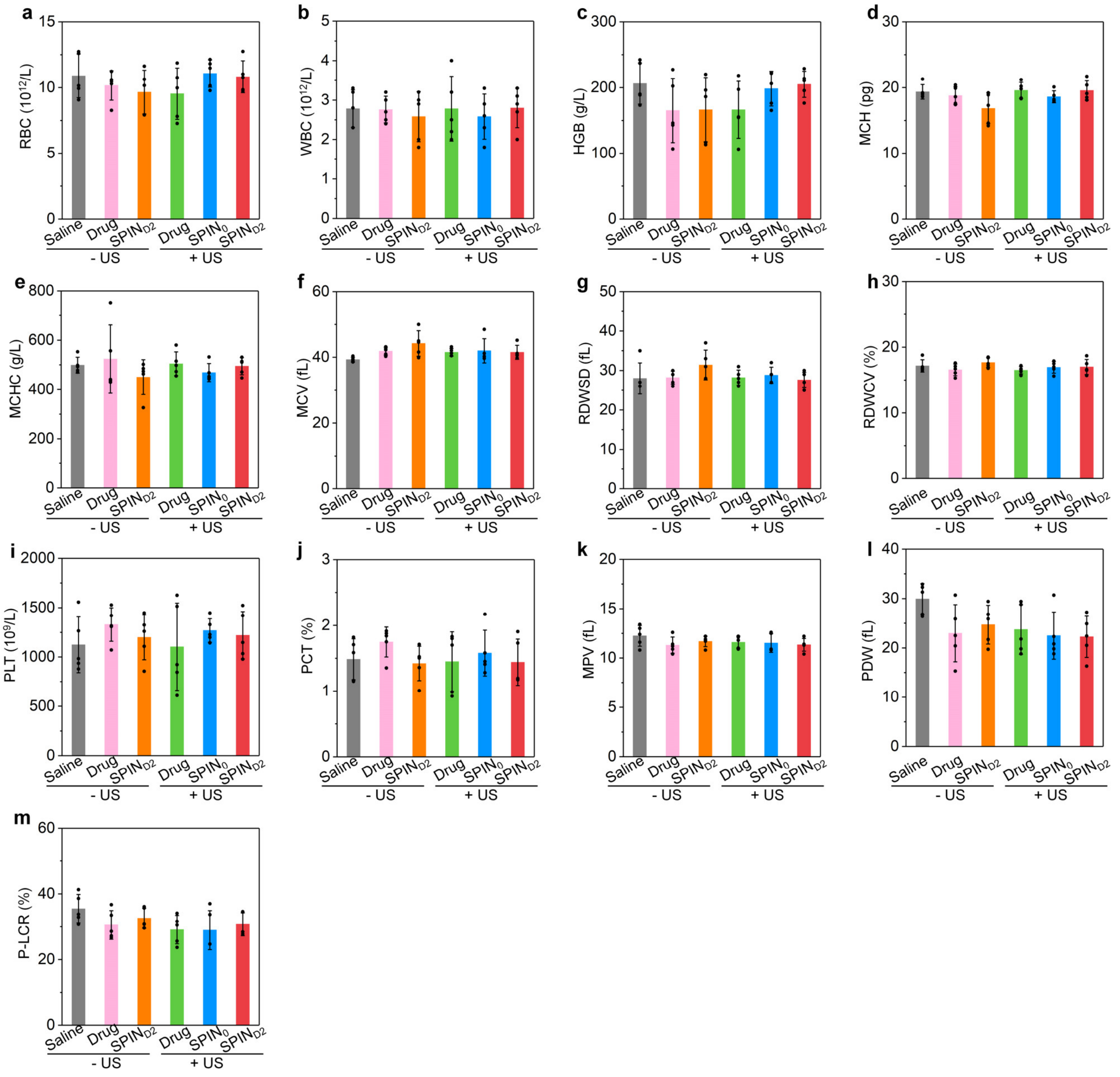

Supplementary Figure 58. Blood routine analysis. Blood routine analysis of RBC (**a**), WBC (**b**), HGB (**c**), MCH (**d**), MCHC (**e**), MCV (**f**), RDWSD (**g**), RDWCV (**h**), PLT (**i**), PCT (**j**), MPV (**k**), PDW (**l**) P-LCR (**m**) in serum of mice at day 30 after systemic administrations of saline, SPIN$_0$, SPIN$_{D2}$ (0.2 mL, 1.2 mg/mL) or free drug mixture (8 mg/kg body weight for NLG919 and aPD-L1) with or without US irradiation (1.0 MHz, 1.2 W/cm^2, 50% duty cycle, 10 min) (n = 5). Data are presented as mean values ± SD. Source data are provided as a Source Data file.

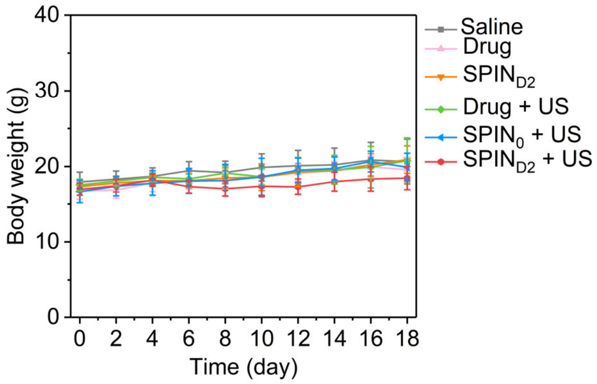

Supplementary Figure 59. Evaluation of mouse body weight change. Body weights of mice after systemic administrations of saline, SPIN$_0$, SPIN$_{D2}$ (0.2 mL, 1.2 mg/mL) or free drug mixture (8 mg/kg body weight for NLG919 and aPD-L1) with or without US irradiation (1.0 MHz, 1.2 W/cm^2, 50% duty cycle, 10 min) (n = 5). Data are presented as mean values ± SD. Source data are provided as a Source Data file.

Supplementary Table 1. Summary of nanomaterials for in vivo sonodynamic therapy of tumors.

Tumor models and mouse strains	Combinational therapy	Material type	US irradiation conditions	Reference
Subcutaneous C32 tumor, Balb/c athymic nude mice	No	TiO_2 nanoparticles	1 MHz, 1 W/cm^2, 50% duty cycle, 2 min	1
Subcutaneous SCC7 tumor, C3H/HeN mice.	No	Au-TiO_2 nanocomposites	1.5 MHz, 30 W, 10% duty cycle, 30 s	2
Subcutaneous 4T1 tumor, BALB/c nude mice	No	PpIX and manganese ions loaded silica nanoparticles	1 MHz, 2.3 W/cm^2, 50% duty cycle, 5 min	3
Subcutaneous PANC-1 tumor, nude mice	No	IR780/fluorocarbon modified silica nanoparticles	1 MHz, 1 W/cm^2, 100% duty cycle, 3 min	4
Subcutaneous U87 tumor, BALB/c nude mice	No	Protoporphyrin/MnO_x silica nanoparticles	1 MHz, 1.5 W/cm^2, 50% duty cycle, 3 min	5
Subcutaneous 4T1 tumor, BALB/c mice	No	Porphyrin contained metal-organic framework	1 MHz, 2.5 W/cm^2, 50% duty cycle, 5 min	6
Subcutaneous MCF-7 tumor, nude mice	No	Methylphenylporphyrin based human serum albumin (HSA)	1 MHz, 2 W/cm^2, 50% duty cycle, 5 min	7
Subcutaneous 4T1 tumor, BALB/c mice	No	Bimetallic oxide $MnWO_X$ nanoparticles	40 kHz, 3 W/cm^2, 50% duty cycle, 5 min	8
Subcutaneous MCF-7 tumor, BALB/c nude mice	No	Au/TiO_2 nanosheets	1 MHz, 1.5 W/cm^2, 5 min	9
Subcutaneous C6 tumor, BALB/c nude mice	No	PpIX modified MnO_2 nanoparticles	1 MHz, 1.5 W/cm^2, 50% duty cycle, 3 min	10
Subcutaneous CT26 tumor, BALB/c nude mice	No	Mn(III)-hemoporfin frameworks	1 MHz, 2.5 W/cm^2, 50% duty cycle, 10 min	11
Subcutaneous 4T1 tumor, BALB/c nude mice	No	Carbon dot/MXene heterojunctions	50 kHz, 3 W/cm^2, 5 min	12

Subcutaneous 4T1 tumor, BALB/c nude mice	No	Iridic-porphyrin complex	3 MHz, 0.3 W/cm^2, 20 min	13
Subcutaneous LNCaP tumor, BALB/c SCID mice	No	Hematoporphyrin loaded poly(L-glutamic acid-L-tyrosine) nanoparticles	1 MHz, 3.5 W/cm^2, 30% duty cycle, 3.5 min	14
Subcutaneous U87MG tumor, BALB/c nude mice	No	IR780 and MnO_2 encapsulated PLGA nanoparticles	3 W/cm^2, 50% duty cycle, 5 min	15
Subcutaneous MDA-MB-231 tumor, BALB/c nude mice	No	N-doped graphene quantum dots	1 MHz, 2.5 W/cm^2, 50% duty cycle, 5 min	16
Subcutaneous B16 tumor, C57BL/6 mice	No	δ-Aminolevulinic acid loaded manganese ferrite nanoparticles	1 MHz, 1.5 W/cm^2, 50% duty cycle	17
Patient-derived tumor xenograft, BALB/c nude mice	No	Patient-derived MVs/AIEgen hybrid system	1 MHz, 0.75 W/cm^2, 30% duty cycle, 10 min	18
Subcutaneous 4T1 tumor, BALB/c mice	No	Hemoglobin-based metalloporphyrin	1 MHz, 1.5 W/cm^2, 50% duty cycle, 5 min	19
Subcutaneous 4T1 tumor, BALB/c mice	No	Defect-rich Ti-based metal–organic framework	1 MHz, 1.5 W/cm^2, 50% duty cycle, 5 min	20
Subcutaneous 4T1 tumor, BALB/c mice	No	Pt-based branched vanadium tetrasulfide nanodendrites	1 MHz, 1.5 W/cm^2, 50% duty cycle, 5 min	21
Subcutaneous BxPC-3 tumor, BALB/c nude mice	Starvation therapy	Erythrocyte membrane camouflaged metal−organic framework integrated with platinum nanoparticles and glucose oxidase	3 MHz, 1.5 W/cm^2, 10 min	22
Orthotopic BxPC-3 tumor, Balb/c SCID mice	Chemotherapy	Rose bengal and 5-fluorouracil functionalized magnetic microbubbles	1 MHz, 3.5 W/cm^2, 30% duty cycle, 3.5 min	23
Subcutaneous SMMC-7721 tumor, BALB/c nude mice	Chemotherapy	Doxorubicin loaded and PpIX conjugated silica nanoparticles	1 MHz, 1.5 W/cm^2, 50% duty cycle, 5 min	24
Subcutaneous MDA-MB-231 tumor, BALB/c nude mice	Chemotherapy	Doxorubicin loaded TiO_2 nanoparticles	1.5 MHz, 15 W/cm^2, 20% duty cycle, 5 min	25
Subcutaneous 4T1 tumor, BALB/c mice	Chemotherapy	Doxorubicin loaded Pt/TiO_2 nanoparticles	1 MHz, 1.5 W/cm^2, 50% duty cycle, 5 min	26
Subcutaneous BxPC-3 tumour, SCID mice	Chemotherapy	Rose bengal and gemcitabine functionalized magnetic microbubbles	1.17 MHz, 30% duty cycle, 3.5 min	27
Subcutaneous HeLa tumor, Nu/Nu nude mice	Chemodynamic therapy	H_2O_2/Fe_3O_4-PLGA polymersomes	40 MHz, 30 min	28
Subcutaneous Saos-2 tumor, BALB/c nude mice	Chemodynamic therapy	Ferrate(VI) and PpIX loaded silica nanoplatforms	1 MHz, 1.4 W/cm^2, 5 min	29
Orthotopic 97H tumor, BALB/c nude mice	Chemodynamic therapy	Gold/manganese oxide hybrid nanoparticles	1 MHz, 2 W/cm^2, 10 min	30
Subcutaneous 4T1 tumor, BALB/c mice	Chemodynamic therapy	$PtCu_3$ nanocages	35 kHz, 3 W/cm^2, 1 min per cycle for ten cycles, 10 min	31
Subcutaneous 4T1 tumor, BALB/c nude mice	Chemodynamic therapy	TiO_2-Fe_3O_4@PEG Janus nanostructure	1 MHz, 1.5 W/cm^2, 50% duty cycle, 5 min	32
Subcutaneous 4T1 and H22 tumors, BALB/c mice	Ferroptosis	Manganese porphyrin-based metal-organic framework	1 MHz, 1 W/cm^2, 50% duty cycle, 5-10 min	33
Subcutaneous RIF-1 tumor, C3H/HeN mice	Photodynamic therapy	Hematoporphyrin and indocyanine green loaded PLGA nanoparticles	1 MHz, 3.5 W/cm^2, 50% duty cycle, 3.5 min	34
Subcutaneous 4T1 tumor, BALB/c nude	Photothermal therapy	Graphene-integrated TiO_2 nanoparticles	1 MHz, 1 W/cm^2, 50% duty cycle, 5 min	35

mice				
Subcutaneous 4T1 tumor, nude mice	Photothermal therapy	TiO_{2-x} layer coated TiO_2 nanocrystals	1 MHz, 1.5 W/cm^2, 50% duty cycle, 5 min	36
Subcutaneous CT26 tumor, BALB/c nude mice	Photothermal therapy	Tetra-(4-aminophenyl) porphyrin loaded Pt-CuS nanoparticles	1 MHz, 1 W/cm^2, 60% duty cycle, 5 min	37
Subcutaneous 4T1 tumor, BALB/c mice	Photothermal therapy, chemotherapy	Polypyrrole coated and honokiol loaded TiO_2 nanoparticles	1 MHz, 1.5 W/cm^2, 1 min	38
Subcutaneous MDA-MB-231 tumor, nude mice	Photothermal and photodynamic therapy	Peptide-ICG nanomicelles	1 MHz, 2.4 W/cm^2, 50% duty cycle, 5 min	39
Subcutaneous 4T1 tumor, BALB/c mice	Photothermal therapy	Polypeptide-capped Te nanorods	1 MHz, 1 W/cm^2, 2 min	40
Subcutaneous 4T1 tumor, BALB/c mice	Photothermal therapy	Titanium carbide nanosheets	40 kHz, 3 W/cm^2, 1 min per cycle, 15 cycles	41
Subcutaneous HeLa tumor, BALB/c nude mice	Photothermal therapy	Cancer cell membrane camouflaged iridium complexes functionalized black-titanium nanoparticles	1 MHz, 1 W/cm^2, 50% duty cycle, 5 min	42
Subcutaneous 4T1 tumor, BALB/c mice	Photothermal therapy	Titanium nitride nanodots	40 kHz, 3.0 W/cm^2, 50% duty cycle, 10 min	43
Orthotopic 4T1 tumor, BALB/c mice	Immunotherapy (TLR7 agonist and aPD-L1)	Hematoporphyrin loaded liposomes	1 MHz, 1.5 W/cm^2, 50% duty cycle, 5 min	44
Subcutaneous 4T1 tumor, BALB/c mice	Immunotherapy (NLG919), gas therapy	Gold-black phosphorus quantum dots-doped mesoporous silica nanoframeworks	1 MHz, 1 W/cm^2, 3 min	45
Subcutaneous B16F10 tumor, BALB/c mice	Immunotherapy (aPD-L1)	Melanoma cell membrane-coated TiO_2 nanoparticles modified with aPD-L1	1 MHz, 3 W/cm^2, 20% duty cycle, 5 min	46
Subcutaneous 4T1 tumor, BALB/c mice	Immunotherapy (immunogenic cell death)	Protoporphyrin-modified mesoporous organosilica nanoparticles	1 MHz, 1 W/cm^2, 50% duty cycle, 5 min	47
Subcutaneous CT26 tumor, BALB/c mice	Immunotherapy (TLR9 agonist)	Zn^{2+}-tetrakis(4-carboxyphenyl) porphyrin nanosheets	40 kHz, 2 W/cm^2, 30 min.	48
Subcutaneous hepa1-6 tumor, C57BL/6 mice	Immunotherapy (CpG and aPD-L1)	Titanium dioxide-Chlorin e6-CpG nanosonosensitizers	1 MHz, 2 W/cm^2, 50% duty cycle, 7 min	49
Subcutaneous U14 tumor, BALB/c mice	Immunotherapy (aPD-L1), gas therapy	L-arginine (LA)-loaded black mesoporous titania nanosystems	1 MHz, 1.5 W/cm^2, 50% duty cycle, 5 min	50
Subcutaneous 4T1 tumor, BALB/c mice	Gas therapy, Immunotherapy (M1 macrophages)	Perfluorodecalin and IR780 encapsulated human serum albumin-based NO donor	1 MHz, 1 W/cm^2, 5 min	51
Subcutaneous 4T1 tumor, BALB/c mice	Immunotherapy (M1 macrophages)	Manganese protoporphyrin liposomes	1 MHz, 2 W/cm^2, 50% duty cycle, 5 min	52
Subcutaneous 4T1 tumor, BALB/c mice	Immunotherapy (aPD-L1)	Titanium dioxide@CaP nanoparticles	3 MHz, 2.1 W, 20 min	53
Subcutaneous B16-OVA tumor, C57BL/6 mice	Immunotherapy (CpG and aPD-L1)	CpG loaded manganese porphyrin-based metal-organic frameworks	1 MHz, 1 W/cm^2, 50% duty cycle, 10 min	54
Subcutaneous B16-F10 tumor, C57BL/6 mice	Immunotherapy (aPD-L1)	Chlorin e6@aPD-L1 lipids	2 MHz, 2 W/cm^2, 20% duty cycle, 10 min	55
Subcutaneous 4T1 tumor, BALB/c mice	Immunotherapy (aPD-L1)	$CoFe_2O_4$ Nanoflowers	1 MHz, 1 W/cm^2, 20% duty cycle, 5 min	56

Supplementary Table 2. Summary of injection dosage of NLG919 and aPD-L1 for combinational immunotherapy of different tumor models.

Immunotherapeutic modulator	Dosage (μg/mouse)	Combinational therapy	Tumor models and mouse strains	Injection way	Reference

NLG919	2500	Chemotherapy (paclitaxel)	Subcutaneous 4T1 tumor, BALB/c mice	i.v. injection	57
NLG919	2100	Chemotherapy (doxorubicin)	Subcutaneous 4T1 tumor, BALB/c mice	i.v. injection	58
NLG919	2000	Chemotherapy (paclitaxel)	Subcutaneous B16-F10 tumor, C57BL/6 mice	intragastric administration	59
NLG919	2500	Chemotherapy (oxaliplatin)	Subcutaneous 4T1 tumor, BALB/c mice	i.v. injection	60
NLG919	2500	Chemotherapy (paclitaxel)	Subcutaneous 4T1 tumor, BALB/c mice	i.v. injection	61
NLG919	2000	Chemotherapy (doxorubicin)	Subcutaneous 4T1 tumor, BALB/c mice	Oral administration	62
NLG919	2100	Chemotherapy (docetaxel)	Subcutaneous 4T1 tumor, BALB/c mice	i.v. injection	63
NLG919	2400	Chemotherapy (gemcitabine and paclitaxel)	Subcutaneous Panc02 tumor, C57BL/6 mice	i.v. injection	64
NLG919	480	Chemotherapy (curcumin)	Subcutaneous B16F10 tumor, C57BL/6 mice	i.v. injection	65
NLG919	400	Chemotherapy (oxaliplatin)	Subcutaneous and orthotopic CT26 tumor, C57BL/6 mice	i.v. injection	66
NLG919	2000	Chemotherapy (doxorubicin)	Subcutaneous 4T1 tumor, BALB/c mice	i.v. injection	67
NLG919	300	Chemotherapy (chlorambucil)	Subcutaneous 4T1 tumor, BALB/c mice	i.v. injection	68
NLG919	900	Chemotherapy (paclitaxel)	Subcutaneous B16F10 tumor, C57BL/6 mice	i.v. injection	69
NLG919	1500	Chemotherapy (doxorubicin)	Subcutaneous CT26 and orthotopic 4T1 tumor, BALB/c mice	i.v. injection	70
NLG919	636	Chemotherapy (docetaxel)	Subcutaneous 4T1 tumor, BALB/c mice	i.v. injection	71
NLG919	1400	Ferroptosis (sorafenib)	Subcutaneous 4T1 tumor, BALB/c mice	i.v. injection	72
NLG919	120	Photothermal therapy	Subcutaneous 4T1 tumor, BALB/c mice	i.v. injection	73
NLG919	400	Photothermal therapy, chemotherapy (gemcitabine)	Subcutaneous Panc02, C57BL/6 mice	i.v. injection	74
NLG919	102	Photodynamic therapy	Subcutaneous 4T1 tumor, BALB/c mice	i.v. injection	75
NLG919	137.6	Photodynamic therapy	Subcutaneous 4T1 tumor, BALB/c mice	intratumoral injection	76
NLG919	600	Photodynamic therapy	Subcutaneous 4T1/CT26 tumor, BALB/c mice	i.v. injection	77
NLG919	156	Photodynamic therapy	Subcutaneous CT26 tumor, BALB/c mice	i.v. injection	78
NLG919	600	Photodynamic therapy	Subcutaneous CT26 tumor, BALB/c mice	i.v. injection	79
NLG919	390	Photodynamic therapy	Subcutaneous CT26 tumor, BALB/c mice	i.v. injection	80
NLG919	150	Photodynamic therapy	Subcutaneous B16-F10 tumor, C57BL/6 mice	i.t. injection	81
NLG919	120	Photodynamic therapy, chemotherapy (oxaliplatin)	Subcutaneous 4T1 tumor, BALB/c mice	i.v. injection	82
NLG919	120	Immunotherapy (PD-L1 antagonist)	Subcutaneous B16-F10 tumor, C57BL/6 mice	i.v. injection	83
NLG919	1000	Immunotherapy (aPD-L1)	Subcutaneous Panc02 tumor, C57BL/6 mice	i.v. injection	84
NLG919	2800	Chemotherapy (combretastatin A4), immunotherapy (PI3Kγ inhibitor)	Subcutaneous 4T1 tumor, BALB/c mice	i.p. injection	85
NLG919	150	Sonodynamic therapy,	Subcutaneous Panc02	i.v. injection	This study

		immunotherapy (aPD-L1)	tumor, C57BL/6 mice		
aPD-L1	300	Chemotherapy (doxorubicin)	Orthotopic C6 glioma, BALB/c mice	i.p. injection	86
aPD-L1	250	Chemotherapy (dimer-7-ethyl-10-hydroxycamptothecin, dimer-lonidamine)	Subcutaneous 4T1 tumor, BALB/c mice	i.p. injection	87
aPD-L1	500	Chemotherapy (doxorubicin)	Orthotopic 4T1 tumor, BALB/c mice	i.v. injection	88
aPD-L1	225	Photodynamic therapy	Subcutaneous 4T1 tumor, BALB/c mice	i.p. injection	89
aPD-L1	375	Photodynamic therapy	Subcutaneous CT26 tumor, BALB/c mice	i.p. injection	90
aPD-L1	300	Photodynamic therapy	Subcutaneous 4T1 tumor, BALB/c mice	i.p. injection	91
aPD-L1	150	Photothermal therapy	Subcutaneous 4T1 tumor, BALB/c mice	i.p. injection	92
aPD-L1	250	Photothermal therapy, chemotherapy(doxorubicin)	Subcutaneous CT26 tumor, BALB/c mice	i.v. injection	93
aPD-L1	120	Photothermal therapy, chemotherapy(doxorubicin)	Subcutaneous 4T1 tumor, BALB/c mice	i.v. injection	94
aPD-L1	150	Photodynamic therapy, chemotherapy (oxaliplatin)	Subcutaneous CT26 tumor, BALB/c mice	i.p. injection	95
aPD-L1	300	Photodynamic therapy, ferroptosis (RSL-3)	Subcutaneous B16-F10 tumor, C57BL/6 mice	i.v. injection	96
aPD-L1	135	Ferroptosis	Subcutaneous MC38 tumor, C57BL/6 mice	i.v. injection	97
aPD-L1	600	Pyroptosis	Subcutaneous B16-F10 tumor, C57BL/6 mice	i.v. injection	98
aPD-L1	225	Photodynamic therapy, immunotherapy (CpG Oligodeoxynucleotides)	Subcutaneous TUBO tumor, BALB/c mice	i.p. injection	99
aPD-L1	225	Photothermal therapy, immunotherapy (R837)	Orthotopic 4T1 tumor, BALB/c mice	i.v. injection	100
aPD-L1	400	Photodynamic therapy, chemotherapy (doxorubicin)	Subcutaneous CT26 tumor, BALB/c mice	i.v. injection	101
aPD-L1	300	Photodynamic therapy, chemotherapy (oxaliplatin)	Subcutaneous 4T1 tumor, BALB/c mice	i.p. injection	44
aPD-L1	375	Magnetic hyperthermia therapy	Orthotopic 4T1 tumor, BALB/c mice	i.p. injection	102
aPD-L1	300	Radiotherapy	Subcutaneous CT26 tumor, BALB/c mice	i.p. injection	103
aPD-L1	225	Radiotherapy, photothermal therapy	Subcutaneous 4T1 tumor, BALB/c mice	i.t. injection	104
aPD-L1	1200	Immunotherapy (aCD3, aCD28)	Orthotopic 4T1 tumor, Subcutaneous CT26 tumor, BALB/c mice	i.v. injection	105
aPD-L1	180	Sonodynamic therapy, immunotherapy (NLG919)	Subcutaneous Panc02 tumor, C57BL/6 mice	i.v. injection	This study

Supplementary Reference:

1. Harada, Y. et al. Ultrasound activation of TiO_2 in melanoma tumors. *J. Controlled Release* **149**, 190-195 (2011).
2. Deepagan, V. et al. Long-circulating Au-TiO_2 nanocomposite as a sonosensitizer for ROS-mediated eradication of cancer. *Nano Lett.* **16**, 6257-6264 (2016).
3. Huang, P. et al. Metalloporphyrin-encapsulated biodegradable nanosystems for highly efficient magnetic resonance imaging-guided sonodynamic cancer therapy. *J. Am. Chem. Soc.* **139**, 1275-1284 (2017).
4. Chen, J. et al. Oxygen-self-produced nanoplatform for relieving hypoxia and breaking resistance to sonodynamic treatment of pancreatic cancer. *ACS Nano* **11**, 12849-12862 (2017).

5. Zhu, P., Chen, Y. & Shi, J. Nanoenzyme-augmented cancer sonodynamic therapy by catalytic tumor oxygenation. *ACS Nano* **12**, 3780-3795 (2018).
6. Pan, X. et al. Metal-organic-framework-derived carbon nanostructure augmented sonodynamic cancer therapy. *Adv. Mater.* **30**, 1800180 (2018).
7. Ma, A. et al. Metalloporphyrin complex-based nanosonosensitizers for deep-tissue tumor theranostics by noninvasive sonodynamic therapy. *Small* **15**, 1804028 (2019).
8. Gong, F. et al. Ultrasmall oxygen-deficient bimetallic oxide $MnWO_X$ nanoparticles for depletion of endogenous GSH and enhanced sonodynamic cancer therapy. *Adv. Mater.* **31**, 1900730 (2019).
9. Cao, Y., Wu, T., Dai, W., Dong, H. & Zhang, X. TiO2 nanosheets with the Au nanocrystal-decorated edge for mitochondria-targeting enhanced sonodynamic therapy. *Chem. Mater.* **31**, 9105-9114 (2019).
10. Liang, K. et al. Intelligent nanocomposites with intrinsic blood-brain-barrier crossing ability designed for highly specific MR imaging and sonodynamic therapy of glioblastoma. *Small* **16**, 1906985 (2020).
11. Geng, P. et al. One responsive stone, three birds: Mn(III)-hemoporfin frameworks with glutathione-enhanced degradation, MRI, and sonodynamic therapy. *Adv. Healthcare Mater.* **10**, 2001463 (2021).
12. Geng, B. et al. Multifunctional carbon dot/MXene heterojunctions for alleviation of tumor hypoxia and enhanced sonodynamic therapy. *Carbon* **179**, 493-504 (2021).
13. Xie, J. et al. Water-soluble iridic-porphyrin complex for non-invasive sonodynamic and sono-oxidation therapy of deep tumors. *ACS Appl. Mater. Interfaces* **13**, 27934-27944 (2021).
14. Hadi, M.M. et al. Investigating the performance of a novel pH and cathepsin B sensitive, stimulus-responsive nanoparticle for optimised sonodynamic therapy in prostate cancer. *J. Controlled Release* **329**, 76-86 (2021).
15. Liu, S. et al. Multifunctional nanozyme for multimodal imaging-guided enhanced sonodynamic therapy by regulating the tumor microenvironment. *Nanoscale* **13**, 14049-14066 (2021).
16. Yang, S. et al. Graphene quantum dots with pyrrole N and pyridine N: superior reactive oxygen species generation efficiency for metal-free sonodynamic tumor therapy. *Small* **17**, 2004867 (2021).
17. Yang, K. et al. A hypoxia responsive nanoassembly for tumor specific oxygenation and enhanced sonodynamic therapy. *Biomaterials* **275**, 120822 (2021).
18. Duo, Y. et al. Patient-derived microvesicles/AIE luminogen hybrid system for personalized sonodynamic cancer therapy in patient-derived xenograft models. *Biomaterials* **272**, 120755 (2021).
19. Yuan, M. et al. A robust oxygen-carrying hemoglobin-based natural sonosensitizer for sonodynamic cancer therapy. *Nano Lett.* **21**, 6042-6050 (2021).
20. Liang, S. et al. Conferring Ti-based MOFs with defects for enhanced sonodynamic cancer therapy. *Adv. Mater.* **33**, 2100333 (2021).
21. Liang, S. et al. A robust narrow bandgap vanadium tetrasulfide sonosensitizer optimized by charge separation engineering for enhanced sonodynamic cancer therapy. *Adv. Mater.* **33**, 2101467 (2021).
22. Bao, Y. et al. Erythrocyte membrane-camouflaged PCN-224 nanocarriers integrated with platinum nanoparticles and glucose oxidase for enhanced tumor sonodynamic therapy and synergistic starvation therapy. *ACS Appl. Mater. Interfaces* **13**, 24532-24542 (2021).
23. Sheng, Y. et al. Magnetically responsive microbubbles as delivery vehicles for targeted sonodynamic and antimetabolite therapy of pancreatic cancer. *J. Controlled Release* **262**, 192-200 (2017).

24. Li, Z. et al. Synergistic sonodynamic/chemotherapeutic suppression of hepatocellular carcinoma by targeted biodegradable mesoporous nanosonosensitizers. *Adv. Funct. Mater.* **28**, 1800145 (2018).
25. Kim, S. et al. Drug-loaded titanium dioxide nanoparticle coated with tumor targeting polymer as a sonodynamic chemotherapeutic agent for anti-cancer therapy. *Nanomedicine* **24**, 102110 (2020).
26. Liang, S. et al. A novel Pt-TiO_2 heterostructure with oxygen-deficient layer as bilaterally enhanced sonosensitizer for synergistic chemo-sonodynamic cancer therapy. *Adv. Funct. Mater.* **30**, 1908598 (2020).
27. Beguin, E. et al. Magnetic microbubble mediated chemo-sonodynamic therapy using a combined magnetic-acoustic device. *J. Controlled Release* **317**, 23-33 (2020).
28. Li, W.-P., Su, C.-H., Chang, Y.-C., Lin, Y.-J. & Yeh, C.-S. Ultrasound-induced reactive oxygen species mediated therapy and imaging using a fenton reaction activable polymersome. *ACS Nano* **10**, 2017-2027 (2016).
29. Fu, J., Li, T., Zhu, Y. & Hao, Y. Ultrasound ‐ activated oxygen and ROS generation nanosystem systematically modulates tumor microenvironment and sensitizes sonodynamic therapy for hypoxic solid tumors. *Adv. Funct. Mater.* **29**, 1906195 (2019).
30. Lin, X. et al. An ultrasound activated vesicle of janus Au-MnO nanoparticles for promoted tumor penetration and sono-chemodynamic therapy of orthotopic liver cancer. *Angew. Chem. Int. Ed.* **59**, 1682-1688 (2020).
31. Zhong, X. et al. GSH-depleted $PtCu_3$ nanocages for chemodynamic-enhanced sonodynamic cancer therapy. *Adv. Funct. Mater.* **30**, 1907954 (2020).
32. Xu, W. et al. Engineering Janus Chemoreactive Nanosonosensitizers for Bilaterally Augmented Sonodynamic and Chemodynamic Cancer Nanotherapy. *Adv. Funct. Mater.* **31**, 2103134 (2021).
33. Xu, Q. et al. Manganese porphyrin-based metal-organic framework for synergistic sonodynamic therapy and ferroptosis in hypoxic tumors. *Theranostics* **11**, 1937 (2021).
34. Nomikou, N. et al. A versatile, stimulus-responsive nanoparticle-based platform for use in both sonodynamic and photodynamic cancer therapy. *Acta Biomater.* **49**, 414-421 (2017).
35. Dai, C., Zhang, S., Liu, Z., Wu, R. & Chen, Y. Two-dimensional graphene augments nanosonosensitized sonocatalytic tumor eradication. *ACS Nano* **11**, 9467-9480 (2017).
36. Han, X. et al. Oxygen-deficient black titania for synergistic/enhanced sonodynamic and photoinduced cancer therapy at near infrared-II biowindow. *ACS Nano* **12**, 4545-4555 (2018).
37. Liang, S. et al. Intelligent hollow Pt-CuS janus architecture for synergistic catalysis-enhanced sonodynamic and photothermal cancer therapy. *Nano Lett.* **19**, 4134-4145 (2019).
38. He, Y. et al. Multifunctional polypyrrole-coated mesoporous TiO_2 nanocomposites for photothermal, sonodynamic, and chemotherapeutic treatments and dual-modal ultrasound/photoacoustic imaging of tumors. *Adv. Healthcare Mater.* **8**, 1801254 (2019).
39. Liu, Z., Li, J., Chen, W., Liu, L. & Yu, F. Light and sound to trigger the Pandora's box against breast cancer: A combination strategy of sonodynamic, photodynamic and photothermal therapies. *Biomaterials* **232**, 119685 (2020).
40. Li, C.-Q. et al. In situ synthesis of multifunctional tellurium nanorods stabilized by polypeptide-engineered for photothermal-sonodynamic combination therapy of tumors. *Chem. Eng. J.* **417**, 127989 (2021).
41. Li, G. et al. Titanium carbide nanosheets with defect structure for photothermal-enhanced sonodynamic therapy. *Bioact. Mater.* **8**, 409-419 (2022).
42. Shen, J. et al. Cancer cell membrane camouflaged iridium complexes functionalized black-titanium nanoparticles for hierarchical-targeted synergistic NIR-II photothermal and sonodynamic therapy. *Biomaterials* **275**, 120979 (2021).

43. Wang, X. et al. Liquid exfoliation of TiN nanodots as novel sonosensitizers for photothermal-enhanced sonodynamic therapy against cancer. *Nano Today* **39**, 101170 (2021).
44. Yue, W. et al. Checkpoint blockade and nanosonosensitizer-augmented noninvasive sonodynamic therapy combination reduces tumour growth and metastases in mice. *Nat. Commun.* **10**, 2025 (2019).
45. Zhang, D. et al. Ultrasound-driven biomimetic nanosystem suppresses tumor growth and metastasis through sonodynamic therapy, CO therapy, and indoleamine 2, 3-dioxygenase inhibition. *ACS Nano* **14**, 8985-8999 (2020).
46. Wei, X. et al. Homology and immune checkpoint dual-targeted sonocatalytic nanoagents for enhancing sonodynamic tumor therapy. *ACS Appl. Mater. Interfaces* **13**, 32810-32822 (2021).
47. Yin, Y. et al. Continuous inertial cavitation evokes massive ROS for reinforcing sonodynamic therapy and immunogenic cell death against breast carcinoma. *Nano Today* **36**, 101009 (2021).
48. Zhu, W. et al. Sonodynamic therapy with immune modulatable two-dimensional coordination nanosheets for enhanced anti-tumor immunotherapy. *Nano Res.* **14**, 212-221 (2021).
49. Lin, X. et al. Nanosonosensitizer-augmented sonodynamic therapy combined with checkpoint blockade for cancer immunotherapy. *Int. J. Nanomed.* **16**, 1889-1899 (2021).
50. Wang, M. et al. A multifunctional nanovaccine based on L-arginine-loaded black mesoporous titania: ultrasound-triggered synergistic cancer sonodynamic therapy/gas therapy/immunotherapy with remarkably enhanced efficacy. *Small* **17**, 2005728 (2021).
51. Ji, C. et al. Mitochondria-targeted and ultrasound-responsive nanoparticles for oxygen and nitric oxide codelivery to reverse immunosuppression and enhance sonodynamic therapy for immune activation. *Theranostics* **11**, 8587-8604 (2021).
52. Chen, H. et al. Noninvasively immunogenic sonodynamic therapy with manganese protoporphyrin liposomes against triple-negative breast cancer. *Biomaterials* **269**, 120639 (2021).
53. Tan, X. et al. Transformable nanosensitizer with tumor microenvironment ‐ activated sonodynamic process and calcium release for enhanced cancer immunotherapy. *Angew. Chem. Int. Ed.* **60**, 14051-14059 (2021).
54. Zhan, G. et al. Biomimetic sonodynamic therapy-nanovaccine integration platform potentiates Anti-PD-1 therapy in hypoxic tumors. *Nano Today* **38**, 101195 (2021).
55. Huang, J. et al. Nanodrug with dual-sensitivity to tumor microenvironment for immuno-sonodynamic anti-cancer therapy. *Biomaterials* **269**, 120636 (2021).
56. Fu, S. et al. Catalytically active $CoFe_2O_4$ nanoflowers for augmented sonodynamic and chemodynamic combination therapy with elicitation of robust immune response. *ACS Nano* **15**, 11953-11969 (2021).
57. Chen, Y. et al. An immunostimulatory dual-functional nanocarrier that improves cancer immunochemotherapy. *Nat. Commun.* **7**, 13443 (2016).
58. Sun, J.-j. et al. Programmable co-delivery of the immune checkpoint inhibitor NLG919 and chemotherapeutic doxorubicin via a redox-responsive immunostimulatory polymeric prodrug carrier. *Acta Pharmacol. Sin.* **38**, 823-834 (2017).
59. Meng, X. et al. Combinatorial antitumor effects of indoleamine 2,3-dioxygenase inhibitor NLG919 and paclitaxel in a murine B16-F10 melanoma model. *Int. J. Immunopathol. Pharmacol.* **30**, 215-226 (2017).
60. Feng, B. et al. Binary cooperative prodrug nanoparticles improve immunotherapy by synergistically modulating immune tumor microenvironment. *Adv. Mater.* **30**, 1803001 (2018).
61. Xu, J. et al. NLG919/cyclodextrin complexation and anti-cancer therapeutic benefit as a potential immunotherapy in combination with paclitaxel. *Eur. J. Pharm. Sci.* **138**, 105034 (2019).

62. Gao, J., Deng, F. & Jia, W. Inhibition of indoleamine 2,3-dioxygenase enhances the therapeutic efficacy of immunogenic chemotherapeutics in breast cancer. *J. Breast. Cancer* **22**, 196-209 (2019).
63. Qiao, H. et al. Folated pH-degradable nanogels for the simultaneous delivery of docetaxel and an IDO1-inhibitor in enhancing cancer chemo-immunotherapy. *Biomater. Sci.* **7**, 2749-2758 (2019).
64. Sun, J. et al. Triple drugs co-delivered by a small gemcitabine-based carrier for pancreatic cancer immunochemotherapy. *Acta Biomater.* **106**, 289-300 (2020).
65. Dai, L. et al. Programmable prodrug micelle with size-shrinkage and charge-reversal for chemotherapy-improved IDO immunotherapy. *Biomaterials* **241**, 119901 (2020).
66. Shen, F. et al. Oxaliplatin-/NLG919 prodrugs-constructed liposomes for effective chemo-immunotherapy of colorectal cancer. *Biomaterials* **255**, 120190 (2020).
67. Zhao, L. et al. Self-delivery oxidative stress amplifier for chemotherapy sensitized immunotherapy. *Biomaterials* **275**, 120970 (2021).
68. Ding, Y. et al. Plasmon-driven catalytic chemotherapy augments cancer immunotherapy through induction of immunogenic cell death and blockage of IDO pathway. *Adv. Mater.* **33**, 2102188 (2021).
69. Luo, K. et al. Charge convertible biomimetic micellar nanoparticles for enhanced melanoma-targeted therapy through tumor cells and tumor-associated macrophages dual chemotherapy with IDO immunotherapy. *Chem. Eng. J.* **412**, 128659 (2021).
70. Zhu, Y. et al. $CaCO_3$-assisted preparation of pH-responsive immune-modulating nanoparticles for augmented chemo-immunotherapy. *Nano-Micro Lett.* **13**, 29 (2021).
71. Lang, T. et al. Ternary regulation of tumor microenvironment by heparanase-sensitive micelle-loaded monocytes improves chemo-immunotherapy of metastatic breast cancer. *Adv. Funct. Mater.* **31**, 2007402 (2021).
72. Zuo, T. et al. pH-sensitive molecular-switch-containing polymer nanoparticle for breast cancer therapy with ferritinophagy-cascade ferroptosis and tumor immune activation. *Adv. Healthcare Mater.* **10**, 2100683 (2021).
73. Peng, J. et al. Photosensitizer micelles together with IDO inhibitor enhance cancer photothermal therapy and immunotherapy. *Adv. Sci.* **5**, 1700891 (2018).
74. Sun, J. et al. Tumor size-dependent abscopal effect of polydopamine-coated all-in-one nanoparticles for immunochemo-photothermal therapy of early-and late-stage metastatic cancer. *Biomaterials* **269**, 120629 (2021).
75. Huang, Z. et al. Enhanced cancer therapy through synergetic photodynamic/immune checkpoint blockade mediated by a liposomal conjugate comprised of porphyrin and IDO inhibitor. *Theranostics* **9**, 5542-5557 (2019).
76. Xing, L. et al. Hypoxia alleviation-triggered enhanced photodynamic therapy in combination with IDO inhibitor for preferable cancer therapy. *Biomaterials* **206**, 170-182 (2019).
77. Gao, A. et al. Sheddable prodrug vesicles combating adaptive immune resistance for improved photodynamic immunotherapy of cancer. *Nano Lett.* **20**, 353-362 (2019).
78. Hu, X. et al. Supramolecular prodrug nanovectors for active tumor targeting and combination immunotherapy of colorectal cancer. *Adv. Sci.* **7**, 1903332 (2020).
79. Hou, B. et al. Engineering stimuli-activatable boolean logic prodrug nanoparticles for combination cancer immunotherapy. *Adv. Mater.* **32**, 1907210 (2020).
80. Zhao, L.-P. et al. Self-delivery photo-immune stimulators for photodynamic sensitized tumor immunotherapy. *ACS Nano* **14**, 17100-17113 (2020).
81. Yang, X. et al. Nanoconjugates to enhance PDT-mediated cancer immunotherapy by targeting

the indoleamine-2, 3-dioxygenase pathway. *J. Nanobiotechnol.* **19**, 182 (2021).
82. Feng, B. et al. Self-amplified drug delivery with light-inducible nanocargoes to enhance cancer immunotherapy. *Adv. Mater.* **31**, 1902960 (2019).
83. Cheng, K. et al. Sequentially responsive therapeutic peptide assembling nanoparticles for dual-targeted cancer immunotherapy. *Nano Lett.* **18**, 3250-3258 (2018).
84. Han, X. et al. Modularly designed peptide nanoprodrug augments antitumor immunity of PD-L1 checkpoint blockade by targeting indoleamine 2,3-dioxygenase. *J. Am. Chem. Soc.* **142**, 2490-2496 (2020).
85. Qin, H. et al. PI3Kgamma inhibitor attenuates immunosuppressive effect of poly (l-glutamic acid)-combretastatin A4 conjugate in metastatic breast cancer. *Adv. Sci.* **6**, 1900327 (2019).
86. Ruan, S. et al. Aggregable nanoparticles-enabled chemotherapy and autophagy inhibition combined with anti-PD-L1 antibody for improved glioma treatment. *Nano Lett.* **19**, 8318-8332 (2019).
87. Yang, X. et al. Tumor microenvironment-responsive dual drug dimer-loaded pegylated bilirubin nanoparticles for improved drug delivery and enhanced immune-chemotherapy of breast cancer. *Adv. Funct. Mater.* **29**, 1901896 (2019).
88. Shao, D. et al. Biomimetic diselenide-bridged mesoporous organosilica nanoparticles as an X-ray-responsive biodegradable carrier for chemo-immunotherapy. *Adv. Mater.* **32**, 2004385 (2020).
89. Duan, X. et al. Photodynamic therapy mediated by nontoxic core-shell nanoparticles synergizes with immune checkpoint blockade to elicit antitumor immunity and antimetastatic effect on breast cancer. *J. Am. Chem. Soc.* **138**, 16686-16695 (2016).
90. Lan, G. et al. Nanoscale metal-organic framework overcomes hypoxia for photodynamic therapy primed cancer immunotherapy. *J. Am. Chem. Soc.* **140**, 5670-5673 (2018).
91. Wang, R. et al. Sustained antitumor immunity based on persistent luminescence nanoparticles for cancer immunotherapy. *Adv. Funct. Mater.* **31**, 2106884 (2021).
92. Wang, R. et al. Surface-functionalized modified copper sulfide nanoparticles enhance checkpoint blockade tumor immunotherapy by photothermal therapy and antigen capturing. *ACS Appl. Mater. Interfaces* **11**, 13964-13972 (2019).
93. Wen, Y. et al. Photothermal-chemotherapy integrated nanoparticles with tumor microenvironment response enhanced the induction of immunogenic cell death for colorectal cancer efficient treatment. *ACS Appl. Mater. Interfaces* **11**, 43393-43408 (2019).
94. Hu, L. et al. The potentiated checkpoint blockade immunotherapy by ROS-responsive nanocarrier-mediated cascade chemo-photodynamic therapy. *Biomaterials* **223**, 119469 (2019).
95. He, C. et al. Core-shell nanoscale coordination polymers combine chemotherapy and photodynamic therapy to potentiate checkpoint blockade cancer immunotherapy. *Nat. Commun.* **7**, 12499 (2016).
96. Song, R. et al. Acidity-activatable dynamic nanoparticles boosting ferroptotic cell death for immunotherapy of cancer. *Adv. Mater.* **33**, 2101155 (2021).
97. Liang, H. et al. Renal clearable ultrasmall single-crystal Fe nanoparticles for highly selective and effective ferroptosis therapy and immunotherapy. *J. Am. Chem. Soc.* **143**, 15812-15823 (2021).
98. Xiong, H. et al. Inspired epigenetic modulation synergy with adenosine inhibition elicits pyroptosis and potentiates cancer immunotherapy. *Adv. Funct. Mater.* **31**, 2100007 (2021).
99. Ni, K. et al. A nanoscale metal-organic framework to mediate photodynamic therapy and deliver CpG oligodeoxynucleotides to enhance antigen presentation and cancer immunotherapy. *Angew. Chem. Int. Ed.* **59**, 1108-1112 (2020).
100. Ge, R. et al. Photothermal-activatable Fe_3O_4 superparticle nanodrug carriers with PD-L1

immune checkpoint blockade for anti-metastatic cancer immunotherapy. *ACS Appl. Mater. Interfaces* **10**, 20342-20355 (2018).
101. Choi, J. et al. Visible-light-triggered prodrug nanoparticles combine chemotherapy and photodynamic therapy to potentiate checkpoint blockade cancer immunotherapy. *ACS Nano* **15**, 12086-12098 (2021).
102. Liu, X. et al. Ferrimagnetic vortex nanoring-mediated mild magnetic hyperthermia imparts potent immunological effect for treating cancer metastasis. *ACS Nano* **13**, 8811-8825 (2019).
103. Ni, K. et al. Nanoscale metal-organic frameworks enhance radiotherapy to potentiate checkpoint blockade immunotherapy. *Nat. Commun.* **9**, 2351 (2018).
104. Dong, X. et al. A heterojunction structured $WO_{2.9}$-WSe_2 nanoradiosensitizer increases local tumor ablation and checkpoint blockade immunotherapy upon low radiation dose. *ACS Nano* **14**, 5400-5416 (2020).
105. Chiang, C.-S. et al. Combination of fucoidan-based magnetic nanoparticles and immunomodulators enhances tumour-localized immunotherapy. *Nat. Nanotechnol.* **13**, 746-754 (2018).
