# Supplementary material for: Precision cancer sono-immunotherapy using deep-tissue activatable semiconducting polymer immunomodulatory nanoparticles
Source: Nat Commun. 2022 Jul 12;13:4032. doi: 10.1038/s41467-022-31551-6 (PMC9276830; doi:10.1038/s41467-022-31551-6)
Supplement: Supplementary file 2 — Reporting Summary [file 41467_2022_31551_MOESM2_ESM.pdf]

nature portfolio

Corresponding author(s): Kanyi Pu

Last updated by author(s): Jun 22, 2022

Reporting Summary

Nature Portfolio wishes to improve the reproducibility of the work that we publish. This form provides structure for consistency and transparency in reporting. For further information on Nature Portfolio policies, see our Editorial Policies and the Editorial Policy Checklist.

Statistics

For all statistical analyses, confirm that the following items are present in the figure legend, table legend, main text, or Methods section.

n/a	Confirmed	
☐	☒	The exact sample size (*n*) for each experimental group/condition, given as a discrete number and unit of measurement
☐	☒	A statement on whether measurements were taken from distinct samples or whether the same sample was measured repeatedly
☐	☒	The statistical test(s) used AND whether they are one- or two-sided<br>*Only common tests should be described solely by name; describe more complex techniques in the Methods section.*
☒	☐	A description of all covariates tested
☒	☐	A description of any assumptions or corrections, such as tests of normality and adjustment for multiple comparisons
☐	☒	A full description of the statistical parameters including central tendency (e.g. means) or other basic estimates (e.g. regression coefficient) AND variation (e.g. standard deviation) or associated estimates of uncertainty (e.g. confidence intervals)
☐	☒	For null hypothesis testing, the test statistic (e.g. *F*, *t*, *r*) with confidence intervals, effect sizes, degrees of freedom and *P* value noted<br>*Give P values as exact values whenever suitable.*
☒	☐	For Bayesian analysis, information on the choice of priors and Markov chain Monte Carlo settings
☒	☐	For hierarchical and complex designs, identification of the appropriate level for tests and full reporting of outcomes
☒	☐	Estimates of effect sizes (e.g. Cohen's *d*, Pearson's *r*), indicating how they were calculated

Our web collection on statistics for biologists contains articles on many of the points above.

Software and code

Policy information about availability of computer code

Data collection	UV Probe software (version 2.3) and fluorolog software (version 3.2) were used to acquire UV and fluorescence data. Agilentopenlab control panel was used to acquire HPLC data. Micromanager was used to acquire histological data. ZEN blue software (version 2.3) was used forconfocal microscopy to acquire fluorescence images. Living Image Software (version 4.3) was used for IVIS Imaging Systems to acquire fluorescence imaging data. Fortessa X20 flow cytometer was used to collect flow cytometry data.
Data analysis	NMR spectra were analyzed using Mestre Nova LITE v5.2.5-4119 software(Mestre lab Research S.L.). Imaging data were analyzed using the Living Image 4.3 Software(Perkinelmer). Confocal fluorescence microscopy imaging data were analyzed using Zen 2.3 blue edition (Carl Zeiss). Flow cytometry data was analyzed using FlowJo software (version 10.7.2). All statistical calculations were performed using Graphpad Prism 6.0 (Graphpad Software Inc.).

For manuscripts utilizing custom algorithms or software that are central to the research but not yet described in published literature, software must be made available to editors and reviewers. We strongly encourage code deposition in a community repository (e.g. GitHub). See the Nature Portfolio guidelines for submitting code & software for further information.

Data

Policy information about availability of data

All manuscripts must include a data availability statement. This statement should provide the following information, where applicable:

- Accession codes, unique identifiers, or web links for publicly available datasets
- A description of any restrictions on data availability
- For clinical datasets or third party data, please ensure that the statement adheres to our policy

The RNA sequencing data has been deposited in the Genome Sequence Archive (GSA) database under the accession code CRA007151 [https://bigd.big.ac.cn/gsa/

browse/CRA007151]. The remaining data are available within the Article, Supplementary Information or Source Data file.

Field-specific reporting

Please select the one below that is the best fit for your research. If you are not sure, read the appropriate sections before making your selection.

[x] Life sciences [ ] Behavioural & social sciences [ ] Ecological, evolutionary & environmental sciences

For a reference copy of the document with all sections, see nature.com/documents/nr-reporting-summary-flat.pdf

Life sciences study design

All studies must disclose on these points even when the disclosure is negative.

Sample size	G*power analysis was used to calculate and ensure the sample sizes fulfill adequate power (p>0.8). According to the experimental data and sample size (n) and P value were calculated and the power was then calculated. If it is more than 80%, demonstrating the sample size is adequate. All sample sizes, statistical tests and P values are indicated in the figure legends.
Data exclusions	No data was excluded from analysis.
Replication	All experiments were repeated at least three independent experiments with similar results. Experiments were reproduced to reliably support conclusions stated in the manuscript.
Randomization	The mice and cells were randomly allocated into experimental groups for further treatments.
Blinding	Investigators were blinded to group allocation during experiments. Investigators performing in vivo fluorescence imaging, SPECT imaging, CT imaging, tumor size measurement, survival monitoring, tissue/blood collection and section staining were blinded to saline and treatment groups.

Reporting for specific materials, systems and methods

We require information from authors about some types of materials, experimental systems and methods used in many studies. Here, indicate whether each material, system or method listed is relevant to your study. If you are not sure if a list item applies to your research, read the appropriate section before selecting a response.

Materials & experimental systems

n/a	Involved in the study	
[ ]	[x]	Antibodies
[ ]	[x]	Eukaryotic cell lines
[x]	[ ]	Palaeontology and archaeology
[ ]	[x]	Animals and other organisms
[x]	[ ]	Human research participants
[x]	[ ]	Clinical data
[x]	[ ]	Dual use research of concern

Methods

n/a	Involved in the study	
[x]	[ ]	ChIP-seq
[ ]	[x]	Flow cytometry
[x]	[ ]	MRI-based neuroimaging

Antibodies

Antibodies used

The following antibodies were used for flow cytometry. They are listed as antigen first, followed by supplier, catalog number and clone/lot number as applicable.
CD45-BV605 (Biolegend, cat. no. 103140, clone 30-F11), CD11c-FITC (Biolegend, cat. no. 117306, clone N418), CD80-PE (Biolegend, cat. no. 104708, clone 16-10A1), CD86-APC (Biolegend, cat. no. 105114, clone PO3), CD3-APC/Cyanine7 (Biolegend, cat. no. 100222, clone 17A2), CD4-FITC (Biolegend, cat. no. 100406, clone GK1.5), CD8-PE (Biolegend, cat. no. 140408, clone 53-5.8), Foxp3-PE (Biolegend, cat. no. 126404, clone MF-14), CD25-APC (Biolegend, cat. no. 101910, clone 3C7), CD44-APC (Biolegend, cat. no. 103011, clone IM7), CD26L-PerCP/Cyanine5.5 (Biolegend, cat. no. 104431, clone MEL-14), Brilliant Violet 605™ Rat IgG2b, κ Isotype Ctrl (Biolegend, cat. no. 400650, clone RTK4530), APC/Cyanine7 Rat IgG2b, κ Isotype Ctrl (Biolegend, cat. no. 400624, clone RTK4530), FITC Rat IgG2b, κ Isotype Ctrl (Biolegend, cat. no. 400605, clone RTK4530), PE Rat IgG2b, κ Isotype Ctrl (Biolegend, cat. no. 400608, clone RTK4530), APC Rat IgG2b, κ Isotype Ctrl (Biolegend, cat. no. 400612, clone RTK4530), PerCP/Cyanine5.5 Rat IgG2a, κ Isotype Ctrl (Biolegend, cat. no. 400531, clone RTK2758), FITC Armenian Hamster IgG Isotype Ctrl (Biolegend, cat. no. 400905, clone HTK888), and PE Armenian Hamster IgG Isotype Ctrl (Biolegend, cat. no. 400907, clone HTK888).

The following antibodies were used for immunofluorescence staining. They are listed as antigen first, followed by supplier, catalog number and clone/lot number as applicable.
Anti-calreticulin antibody (Abcam Inc., cat. no. ab227444), anti-HMGB1 antibody (Abcam Inc., cat. no. ab79823), goat anti-rabbit IgG H&L (Alexa Fluor® 488) (Abcam Inc., cat. no. ab150077), anti-PD-L1 antibody (Abcam Inc., cat. no. ab213480), FITC anti-Granzyme B (Biolegend, cat. no. 515403, clone GB11), FITC IFN-γ (Biolegend, cat. no. 505805, clone XMG1.2), CD3-APC/Cyanine7 (Biolegend, cat. no. 100222, clone 17A2), CD4-APC/Cyanine7 (Biolegend, cat. no. 100414, clone GK1.5), and CD8b.2-APC/Cyanine7 (Biolegend, cat.

no. 140422 , clone 53-5.8).

The following antibodies were used for immunohistochemical staining. They are listed as antigen first, followed by supplier, catalog number and clone/lot number as applicable.
Anti-CD3 antibody (Abcam Inc., cat. no. ab16669), anti-CD8α antibody (Abcam Inc., cat. no. ab217344), and goat anti-rabbit IgG H&L (HRP) (Abcam Inc., cat. no. ab205718).

Validation

All antibodies were used in this study according to the profile of manufacturers. Antibody validation for flow cytometry, immunohistochemical and immunofluorescence staining was validated by the supplier and confirmed. Specifically, the validation statements of primary antibodies can be found on the manufacturer's website:
1. https://www.abcam.com/calreticulin-antibody-ab227444.html
2. https://www.abcam.com/hmgb1-antibody-epr3507-ab79823.html
3. https://www.abcam.com/pd-l1-antibody-epr20529-ab213480.html
4. https://www.abcam.com/cd3-antibody-sp7-ab16669.html
5. https://www.abcam.com/cd8-alpha-antibody-epr21769-ab217344.html

Eukaryotic cell lines

Policy information about cell lines

Cell line source(s)

The murine pancreatic cell line Panc02 cells were purchased from National Infrastructure of Cell Line Resource (NICR). Rabbit VX2 liver cancer cells (MZ-0769) were obtained from Ningbo Mingzhou Biological Technology Co., Ltd.

Authentication

It was authenticated by the supplier using STR analysis.

Mycoplasma contamination

No contamination was detected by the supplier using Hoechst DNA stain method, agar culture method, PCR-based assay.

Commonly misidentified lines
(See ICLAC register)

No commonly misidentified cell lines were used.

Animals and other organisms

Policy information about studies involving animals; ARRIVE guidelines recommended for reporting animal research

Laboratory animals

Female C57BL/6 mice (4-6 weeks) and female new Zealand white rabbits (age: 9-12 weeks, weight: 2.0-2.5 kg) were purchased from Shanghai Slac Laboratory Animal Center. The mice were housed with a 12 h/12 h light/dark cycle (temperature: 20-25 °C, humidity: 50-65 %) and fed with food and water ad libitum.

Wild animals

The study did not involve wild animals.

Field-collected samples

The study did not involve samples collected from field.

Ethics oversight

Animal experiments were performed according to the protocols authorized by the Institutional Animal Care and Use Committee (IACUC) at Tongji University.

Note that full information on the approval of the study protocol must also be provided in the manuscript.

Flow Cytometry

Plots

Confirm that:

- [x] The axis labels state the marker and fluorochrome used (e.g. CD4-FITC).
- [x] The axis scales are clearly visible. Include numbers along axes only for bottom left plot of group (a 'group' is an analysis of identical markers).
- [x] All plots are contour plots with outliers or pseudocolor plots.
- [x] A numerical value for number of cells or percentage (with statistics) is provided.

Methodology

Sample preparation

The collected tissues were cut into small pieces and grinded into tissue suspension in an ice box. The tissue suspension was filtered through 70-µm cell strainers to obtain single-cell suspension, followed by treatments with red blood cell lysis buffer or lymphocyte separation medium and washing with PBS to separate lymphocytes.

Instrument

Fortessa X20 flow cytometer (BD Biosciences, USA)

Software

FlowJo software (version 10.7.2)

Cell population abundance

No sorting was performed.

Gating strategy	The cells were first gated on FSC/SSC and then gated for singlets (FSC-H and FSC- A), and stained using DAPI or Zombie Aqua™ (Biolegend, cat. no. 423102) to exclude dead cells and took only the live leukocytes (CD45-BV605). Surface antigen gating was performed on the live cell population.

☒ Tick this box to confirm that a figure exemplifying the gating strategy is provided in the Supplementary Information.
