# Supplementary material for: Precision cancer sono-immunotherapy using deep-tissue activatable semiconducting polymer immunomodulatory nanoparticles
Source: Nat Commun. 2022 Jul 12;13:4032. doi: 10.1038/s41467-022-31551-6 (PMC9276830; doi:10.1038/s41467-022-31551-6)
Supplement: Supplementary file 3 — Source Data [file 41467_2022_31551_MOESM3_ESM.zip › Source Data/Figures Source Data/Source Data/Legends for Source Data.docx]

**Source Data Fig. 2:** Data for UV-vis spectra, ESR spectra, sonodynamic property and stability.

**Source Data Fig. 3:** Data for zeta potentials, sizes, hemolysis percentages, deep-tissue sonodynamic activation, drug release and binding activity assay.

**Source Data Fig. 4:** Data for tumor volumes, survival, flow cytometry analysis and gene expression assay.

**Source Data Fig. 5:** Data for tumor volumes and survival of mice, radiolabeling stability, SPECT imaging signal intensity, and tumor volumes and survival of rabbits.

**Source Data Fig. 6:** Data for CD3^+^CD4^+^ T and CD3^+^CD8^+^ T populations and serum levels of ALT and AST.
