# Supplementary material for: Precision cancer sono-immunotherapy using deep-tissue activatable semiconducting polymer immunomodulatory nanoparticles
Source: Nat Commun. 2022 Jul 12;13:4032. doi: 10.1038/s41467-022-31551-6 (PMC9276830; doi:10.1038/s41467-022-31551-6)
Supplement: Supplementary file 3 — Source Data [file 41467_2022_31551_MOESM3_ESM.zip › Source Data/Supplementary Source Data/Supplementary Dataset/Legends for Supplementary Dataset.docx]

**Supplementary Dataset Fig. 1:** Data for hydrodynamic diameters.

**Supplementary Dataset Fig. 2:** Data for fluorescence spectra.

**Supplementary Dataset Fig. 3:** Data for ESR spectra.

**Supplementary Dataset Fig. 4:** Data for fluorescence enhancement of SOSG.

**Supplementary Dataset Fig. 5:** Data for ESR spectra.

**Supplementary Dataset Fig. 6:** Data for UV-vis absorption spectra.

**Supplementary Dataset Fig. 11:** Data for gel permeation chromatography curve, UV-vis absorption spectrum and fluorescence spectrum.

**Supplementary Dataset Fig. 22:** Data for hydrodynamic sizes.

**Supplementary Dataset Fig. 23:** Data for UV-vis absorption and fluorescence spectra.

**Supplementary Dataset Fig. 24:** Data for in vitro cell viability.

**Supplementary Dataset Fig. 25:** Data for ESR spectra.

**Supplementary Dataset Fig. 26:** Data for drug release profiles.

**Supplementary Dataset Fig. 27:** Data for NIR fluorescence intensity.

**Supplementary Dataset Fig. 29:** Data for in vivo biodistribution.

**Supplementary Dataset Fig. 30:** Data for mean fluorescence intensity of SOSG.

**Supplementary Dataset Fig. 31:** Data for mean fluorescence intensity of CRT and HMGB1 staining and relative ATP levels.

**Supplementary Dataset Fig. 32:** Data for mean fluorescence intensity of PD-L1 staining.

**Supplementary Dataset Fig. 35:** Data for relative tumor volumes of primary and distant tumors.

**Supplementary Dataset Fig. 36:** Data for survival of mice.

**Supplementary Dataset Fig. 40:** Data for populations of CD80^+^CD86^+^ DCs.

**Supplementary Dataset Fig. 41:** Data for populations of CD3^+^CD8^+^ T cells in primary tumors and distant tumors.

**Supplementary Dataset Fig. 42:** Data for populations of T_reg_ cells in primary tumors and distant tumors.

**Supplementary Dataset Fig. 44:** Data for mean fluorescence intensity of IFN-γ and Granzyme B staining.

**Supplementary Dataset Fig. 45:** Data for populations of CD3^+^CD8^+^ T cells.

**Supplementary Dataset Fig. 51:** Data for relative mean fluorescence intensity of CD3 staining.

**Supplementary Dataset Fig. 53:** Data for relative mean fluorescence intensity of CD4 staining.

**Supplementary Dataset Fig. 55:** Data for relative mean fluorescence intensity of CD8 staining.

**Supplementary Dataset Fig. 56:** Data for cytokine levels.

**Supplementary Dataset Fig. 57:** Data for levels of ALP, CREA, UREA, and GGT.

**Supplementary Dataset Fig. 58:** Data for blood routine analysis.

**Supplementary Dataset Fig. 59:** Data for mouse body weight.
